# Supplementary material for: Web-based self-management support for people with type 2 diabetes (HeLP-Diabetes): randomised controlled trial in English primary care
Source: BMJ Open. 2017 Sep 27;7(9):e016009. doi: 10.1136/bmjopen-2017-016009 (PMC5623569; doi:10.1136/bmjopen-2017-016009)
Supplement: Supplementary file 1 [file bmjopen-2017-016009supp001.pdf]

Appendix 1: description of the intervention.[1]

Title: Healthy Living for People with type 2 Diabetes (HeLP-Diabetes): a web-based self-management programme for people with type 2diabetes.

Rationale

The aim of HeLP-Diabetes was to enable people with type 2 diabetes to lead healthier lives and improve overall quality of life. In order to do this, we adopted a strong theoretical framework, which included:

1. The Corbin and Strauss model of living with a long term condition,[2] which determined the overall scope of the intervention;
2. Normalisation Process Theory,[3] to maximise likelihood of successful implementation of the intervention into routine health care
3. A taxonomy of behaviour change techniques,[4] to guide the content of the behaviour change modules;
4. Finally, we worked within the paradigm of evidence-based medicine, ensuring all content was congruent with current best evidence. This applied both to the medical content (e.g. information about treatments) and to the delivery of the intervention (e.g. evidence about maximising acceptability, uptake and adherence).

Self-management of a long term condition is a complex process, which Corbin and Strauss characterised as requiring medical, emotional and role management.[2] Medical management includes behaviour change, such as eating healthily, being physically active, stopping smoking and taking medication. Working with health professionals and the health care system (e.g. keeping appointments, remembering to schedule check-ups or regular monitoring tests) is also included in medical management. Equally important, and often just as challenging for individuals concerned, are emotional management and role management. Emotional management refers to managing the complex negative emotions that result from being diagnosed with a long term condition, such as depression, anger, guilt, shame and a sense of stigma. Role management requires adjusting to the change in biographical narrative, acceptance of the role of “patient”, and negotiating inevitable changes in relationships with family, friends and colleagues. This characterisation of the work of self-management provided the overall framework for the content, tone and style of the intervention. As these tasks are intertwined and inter-dependent, we designed the content to address all three tasks in an integrated fashion.

Normalisation Process Theory is a mid-range sociological theory which predicts and explains whether and why certain innovations will be effectively implemented into routine health care and become “normalised”, that is, become so much part of routine practice that they disappear from view.[3] It posits that interventions which are easily understood and distinguished from other interventions (coherence), which relevant professionals can see the advantage of compared to current or alternative practice (cognitive participation), which fit well with existing professional relationships and workflows (collective action), and where users are provided with formal or informal feedback and evidence about the impact of the intervention (reflexive monitoring) are more likely to be implemented than those that do not. These constructs guided the development of the intervention by, for example, ensuring HeLP-Diabetes integrated well with existing workflows in general practice.

Effective self-management in diabetes often requires patients to change deep-rooted behaviours, particularly around diet and physical activity. Rather than adopt a single behavioural theory, we opted to design our behaviour change modules around specific

behaviour change techniques, selecting those with the best evidence base to support their use.[4]

Content

The overall content was broken down into 8 sections:

1. Understanding diabetes (information about the nature and causes of diabetes, and how it affects the body);
2. Staying healthy (motivational material about how to maintain optimal physical and emotional health and the importance of self-management; new behaviour change modules and previously validated programmes for diet, weight loss, physical activity, smoking cessation, moderating alcohol intake, and taking medicines);
3. Treating diabetes (information about medications used in diabetes, including information about indications, side effects and monitoring; importance of managing cardiovascular risk factors as well as glycaemic levels; importance of regular monitoring to prevent retinopathy, neuropathy and nephropathy; and types and roles of different health care professionals involved in caring for people with diabetes);
4. Living and working with diabetes (focus on managing social and work situations, such as shift work, parties, or holidays; impact on relationships, including sexual relationships; and possible impact on emotions and feelings of self-worth);
5. Managing my feelings (self-assessment tools for identifying low mood; cognitive behavioural therapy modules; mindfulness-based approaches);
6. My health record (opportunity to record appointments with health care professionals, results of tests or self-monitoring, with opportunities for graphical displays and feedback);
7. News and Research (updates about latest research or news about diabetes treatment, in depth articles about seminal research papers);
8. Forum and Help (moderated forum; videos of personal stories about diabetes (used with license from health talk online); additional resources, with local resources tailored according to Clinical Commissioning Group (CCG)).

Where possible we aimed to use existing modules with a proven evidence-base. Hence we used, with licence, the entire type 2 diabetes module from Health Talk Online, which contains videos of interviews with people with diabetes from a range of socio-economic and ethnic backgrounds, talking about how they were diagnosed, the impact diabetes has had on their lives and relationships, how they have adapted or come to terms with it, and treatments they have experiences (see www.healthtalk.org). Behaviour change programmes used with license included: POWeR (Positive Online Weight Reduction), a set of 12 modules shown to be effective for managing obesity in primary care[5]; Stop Advisor (smoking cessation)[6] and Down Your Drink (alcohol moderation).[7] New modules were developed for healthy eating, increasing physical activity and taking medication. These used the behaviour change techniques of action planning, goal setting, self-monitoring, reminders and feedback, along with information about the benefits of change. The section on “Managing my feelings” included the *Living Life to the Full* programme[8] – a set of 10 modules based on the principals of cognitive behavioural therapy, specifically adapted for people with type 2 diabetes, and used with license.

Procedures

HeLP-Diabetes was designed to be used as part of an overall package of care for people with diabetes. Low usage, or non-adherence to internet interventions is well-recognised problem, and our preparatory work with patients and health care professionals indicated that integrating the intervention into routine care was likely to improve uptake and adherence. Hence we made the programme available to registered users only, and encouraged health care professionals to register patients. Once registered, patients could use the programme as much (or as little) as they wanted. There was no prescribed level of use, as our proposed users included patients at all stages of their illness journey, from those newly diagnosed to those who had lived with diabetes for many years. As such, we anticipated that each user would have different needs and priorities, and the programme was designed to allow users to pick and choose sections that were most relevant and beneficial for them personally. There was a limited amount of tailoring. Additional resources and sources of help were tailored by the patient's CCG, but otherwise tailoring was limited to the behaviour change and health record sections where users entered their own goals or data.

Registration and facilitation.

During the trial, registration was performed by a practice nurse. During the registration procedures users were asked to select a password and username. After registration, the nurse was asked to demonstrate the intervention to the patient, and integrate use of the intervention into the patient's personal diabetes care plan by, for example, discussing what goals the patient would like to set for the coming period, and showing the patient how to use the intervention to set goals, monitor progress, and, if desired, arrange for automated reminders by SMS or email to be sent by the programme.

Registration was undertaken in the patient's practice. All subsequent use of the intervention was at any location convenient for the patient with internet access. For most people, we expected this to be at home, or at the home of a relative. However, all users were given information about local services (usually libraries) offering free access to an internet-connected computer.

Encouraging engagement

In addition to the registration and facilitation procedures described above, engagement to the programme was encouraged through regular prompts, provided by email or SMS. Emailed prompts were of two types: a) short (2 – 3 lines) emails with one message and a link to the relevant section of the programme. An example of this type of prompt was a seasonal reminder of the importance of flu vaccination for people with diabetes, with a link to the page of the programme explaining how and why flu vaccination is beneficial, and a reminder that flu vaccination is available free from their general practice. The second type of email prompt was a longer (1 page of A4) newsletter, which summarised recent research or news stories, explained what this meant for patients, and also contained a paragraph or two on specific health promotion – for example, the importance of regular foot care, how to fast safely during Ramadan, or tips for safe overseas travel. These stories and paragraphs also contained links to relevant sections of the programme. SMS prompts were very brief, with one clear message, and a link to the programme. We sent around 3 prompts per month – usually 2 short emails or SMS plus one longer newsletter. Users could opt out of receiving prompts.

Modifications during the trial

One of the key functions of the programme was to provide up-to-date, evidence-based information. Hence the site was regularly reviewed to ensure all content was up-to-date, evidence-base, and congruent with current NICE guidelines. In practice, this meant small updates each month, with a complete review when the NICE guidelines on management of diabetes were updated.[9]

Assessment of fidelity

Intervention use was assessed through bespoke software which recorded the date, time and pages viewed for each log in by each user. Practice nurses were trained in registration and facilitation procedures, but we were unable to monitor how well they adhered to them.

Availability of the intervention

The intervention is currently available for commissioning from a not-for-profit Community Interest Company, HeLP-Digital. The open access sections of the intervention can be viewed at http://www.helpdigitalcic.org.uk/products

References

1. Hoffmann TC, Glasziou PP, Boutron I, et al. Better reporting of interventions: template for intervention description and replication (TIDieR) checklist and guide. *BMJ (Clinical research ed)* 2014; **348**: g1687.
2. Corbin JM, Strauss A. Unending Work and Care. First ed. San Francisco: Jossey-Bass Inc; 1988.
3. May C, Finch T. Implementation, embedding, and integration: an outline of Normalization Process Theory. *Sociology* 2009; **43**(3): 535-54.
4. Abraham C, Michie S. A taxonomy of behavior change techniques used in interventions. *Health Psychol* 2008; **27**(3): 379-87.
5. Yardley L, Ware LJ, Smith ER, et al. Randomised controlled feasibility trial of a web-based weight management intervention with nurse support for obese patients in primary care. *The international journal of behavioral nutrition and physical activity* 2014; **11**: 67.
6. Brown J, Michie S, Geraghty AW, et al. Internet-based intervention for smoking cessation (StopAdvisor) in people with low and high socioeconomic status: a randomised controlled trial. *The Lancet Respiratory medicine* 2014; **2**(12): 997-1006.
7. Wallace P, Murray E, McCambridge J, et al. On-line Randomized Controlled Trial of an Internet Based Psychologically Enhanced Intervention for People with Hazardous Alcohol Consumption. *PLoSONE* 2011; **6**(3): e14740.
8. Williams C, Association CMH. Living life to the full: Media innovations; 2008.
9. NICE. Type 2 diabetes in adults: Management. NG28. London, 2015.
