# Supplementary material for: Web-based self-management support for people with type 2 diabetes (HeLP-Diabetes): randomised controlled trial in English primary care
Source: BMJ Open. 2017 Sep 27;7(9):e016009. doi: 10.1136/bmjopen-2017-016009 (PMC5623569; doi:10.1136/bmjopen-2017-016009)
Supplement: Supplementary file 2 [file bmjopen-2017-016009supp002.pdf]

Appendix 2: Additional statistical methods and results

Statistical Methods for Multiple Imputation

Multiple Imputation using chained equation was used as the primary method to account for missing data (in both baseline and follow-up data).[1] A set of imputation models were specified, one for each variable with missing data. Each variable was then regressed on all other variables, including completely recorded baseline and follow-up variables and stratified by randomised group. Imputations were performed using predictive mean matching using the five nearest neighbours to the prediction as a set to draw from. The full list of variables considered in the MICE approach is shown in Supplementary Table 1, together with the number of missing values for each variable and time period.

Since only measurements within a 10-14 month window period were used within the main analyses of HbA1c and PAID, the following imputation procedure was implemented for these two co-primary outcomes. Twelve month measurements were subdivided into those that were measured within 10-14 months (the primary outcome variable) and those that were measured outside 10-14 months (a variable used for imputing only). For HbA1c, two additional variables were created for use within the imputation model; 1) the time in days from randomisation that the "12-month" HbA1c measurement was actually taken for values inside of the window period (and set to 365 for measurements taken outside the window period), 2) the time in days from randomisation that the "12-month" HbA1c measurement was actually taken for values outside the window period (and set to missing for measurements taken within the window period). The first of these variables gives the desired time for imputing HbA1c measurements when they are missing, whilst the second gives information on how far outside of the window the actual measurements were taken. Corresponding variables were created for the "12-month" PAID measurement. Finally, two additional variables were created defining the time in days at which HbA1c and PAID were measured at "3-months". All variables were included within the chained equations and imputed where necessary.

40 imputed datasets were created, the analysis models were fitted to each imputed dataset separately, and the estimates were pooled using Rubin's rules.

Statistical Methods for causal analyses

The causal analysis proposed attempts to address how the effectiveness of the intervention is mediated through the frequency of website usage. In particular, it is important to understand whether prolonged usage of the website modifies the efficacy of the intervention. Since website usage is measured post-randomisation a naïve analysis of correlating usage with outcomes in the intervention group may give biased and misleading results, since there may be unmeasured confounders also correlated with the outcomes that distinguish the motivated users who regularly log-in from the less motivated ones. Causal analyses using instrumental variables (IV) were therefore used to determine the effect of website usage on outcomes. This approach preserves randomisation (i.e. provides a comparison independent of observed and unobserved confounders).

"Usage" is defined as the proportion of follow-up (rescaled as no. days in a year) that the HeLP-Diabetes website is accessed. It was assumed that the efficacy of the intervention is zero for individuals who never log-in (the exclusion restriction assumption). Website usage in the control group was ignored in the model as it was assumed that the control website was unlikely to be effective. (NB. usage statistics were collected for the comparator website but were not used in these analyses).

This main underlying assumption of the causal analysis is that the effect of randomisation to the HeLP-Diabetes intervention on 12-month outcomes occurs only through use of the website (Supplementary Figure 1; Z is randomised intervention, WU is website-usage and Y is 12-month outcome, e.g. HbA1c or PAID). This relies of the “exclusion restriction” assumption that the HeLP Diabetes intervention has no effect when usage is zero (i.e. for individuals who never log-in). Hence randomisation is assumed to be an instrumental variable.

Supplementary Figures 2 and 3 show the estimated causal effects of HeLP Diabetes on HbA1c and PAID at 12-months, respectively, by level of website usage (the “exclusion restriction” assumption leads to zero efficacy at zero usage). To determine the casual estimate of efficacy for a “high-usage” group, the individual predicted efficacy was calculated for each individual greater than or equal to the median usage of 4 days (shown as the red boxes in the histograms in Supplementary Figures 2 and 3) and the average efficacy for this group was obtained. The mean usage in the “high-usage” group was 18 days.

Supplementary Figure 1. Causal model assuming no direct effect of intervention (complete mediation through website usage).

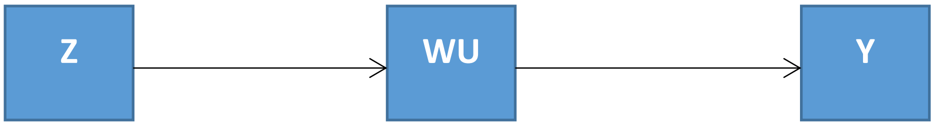

In this causal model, randomisation (Z) is the instrumental variable, which acts on the outcome (Y) exclusively through website usage (WU).

Supplementary Figure 2: Efficacy of HeLP-Diabetes on HbA1c at 12-months based on number of days usage, superimposed by the distribution of usage observed in the HeLP-Diabetes group. The bars shown in red highlight the "high-usage" group that have usage greater than or equal to the median of 4 days.

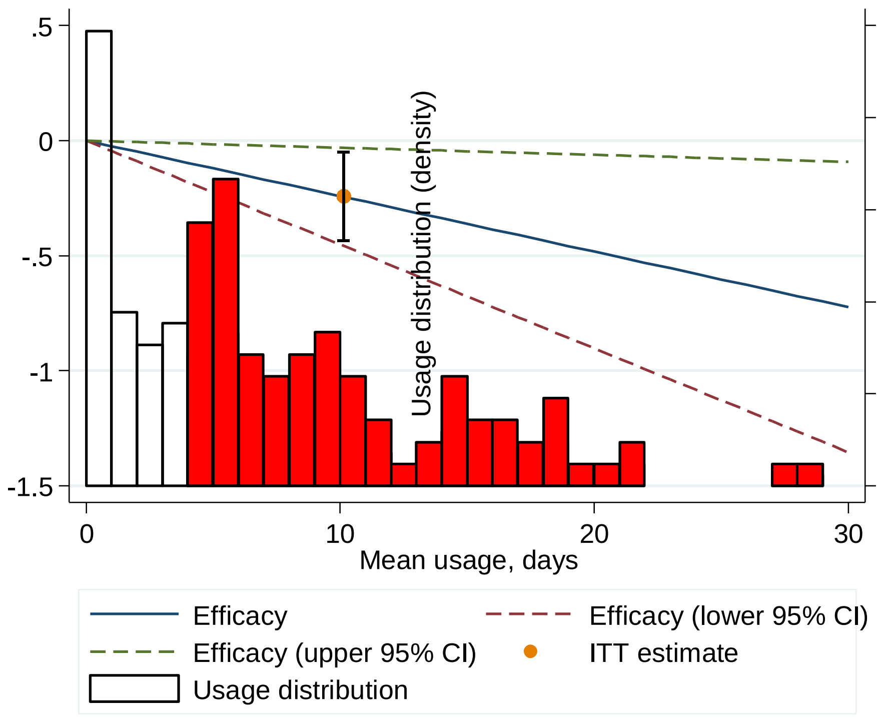

Supplementary Figure 3. Efficacy of HeLP-Diabetes on PAID score at 12-months based on number of days usage, superimposed by the distribution of usage observed in the HeLP-Diabetes group. The bars shown in red highlight the "high-usage" group that have usage greater than or equal to the median of 4 days.

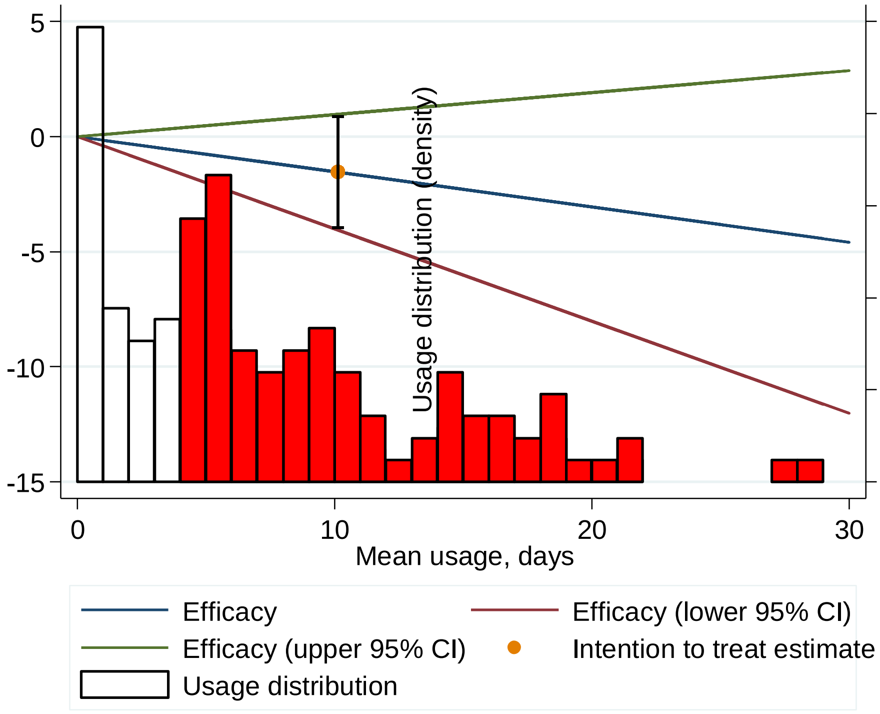

Supplementary Figure 4. Means and 95% CIs of HbA1c over time by randomised group and completers vs. non-completers, after multiple imputation of non-completers follow-up

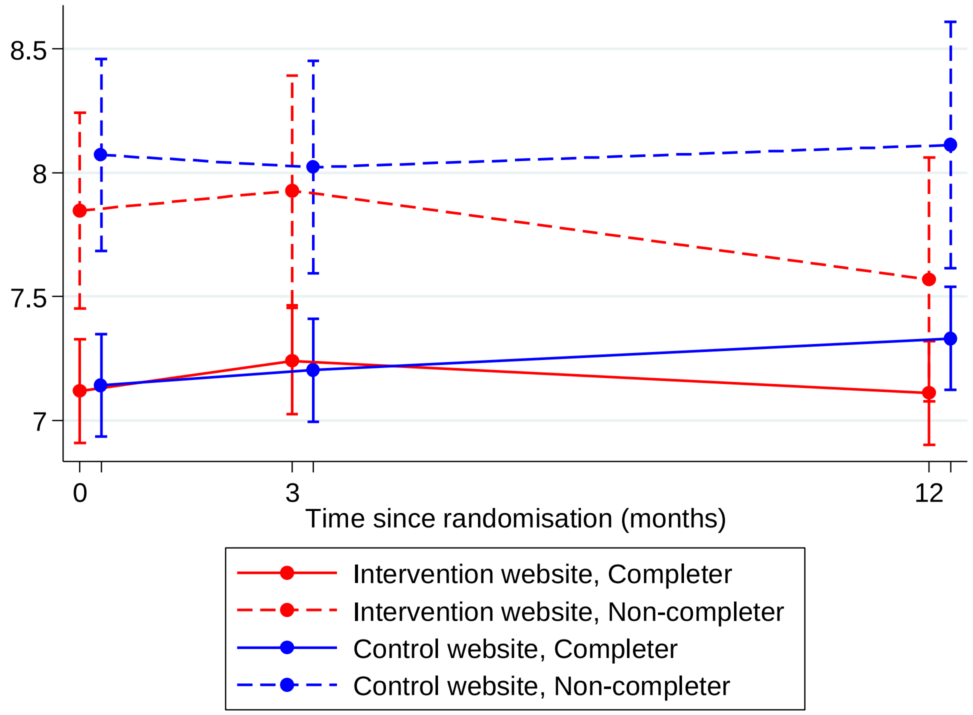

Supplementary Table 1. List of variables imputed and amount of missing data at baseline, 3 months and 12 months.

Variable	Number of missing values		
	Baseline	3 months	12 months
HbA1c	5	64	83 10-14months †
			347 Outside 10-14months †
PAID	0	54	53 10-14months †
			358 Outside 10-14months †
Systolic Blood Pressure	0	57	68
Diastolic Blood Pressure	0	57	68
Body Mass Index	2	58	69
Total cholesterol	2	68	75
HDL cholesterol	12	73	76
Completion of 9 essential processes	69	-	69
HADS	0	74	107
DMSES	0	73	109
DTSQ	0	77	109
Age	0	-	-
Sex	0	-	-
Duration of diabetes	4	-	-
History of cardiovascular disease	0	-	-
Attending any other self-management class	0	-	-
Smoking status	0	-	-
No of visits to the website	-	-	0
No of webpages visited	-	-	105
Average time per visit	-	-	105

* Partial mean matching performed using 5 nearest neighbours

† 12-month measurements were subdivided into those that were measured within 10-14 months (primary outcome variable) and those that were measured outside 10-14 months (variable used for imputing only).

Supplementary Table 2: Descriptive statistics of 3-month variables by randomised group. Mean (SD) unless otherwise specified.

	HeLP-Diabetes	Control	p-value*	N missing
**Clinical Measures**				
Systolic blood pressure (mmHg)	133 (16)	134 (17)	0.350	57
Diastolic blood pressure (mmHg)	76 (10)	75 (9)	0.699	57
Total cholesterol (mmol/l)	4.17 (0.97)	4.16 (1.04)	0.900	68
HDL-C (mmol/l)	1.29 (0.37)	1.24 (0.37)	0.356	73
Total cholesterol /HDL-C ratio	3.48 (1.15)	3.52 (1.09)	0.570	74
HbA1c (%)	7.34 (1.48)	7.34 (1.24)	0.323	64
Body mass index (Kg/m^2)	30.0 (5.4)	30.0 (5.6)	0.545	58
**Questionnaires / scores**				
PAID (0-100)	15.2 (15.2)	16.7 (17.1)	0.492	54
HADS (0-42)	8.84 (6.54)	8.86 (7.45)	0.603	74
Anxiety scale (0-21)	4.75 (3.88)	5.07 (4.24)	0.639	74
Depression scale (0-21)	4.10 (3.30)	3.80 (3.59)	0.205	74
DMSES (0-150)	103.9 (32.2)	104.9 (32.7)	0.795	73
DTSQ (0-48)	32.8 (6.9)	32.9 (6.3)	0.715	77

* Wilcoxon rank-sum test

Supplementary Table 3. Effect of HeLP-Diabetes vs. Control on 12-month primary outcomes, HbA1c and PAID. Results from primary analyses and six sensitivity analyses

	**HbA1c, %**	
	Number individuals	mean difference (95% CI)<br>HeLP-Diabetes - Control
Primary Analysis<br>Multiple Imputation of both baseline and outcomes	374	-0.242 (-0.435, -0.049); p=0.014
Sensitivity Analysis 1:<br>(multiple imputation of baseline covariates only)	291	-0.214 (-0.390, -0.038); p=0.017
Sensitivity Analysis 2:<br>(complete cases) ƚ	284	-0.207 (-0.385, -0.028); p=0.023
Sensitivity Analysis 3:<br>(complete cases) ǂ	186	-0.220 (-0.416, -0.025); p=0.027
Sensitivity Analysis 4:<br>(non-contaminated cases) ¥	370	-0.225 (-0.418, -0.032); p=0.023
Sensitivity Analysis 5:<br>Linear model excluding centre*	374	-0.242 (-0.438, -0.047); p=0.015
Sensitivity Analysis 6:<br>Unadjusted model (complete-cases)	291	-0.220 (-0.483, 0.043); p=0.100
	**PAID**	
	Number individuals	mean difference (95% CI)<br>HeLP-Diabetes - Control
Primary Analysis<br>Multiple Imputation	374	-1.54 (-3.94, 0.87); p=0.209
Sensitivity Analysis 1:<br>(multiple imputation of baseline covariates only)	321	-1.31 (-3.65, 1.03); p=0.274
Sensitivity Analysis 2:<br>(complete cases) ƚ	317	-1.29 (-3.66, 1.08); p=0.285
Sensitivity Analysis 3:<br>(complete cases) ǂ	65	-2.22 (-6.35, 1.91); p=0.292
Sensitivity Analysis 4:<br>(non-contaminated cases) ¥	370	-1.57 (-3.99, 0.85); p=0.204
Sensitivity Analysis 5:<br>Linear model excluding centre**	374	-1.54 (-3.98, 0.90); p=0.215
Sensitivity Analysis 6:<br>Unadjusted model (complete-cases)	321	-2.38 (-6.02, 1.26); p=0.199

ƚ excluding outcomes outside 10-14 months post-randomisation and those missing baseline covariates

ǂ excluding outcomes outside 11-13 months post-randomisation and those missing baseline covariates

¥ Excluding patients who were suspected to have been exposed to the alternative intervention

* likelihood ratio test for including centre in the model as a fixed effect; p=0.617 (HbA1c)

** likelihood ratio test for including centre in the model as a fixed effect; p=0.357 (PAID)

Supplementary Table 4. Effect of subgroups on the effectiveness of HeLP-Diabetes in reducing HbA1c and PAID.

Subgroup	**HbA1c, %**			
	Number individuals	mean difference (95% CI) HeLP-Diabetes - Control		Interaction p-value*
		Complete Case ƚ	Multiple Imputation	
Baseline HbA1c				0.458
<7.5%	254/369	-0.15 (-0.41, 0.11)	-0.14 (-0.39, 0.12)	
≥7.5%	115/369	-0.51 (-0.94, -0.07)	-0.51 (-0.93, -0.09)	
Duration of diabetes				0.425
<6.9 yrs	185/370	-0.34 (-0.59, -0.08)	-0.30 (-0.57, -0.03)	
≥6.9 yrs	185/370	-0.07 (-0.32, 0.18)	-0.18 (-0.45, 0.09)	
	**PAID**			
Baseline PAID				0.066
<12	181/374	-0.3 (-4.6, 4.1)	-0.1 (-4.2, 4.1)	
≥12	193/374	-3.6 (-7.8, 0.7)	-3.9 (-8.2, 0.4)	
Duration of diabetes				0.004
<6.9 yrs	185/370	-2.9 (-6.3, 0.4)	-3.6 (-7.0, -0.1)	
≥6.9 yrs	185/370	0.3 (-3.0, 3.7)	0.4 (-3.1, 3.9)	

ƚ excluding outcomes outside 10-14 months post-randomisation and those missing baseline covariates

* p-value for interaction based on a test of linear trend with the continuous variable.

Reference List

1. White IR, Royston P, Wood AM. Multiple imputation using chained equations: Issues and guidance for practice. *Statistics in medicine* 2011; **30**(4): 377-99.
