# Supplementary material for: Web-based self-management support for people with type 2 diabetes (HeLP-Diabetes): randomised controlled trial in English primary care
Source: BMJ Open. 2017 Sep 27;7(9):e016009. doi: 10.1136/bmjopen-2017-016009 (PMC5623569; doi:10.1136/bmjopen-2017-016009)
Supplement: Author's manuscript [file bmjopen-2017-016009.draft_revisions.pdf]

BMJ Open is committed to open peer review. As part of this commitment we make the peer review history of every article we publish publicly available.

When an article is published we post the peer reviewers' comments and the authors' responses online. We also post the versions of the paper that were used during peer review. These are the versions that the peer review comments apply to.

The versions of the paper that follow are the versions that were submitted during the peer review process. They are not the versions of record or the final published versions. They should not be cited or distributed as the published version of this manuscript.

BMJ Open is an open access journal and the full, final, typeset and author-corrected version of record of the manuscript is available on our site with no access controls, subscription charges or pay-per-view fees (http://bmjopen.bmj.com).

If you have any questions on BMJ Open's open peer review process please email info.bmjopen@bmj.com

BMJ Open

Web-based self-management support for people with type 2 diabetes (HeLP-Diabetes): randomised controlled trial in English primary care.

Journal:	*BMJ Open*
Manuscript ID	bmjopen-2017-016009
Article Type:	Research
Date Submitted by the Author:	17-Jan-2017
Complete List of Authors:	Murray, Elizabeth; University College London, Research Department of Primary Care and Population Health<br>Sweeting, Michael; MRC Biostatistics Unit, Cambridge,<br>Dack, Charlotte; University of Bath, Department of Psychology<br>Pal, Kingshuk; University College London, Department of Primary Care & Population Health<br>Modrow, Kerstin; University College London, Research Department of Primary Care and Population Health<br>Hudda, Mohammed; MRC Biostatistics Unit, Cambridge,<br>Li, Jinshuo; University of York, Department of Health Sciences<br>Ross, Jamie; University College London, Primary Care & Population Health<br>Alkhaldi, Ghadah; University College London, Research Department of Primary Care and Population Health<br>Barnard, Maria; Whittington Health NHS Trust<br>Farmer, Andrew; University of Oxford, Department of Primary Care Health Sciences<br>Michie, Susan; University College London, Centre for Outcomes Research and Effectivenes<br>Yardley, Lucy; University of Southampton, Academic Unit of Psychology<br>May, Carl; University of Southampton, Faculty of Health Sciences<br>Parrott, Steve; University of York, Department of Health Sciences<br>Stevenson, Fiona; UCL, Primary care and population sciences<br>Knox, Malcolm; University College London, Research Department of Primary Care and Population Health<br>Patterson, David; Whittington Health NHS Trust
**Primary Subject Heading**:	Diabetes and endocrinology
Secondary Subject Heading:	General practice / Family practice, Health informatics, Health services research, Patient-centred medicine
Keywords:	internet, self care, Diabetes mellitus, type 2, PRIMARY CARE

SCHOLARONE™
Manuscripts

For peer review only

Title: Web-based self-management support for people with type 2 diabetes (HeLP-Diabetes): randomised controlled trial in English primary care.

Authors:

Elizabeth Murray[1], Professor of eHealth and Primary Care
Michael Sweeting[2], Senior Research Associate
Charlotte Dack[3], Lecturer,
Kingshuk Pal[1], Clinical Research Associate
Kerstin Modrow[1], Data Manager
Mohammed Hudda[4], Research Fellow in Medical Statistics
Jinshuo Li[5], Research Fellow
Jamie Ross[1], Research Associate
Ghadah Alkhaldi[1] PhD student
Maria Barnard[6], Consultant in Diabetes and Endocrinology,
Andrew Farmer[7], Professor of General Practice
Susan Michie[8], Professor of Health Psychology
Lucy Yardley[9], Professor of Health Psychology
Carl May[10], Professor of Health Care Innovation
Steve Parrott[5] Reader in Health Economics,
Fiona Stevenson[1] Reader in Medical Sociology
Malcolm Knox[1] Patient Representative
David Patterson[6], Consultant Cardiologist

[1]Research Department of Primary Care and Population Health, University College London, London NW3 2PF, UK
[2]Cardiovascular Epidemiology Unit, Department of Public Health and Primary Care, University of Cambridge, Worts Causeway, Cambridge, CB2 0SR
[3]Department of Psychology, University of Bath, Claverton Down, Bath, BA2 7AY
[4]Population Health Research Institute, St George's, University of London, Cranmer Terrace, London SW17 0RE
[5]Department of Health Sciences, University of York. Seebohm Rowntree Building, University of York, Heslington, York, YO10 5DD, UK
[6]Whittington Health, Magdala Avenue, London N19 5NF
[7]Nuffield Department of Primary Care Health Sciences, University of Oxford, Radcliffe Observatory Quarter, Woodstock Road, Oxford. OX2 6GG
[8]Centre for Behaviour Change, Research Department of Clinical, Educational and Health Psychology, University College London, 1-19 Torrington Place, London, WC1E 7HB
[9]Department of Psychology, Building 44 Highfield Campus, University of Southampton SO17 1BJ
[10]Health Sciences, University of Southampton, University Road, Southampton SO17 1BJ

Author for correspondence:
Professor Elizabeth Murray, Research Department of Primary Care and Population Health, University College London, London NW3 2PF, UK. Elizabeth.murray@ucl.ac.uk

For peer review only - http://bmjopen.bmj.com/site/about/guidelines.xhtml

Abstract

Objective: To determine the effectiveness of a web-based self-management programme for people with type 2 diabetes in improving glycaemic control and reducing diabetes-related distress.

Methods

Design: Individually randomised two-arm controlled trial

Setting: 20 General Practices in England

Participants: Adults aged 18 or over with a diagnosis of type 2 diabetes registered with participating general practices

Intervention and comparator: Usual care plus ***either*** HeLP-Diabetes, an interactive, theoretically informed, web-based self-management programme ***or*** a simple, text-based website containing basic information only.

Outcomes and data collection: Joint primary outcomes were glycated haemoglobin (HbA1c) and diabetes-related distress, measured by the Problem Areas in Diabetes (PAID) scale, collected at 3 and 12 months after randomisation, with 12 months the primary outcome point. Research nurses, blind to allocation collected clinical data; participants completed self-report questionnaires online.

Analysis: The analysis compared groups as randomised (intention to treat) using a linear mixed effects model, adjusted for baseline data with multiple imputation of missing values.

Results

Of the 374 participants randomised between September 2013 and December 2014, 185 were allocated to the intervention and 189 to the control. Final (12 month) follow up data for HbA1c were available for 318 (85%) and for PAID 337 (90%) of participants. Of these, 291 (78%) and 321 (86%) responses were recorded within the pre-defined "window" of 10-14 months. Participants in the intervention group had lower HbA1c than those in the control (mean difference -0.24%; 95% Confidence Interval -0.44 to -0.049; p=0.014). There was no significant overall difference between groups in the mean PAID score (p=0.21), but pre-specified subgroup analysis of participants who had had diabetes for less than 7 years showed a beneficial impact of the intervention in this group (p = 0.004). There were no reported harms.

Conclusions

Access to HeLP-Diabetes improved glycaemic control over 12 months.

Registration

Trial registration ISRCTN02123133.

Word count of abstract = 299.

For peer review only

Strengths and Limitations of this study

- Self-management improves outcomes and health status in people with type 2 diabetes; but uptake is poor, partly because some people find the current model of group-based structured education inconvenient or unappealing.
- Web-based self-management support may help improve uptake.
- The trial recruited to target and achieved reasonable follow-up; however trial participants may not be representative of the total population of people with type 2 diabetes in primary care.

For peer review only

For peer review only - http://bmjopen.bmj.com/site/about/guidelines.xhtml

Introduction.

There is a global epidemic of type 2 diabetes mellitus (T2DM). An estimated 422 million adults, or 10% of the global population, were living with diabetes in 2014 of whom around 90% had type 2 diabetes.[1] Poorly controlled diabetes is associated with premature mortality, and a high risk of complications, including cardiovascular disease, nephropathy and retinopathy. The risk of complications can be reduced by good control of glycaemia and cardiovascular risk factors. [2 3] Interventions which improve self-management skills for patients with diabetes can improve health outcomes and reduce health care costs[4] and international guidelines support training patients in self-management. [3 5] However, it is not clear how best to support patients in developing such skills, and uptake of diabetes self-management education remains low. In England, despite over 90% of eligible patients being referred, [6] only 5.3% attended self-management training in 2014 – 15. [7]

Poor uptake may be related to the dominant model of structured education, which is group-based sessions, lasting a half or whole day, or spread over regular sessions over several weeks. [8] Many patients, such as those who work, those with caring commitments, or those who are uncomfortable in groups, may find it difficult to attend.[9 10]

Web-based support for self-management could address some of these barriers, particularly in high income countries, where levels of web-access are high. In the UK over 80% of households had Internet access in 2015, and internet access amongst older people continues to grow steadily. [11 12] Potential advantages include convenience, anonymity, regular updates, and the potential to use video and graphics to present complex information in a format accessible to those with low literacy.[13] Although systematic reviews have confirmed that computer-based interventions can improve health outcomes in diabetes [14], not all such interventions have a beneficial impact, with meta-analyses showing substantial heterogeneity related to widely differing interventions, including in the use of theory to develop the intervention, [15] outcomes, [14 16] and the duration of follow-up, with most trials having relatively short follow-up (less than 12 months). [14] This is the first UK-based trial of a comprehensive, web-based self-management support programme for people with type 2 diabetes.

This trial assessed the effects of a web-based self-management programme, called Healthy Living for People with Diabetes (HeLP-Diabetes), on glycated haemoglobin (HbA1c) and diabetes-related distress over 12 months.

Methods.

Trial Design and participants: multi-centre, two-arm individually randomised controlled trial in 20 General Practices in England with a mix of urban, suburban and rural practices. Practices were required to have two nurses – one to facilitate access to the intervention, and one to collect data. Participants were adults, aged 18 or over, with T2DM, registered with participating general practices. Patients were excluded if they were unable to provide informed consent; unable to use a computer due to severe mental or physical impairment; had insufficient spoken or written English to use the intervention (operationalised as unable to consult without an interpreter); were terminally ill with less than 12 months life expectancy; or were currently participating in a trial of an alternative self-management programme. Participants were not required to have home Internet access or prior experience of using the Internet to participate. Participants with previous or current experience of self-management education were eligible to participate. Recruitment took place between September 2013 and December 2014. The trial protocol was submitted for publication in June 2014.[17] There were no changes to the methods after the protocol was agreed and the start of the trial. Ethical

For peer review only - http://bmjopen.bmj.com/site/about/guidelines.xhtml

approval was obtained from Camden and Islington National Research Ethics Service (NRES) committee, reference 12/LO/1571.

Patient involvement

Patients were involved in all stages of the study, including contributing to the original application for funding as co-investigators; substantive and ongoing contribution to intervention development; contributing to the trial design, including the decision to have two co-primary outcomes; active membership of the Trial Steering Committee and Trial Management Group; and contributing to the writing of this paper. This last role is recognised through co-authorship.

Randomisation and blinding

Randomisation marked the point of study entry. It was performed centrally (independently of the trial team), after written informed consent was obtained and all baseline data were completed, using a web-based randomisation system, at the level of the individual participant. Randomisation was conducted in a 1:1 ratio using random permuted blocks of sizes 2, 4 and 6, stratified by recruitment centre. Participants were informed the trial compared two forms of web-based support, and were blinded as to which was the intervention and which the comparator. Nurses who offered facilitation for the intervention could not be blinded, but were asked not to discuss details of allocation with the nurses who gathered follow-up data. The research team obtaining and analysing data from participants were blind to allocation.

Intervention

The intervention consisted of facilitated access to HeLP-Diabetes. Facilitation consisted of an introductory training session with the practice nurse. In this appointment patients were were shown how to log on, set a user name and password, and introduced to the content of the website. HeLP-Diabetes was a theoretically informed web-based programme whose overall goals were to improve health outcomes and reduce diabetes-related distress.[18] Overall content was guided by the Corbin and Strauss model of managing a long term condition which posits that patients must undertake medical, emotional and role management.[19] It was developed using participatory design principles, with substantial input from users, defined as patients with T2DM and health professionals caring for such patients. All content was evidence-based, drawing on evidence on management of diabetes, promoting behaviour change and emotional wellbeing, and maximising usability and engagement. Content was designed to be accessible to people with a wide range of literacy and health literacy skills, with all essential content provided in both video and text. There were information sections on diabetes, how diabetes is treated, possible complications of diabetes, possible impacts of diabetes on relationships at home and at work, dealing with unusual situations like parties, holidays, travelling or shift work, and what lifestyle modifications will improve health. There were sections addressing skills and behaviour change, including behaviour change modules on eating healthily, losing weight, being more physically active, smoking cessation, moderating alcohol consumption, managing medicines, glycaemic control and blood pressure control. Users could set the programme to send themselves reminder text messages or emails, and could specify the content and frequency of such reminders. The third strand of components focused on emotional well-being with self-help tools based on cognitive behavioural therapy and mindfulness. There were multiple personal stories (used with license from health talk online), and a moderated forum. Participants were free to use the programme as much or as little as they chose. Engagement with the programme was promoted through regular newsletters, emails and SMS containing updates on latest diabetes-related research or practice, seasonally-relevant advice (e.g. fasting during Ramadan, benefits of 'flu vaccinations), and links to specific relevant parts of the programme. Two or three prompts were sent each month, although users could opt out of receiving them. Further details are provided in Appendix 1.

For peer review only - http://bmjopen.bmj.com/site/about/guidelines.xhtml

Comparator

From an NHS perspective, the important research question was whether the proposed intervention could improve health outcomes when compared to current practice. However, to improve acceptability to participants and to maintain blinding, all participants had access to a website. Participants in the control arm were given access to a simple information website, based on the information available on the website of the main UK diabetes charity (Diabetes UK) or National Health Service patient information website (NHS Choices). They received the same initial facilitation meeting as participants in the intervention group, in which they were shown how to log on, set a user name and password, and how to use the website.

Outcomes and outcome measures

Primary outcomes

The outcomes reflected the dual goals of improving health outcomes and reducing diabetes-related distress. The two joint primary outcomes were glycated haemoglobin (HbA1c) and diabetes-related distress, measured by the Problem Areas in Diabetes (PAID) scale, both at 12 months post-randomisation. PAID has 20 items focusing on areas that cause difficulty for people living with diabetes, including social situations, food, friends and family, diabetes treatment, relationships with health care professionals and social support.[20] PAID scores range from 0 – 100, with higher scores indicating more distress. A score of 40 or more indicates significant distress, and around 40% of patients with diabetes experience significant distress.[21]

Secondary outcomes

Clinical secondary outcomes included systolic and diastolic blood pressure; body mass index; total cholesterol and HDL (not fasting); and completion of the "9 essential processes" for effective management of diabetes, mandated by NHS England (= weight, BP, smoking status, measurement of serum creatinine, cholesterol and HbA1c, urinary albumin and assessment of eyes and feet) within the previous 12 months.[3] Patient-reported outcomes included depression and anxiety, measured using the Hospital Anxiety and Depression Scale (HADS);[22] diabetes-related self-efficacy measured using the Diabetes Management Self-Efficacy Scale (DMSES);[23] and satisfaction with treatment, measured using the Diabetes Satisfaction with Treatment Questionnaire status and change version (DTSQs & DTSQc).[24]

Data collection

Data were collected at baseline, 3 and 12 months, with 12 months the primary endpoint. Patient-reported data were collected using online questionnaires emailed to participants. Clinical outcomes were collected by nurses in participating practices. Participants were asked to complete their online questionnaires before visiting the nurse for clinical measurements and blood tests. Blood samples were analysed at the local NHS laboratory used by participating practices for routine clinical analyses. Data on completion of the "9 essential processes" were collected from the GP record for the 12 months prior to randomisation and the 12 months after randomisation at the 12 month follow-up point to avoid triggering behaviour change amongst the study nurses. Use of the intervention was recorded automatically using bespoke software that recorded the date, and time of each page visited. A new log-in to the intervention was defined as any page that was accessed 30 minutes or more after the last accessed page.

Sample size Calculation

For peer review only - http://bmjopen.bmj.com/site/about/guidelines.xhtml

For peer review only

Our original sample size calculation was that randomising 350 participants with 85% follow-up would provide 90% power at the 5% level of significance to detect a 0.25% difference in HbA1c and a 4.0 point difference in PAID score (both minimal clinically important differences) at 12 months post-randomisation between the randomised groups.[25 26] Since HbA1c and PAID were joint primary outcomes measuring different aspects of T2DM, both were tested at a 5% significance level.

Analysis

The analysis followed a pre-specified analysis plan, based on comparing the groups as randomised (intention-to-treat). The analysis plan was approved by the Trial Steering Committee before unblinding and uploaded to the trial website, https://www.ucl.ac.uk/pcph/research-groups-themes/ehealth/projects/projects/helpdiabetesrct. Only HbA1c and PAID measured within a 10-14 month window period following randomisation was used in the primary analysis with missing 12-month outcomes multiply imputed using baseline and other outcome data (e.g. 3m data and final follow-up data collected outside the 10-14 month window). Further information on the imputation method is given in Appendix 2.

A linear mixed effects model with random centre effects was used to analyse each of the primary outcomes separately, adjusting for the baseline level of the outcome, age, gender, previous participation in other self-management programmes, pre-existing cardiovascular disease and time since diagnosis of diabetes. Secondary outcome measures were analysed similarly using generalised linear mixed models. Pre-specified sub-group analysis for the co-primary outcomes was undertaken by baseline glycaemic control (HbA1c outcome only), baseline PAID (PAID outcome only), and duration of diabetes, treating all potential effect modifiers as continuous. The interaction between randomised group and each effect modifier was included in the model separately and assessed using a Wald test.

Use of the intervention was investigated as a mediator for efficacy, using instrumental variable methods, with randomisation as the instrument.[27 28]

Potential contamination was monitored by recording participants with similar family names and identifying those with the same addresses. Where this occurred, it was dealt with in the analysis by reporting the extent and undertaking a sensitivity analysis excluding these individuals.

A number of other sensitivity analyses were performed to assess the robustness of the primary analyses: 1) performing two complete case analyses disregarding outcomes measured outside 10-14 months and 11-13 months post-randomisation; 2) repeating the analysis using multiple imputation of baseline covariates only; and 3) fitting linear models excluding centre random-effects.

The TSC took on the role of the data monitoring committee. Trial registration ISRCTN02123133.

Results

Recruitment took place between September 2013 and December 2014. An initial 421 patients consented to participate, but of these 47 did not fully complete their baseline questionnaires and were therefore not randomised and did not enter the study. A total of 374 participants were randomised, of whom 86% (n = 321) provided data on PAID, and 78% (n = 291) had HbA1c measured within 10 to 14 months of randomisation. Additional final outcome data, obtained outside the 10 – 14 month pre-defined window, were available for a further 27 participants for HbA1c and 16 participants for PAID (Figure 1). Data obtained

For peer review only - http://bmjopen.bmj.com/site/about/guidelines.xhtml

outside the 10 – 14 month window were not used directly in the primary analysis, but were entered into the imputation model.

Baseline characteristics

Baseline demographic and clinical characteristics are shown in Table 1. The mean age was nearly 65 years, over two-thirds (n = 258, 69%) were male, and most were White British (n = 300, 80%). Nearly all (n=370, 99%) had a computer with access to the internet at home and just over half (n = 210, 56%) rated themselves as experienced computer users. Around one-third (n = 134; 36%) had been diagnosed for less than 5 years, with a further third (n = 115, 31%) having been diagnosed between 5 and 9 years ago. Overall, this was a population with well-controlled diabetes at baseline (mean HbA1c was 7.3% (56 mmol / mol)) and low levels of distress (mean PAID = 19).

Primary outcomes

At twelve months the primary analysis showed a significant difference in change in HbA1c between the randomised groups with participants in the HeLP-Diabetes group having a lower HbA1c than those in the control group (mean difference = -0.24%; 95% confidence intervals -0.44 to -0.05, p=0.014) (Table 2, Figure 2). There was no difference in change in PAID scores between the groups at 12-months (mean difference -1.5; 95% CI -3.9, 0.9, p=0.209), though both groups showed a decrease in PAID over the follow-up of the trial (Table 2, Figure 3).

Secondary outcomes

There was no difference in secondary outcomes at 12 months, with the exception of systolic blood pressure, which decreased more in the intervention group than in the control group (p = 0.010) (Table 2). There were no significant differences between groups on any of the outcome measures amongst individuals who completed three month outcomes (Supplementary Table 2). No adverse effects or events were recorded during follow-up.

Usage data

The mean number of log-ins was significantly higher in the intervention group than the control group (18.7 vs. 4.8, p= 0.0001), as was the mean number of pages visited per log-in (10.5 vs. 7.7, p <0.0001) and the mean number of days in which the website was accessed (10.1 vs. 3.3, p<0.0001) (Table 3). The causal analyses estimated that for a "high-usage" population (those with usage ≥ the median of 4 days) the HeLP-Diabetes intervention could on average reduce HbA1c by -0.44% (95% CI -0.81, -0.06) and PAID by -2.8 (95% CI -7.2, 1.7) over 12 months (Supplementary Figures 2 and 3). The mean usage in the "high-usage" group was 18 days. It should benoted that the usage data presented do not include the initial facilaition visit. There was a technical error in the software which led to usage data not being collected before 1 January 2014. At this point 16 participants had been randomised (7 to intervention, 9 to control). For these 16 participants, the usage data is not based on a full year, but for all other participants, data are summarised for the 12 months post-randomisation.

Sensitivity analyses

The findings from the sensitivity analyses, including a complete-case analysis, were similar to the main analysis (Supplementary Table 3). Participants who were missing 12-month HbA1c had significantly higher mean baseline HbA1c measures (7.9% vs. 7.1%, p<0.001) leading to higher imputed HbA1c at 12-months in the non-completers and a greater mean difference between the randomised groups than from complete case analyses (Supplementary Figure 4, Supplementary Table 3).

Subgroup analyses

Pre-specified subgroup analyses showed that there was no evidence of baseline measures of HbA1c or PAID being effect modifiers for the mean difference between the groups. There

For peer review only - http://bmjopen.bmj.com/site/about/guidelines.xhtml

was strong statistical evidence (interaction p=0.004) to suggest that the duration of diabetes acted as an effect modifier, with those who had been diagnosed more recently (<7 years) showing more of a reduction in PAID than those who had been diagnosed for longer periods of time. Duration of diabetes had no effect on change in HbA1c (Supplementary Table 4).

Harms

There were no reported harms in either group.

Discussion

In this first UK-based trial of a web-based self-management programme for people with T2DM, participants randomised to HeLP-Diabetes demonstrated improved glycaemic control at 12 months compared to those randomised to a simple information website. This improvement is both clinically and statistically meaningful, appears robust across all pre-specified sensitivity analyses, and was not dependent on duration of diabetes, baseline glycaemic levels or level of diabetes-related distress. Each 1% reduction in HbA1c is associated with a risk reduction of 21% for deaths related to diabetes and a 37% risk reduction for microvascular complications.[26] Given that this web-based intervention could be delivered at low-cost and at scale across the UK, the potential for population benefit is considerable. Moreover, in contrast to group-based education, where the effects appear to wane with time,[29] the effects of HeLP-Diabetes were greater at 12 months than at 3 months. There was no overall impact on diabetes-related distress, but some evidence that HeLP-diabetes appeared to reduce distress in recently diagnosed individuals. However, it is worth noting that baseline PAID scores were exceptionally low in this trial population. In a small pilot study, participants offered supported access to HeLP-Diabetes reduced their PAID scores by 6 points (p=0.04) over 6 weeks.[30]

The trial has many strengths. It was a pragmatic trial, open to nearly all patients with T2DM in participating practices. Concealment of allocation was complete, as randomisation occurred after baseline data collection. Baseline prognostic factors were well balanced between groups. Every effort was made to achieve blinding, including requiring practices to have two nurses, so that data collection was undertaken by a nurse blind to participant allocation. Data for the co-primary outcomes at the primary outcome point were available for 78% and 86% of participants for HbA1c and PAID respectively. All analyses were on an intention-to-treat basis, supplemented by a CACE analysis. Although response rates for the co-primary outcomes were good, some potential for bias existed. Our primary analysis used multiple imputation methods because evidence shows that the assumptions underpinning this method are more defensible than those assumed using other approaches to missing data.[31] We also undertook sensitivity analyses including complete cases, non-contaminated cases, and a linear model excluding centre; all yielded similar results.

The two co-primary outcomes reflected the twin aims of the intervention: to improve diabetes control and to reduce diabetes-related distress. Around 40% of patients with diabetes have significant levels of distress, which severely impacts on quality of life,[32] and diabetes-related distress is an important outcome for patients.[33] Our patient and public involvement (PPI) panel were clear that this should be a primary outcome, and a recent meta-ethnography emphasised the importance of empowerment and quality of life in promoting long term engagement with self-management.[34] In contrast, many health care professionals are more interested in glycaemic control. In line with previous trials in this area,[35] we decided to adopt both as co-primary outcomes and to test both at a 5% level of significance.[36]

There are some limitations. Despite maximising the inclusivity of the trial by minimising the exclusion criteria, participants were not representative of the overall population of patients with type 2 diabetes in England. Compared to the overall population, participants had better control of their diabetes and cardiovascular risk factors, [6 7] and were much less distressed. [21]

For peer review only - http://bmjopen.bmj.com/site/about/guidelines.xhtml

This finding mirrors that of a recent systematic review of demographic factors associated with web portal usage amongst people with diabetes which found that those with well controlled diabetes were more likely to use such portals than those with poor control. [37] However, fewer of our participants self-rated their computer skills as excellent (57% of our sample compared to a national average of 73%).[12] This good control at baseline has two implications – first, that there was little room for improvement in this population, and secondly, that this population may have been unusually motivated to self-manage their diabetes. Although every effort was made to maintain blinding, it is possible that some participants may have discussed their use of the intervention with research nurses, making it possible to infer which arm they had been allocated to. This could have affected secondary clinical outcomes, such as blood pressure or weight, but could not have affected assessment of glycated haemoglobin. There appeared to be high potential for contamination between two participants who shared the same surname and address, and a further two participants did not receive their allocated intervention due to an error at practice level; excluding these four made no difference to the results. A further limitation of the trial is that it provides little insight into the mechanism of action of HeLP-Diabetes. This was the result of a deliberate decision to focus on clinically important outcomes and minimise both the response burden and the potential impact of measurement on participants.

This is the first UK-based trial of a web-based self-management programme for people with type 2 diabetes, and internationally, the first trial of such a comprehensive intervention that aims to address the three main tasks of self-management: emotional, medical and role management.[19] In the Cochrane review of computer-based self-management interventions for people with T2DM, only 4 of the included studies had follow-up of 12 months or more.[14] Of these, three interventions were clinic-based, with participants completing self-assessment tools on a touch screen and receiving tailored advice during their baseline visit to their diabetes clinician [38-40] and one was a mobile phone-based intervention which provided tailored messages in response to participant's results of blood glucose self-monitoring data. [41] A more recent systematic review of internet delivered diabetes self-management identified 2 trials with 12 or more months follow-up. [42] One trial was on a structured intervention based on a peer-led, group-based, diabetes self-management course [43]. There were six sessions, with each session available for one week. Each session required participants to make a specific action plan to address a problem they were experiencing. Peer facilitators encouraged use of the programme. Follow up was planned at 6 and 12 months; however HbA1c data were only available at 6 months. The other trial compared two versions of a web-based intervention (with and without additional social support) to enhanced usual care. The web-based intervention was designed using social-cognitive theory and a social-ecological model, with a focus on three main behaviours: dietary intake, physical activity and medication adherence. Users of either web-based intervention received motivational phone calls to encourage adherence and development of action plans. Those randomised to the enhanced intervention (with additional social support) received two additional phone calls and an invitation to attend a group session. There was no statistically significant difference between groups in HbA1c or other biological outcomes at 12 months. [44] Thus the results of this trial add significantly to the available literature.

On the basis of these results, HeLP-Diabetes should be considered as an addition to the current menu of self-management support for people with type 2 diabetes, and may help increase overall access and uptake. Most commissioned services currently focus on newly diagnosed patients, leaving clear unmet need for people who have had their diabetes for longer, but are looking for ways to improve their health. Many patients are not ready to engage in self-management early in their illness journey,[9] but become motivated to do so later, often as a result of a change in medication or development of a complication.[45] The intervention is low cost, and as most costs are fixed, irrespective of number of users, is likely to be cost-effective, particularly if widely used. A cost-effectiveness analysis of HeLP-Diabetes will be reported separately.

For peer review only - http://bmjopen.bmj.com/site/about/guidelines.xhtml

Word count 4,303

Contributors

EM, MS, CD, KP, MB, AF, SM, LY, CM, SP, FS and DP all contributed to the design of the trial. MS, assisted by MH and KM designed the statistical analysis plan and undertook the analysis. SP and JL designed and undertook the health economic aspects of the trial. EM, KP, CD, JR, GA, LY and SM contributed to the development and delivery of the intervention. MK contributed PPI input. EM and MS wrote the first draft of the paper; all authors commented on this draft and have approved the final version.

Declaration of interests

EM is the Managing Director of a not-for-profit Community Interest Company established to disseminate HeLP-Diabetes across the UK.

Acknowledgements

Funding.

This paper presents independent research funded by the National Institute for Health Research (NIHR) under its Programme Grants for Applied Research Programme (Grant Reference Number RP-PG-0609-10135). The views expressed are those of the author(s) and not necessarily those of the NHS, the NIHR or the Department of Health. The funder had no role in the study design, data collection, data analysis, data interpretation, or writing of the report. All authors had full access to all the data in the study and can take responsibility for the integrity of the data and data analysis. The lead author had final responsibility for the decision to submit for publication, affirms that the manuscript is an honest, accurate, and transparent account of the study being reported; that no important aspects of the study have been omitted; and that there were no significant discrepancies from the published protocol.

Work conducted at the Cardiovascular Epidemiology Unit, University of Cambridge by MS and MH was additionally funded by the UK Medical Research Council (MR/L003120/1), British Heart Foundation (RG/13/13/30194) and UK National Institute for Health Research Cambridge Biomedical Research Centre.

AF is an NIHR Senior Investigator and receives funding from Oxford NIHR Biomedical Research Centre

We gratefully acknowledge the permission to use under license the validated behaviour change modules for weight loss (POWeR), alcohol reduction (DownYour Drink) and smoking cessation (StopAdvisor and the diabetes module from Healthtalk online (http://www.healthtalk.org/) in HeLP-Diabetes.

We are grateful to Orla O'Donnell for outstanding project management, Fiona Giles for administrative support, all our PPI who contributed to the development, maintenance and

For peer review only - http://bmjopen.bmj.com/site/about/guidelines.xhtml

delivery of the intervention and / or the management and oversight of the trial, staff at the participating practices, PCRN staff, and all our participants.

Data Sharing.

Patient level data, the full dataset, statistical code are available from the corresponding author. Consent for data sharing was not obtained from participants, but the potential benefits of sharing these data outweigh the potential harms as the data are anonymised.

For peer review only

Figure 1: CONSORT diagram.

ƚ Withdrawn from the trial before 60 days
ǂ Withdrawn from the trial between 60 and 304 days
¥ Withdrawn from the trial after 304 days
* Defined as a visit 60-120 days after randomisation
** Defined as a visit 300-420 days after randomisation
[a] Range of visits from 63 to 354 days after randomisation
[b] Range of visits from 50 to 253 days after randomisation

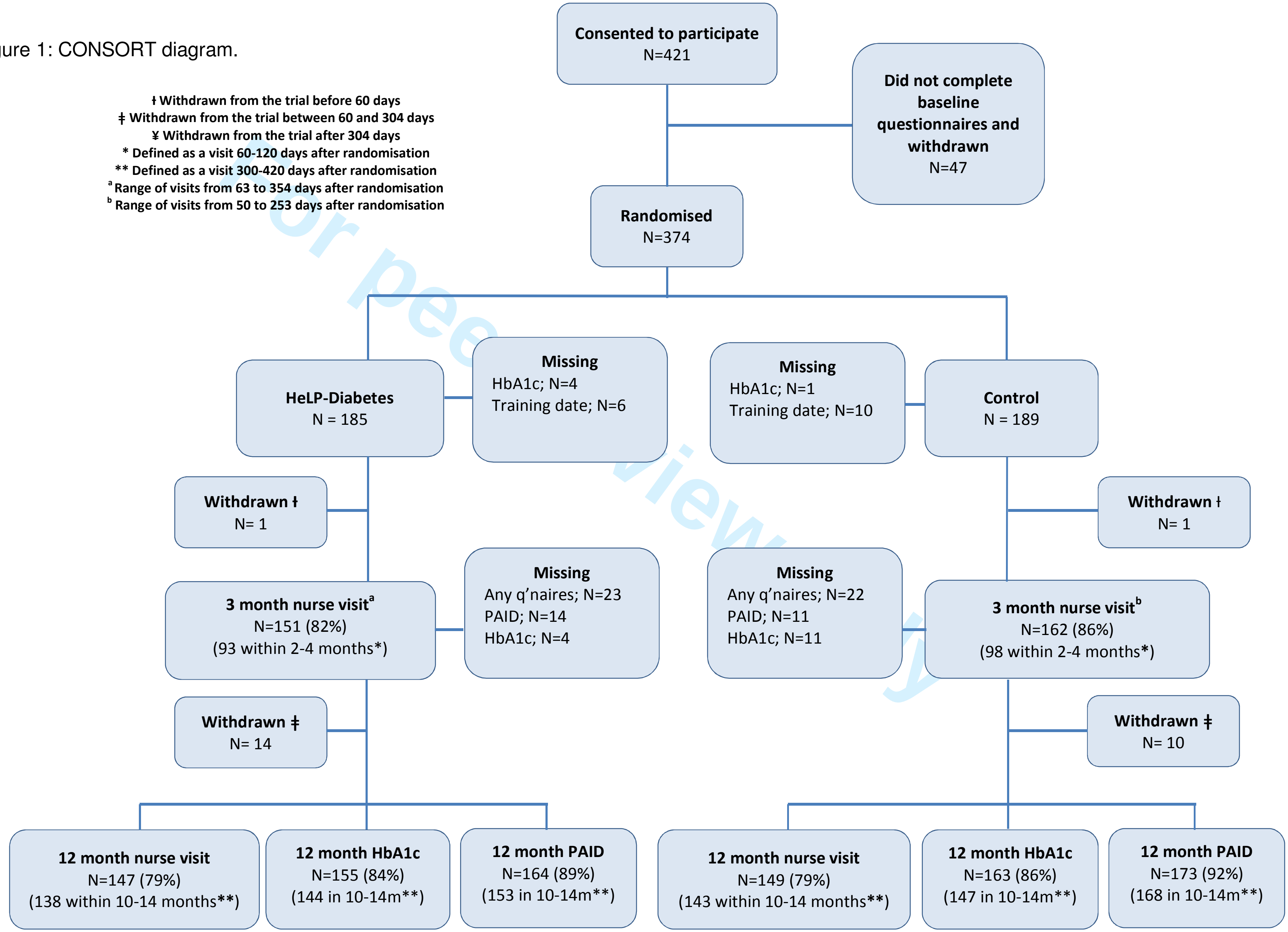

For peer review only

For peer review only - http://bmjopen.bmj.com/site/about/guidelines.xhtml

Figure 2. Mean HbA1c (95% confidence interval) over follow-up by randomised group following multiple imputation.

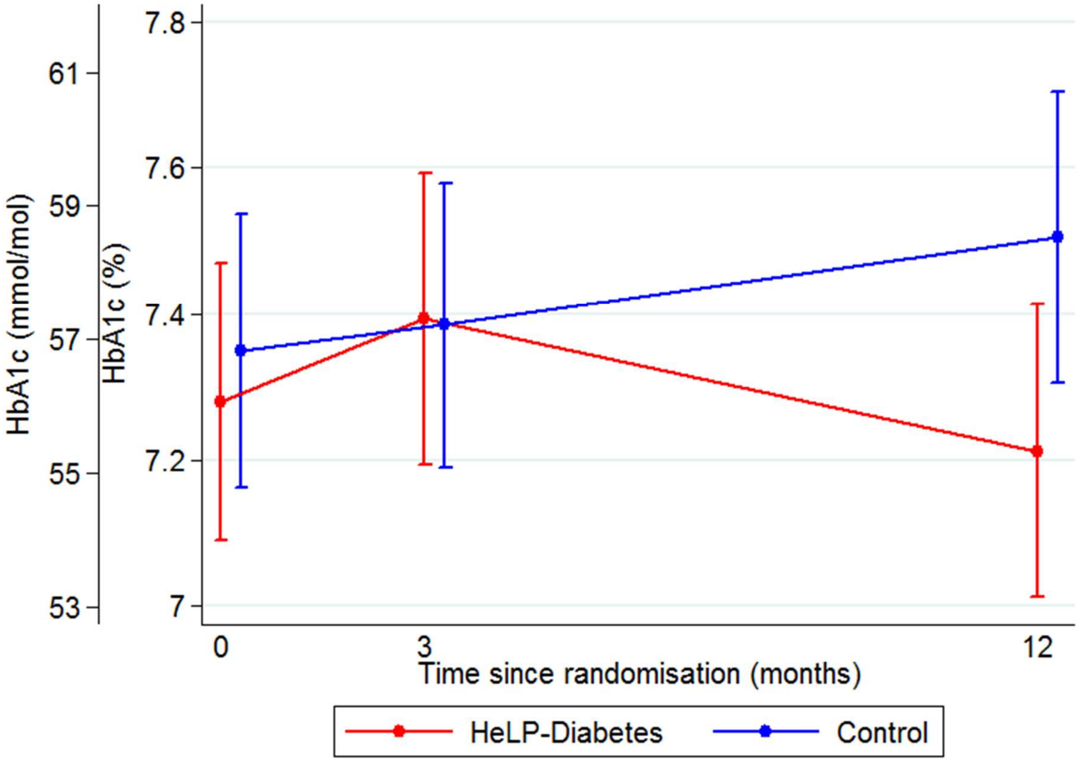

For peer review only

For peer review only - http://bmjopen.bmj.com/site/about/guidelines.xhtml

Figure 3. Mean PAID score (95% confidence interval) over follow-up by randomised group following multiple imputation

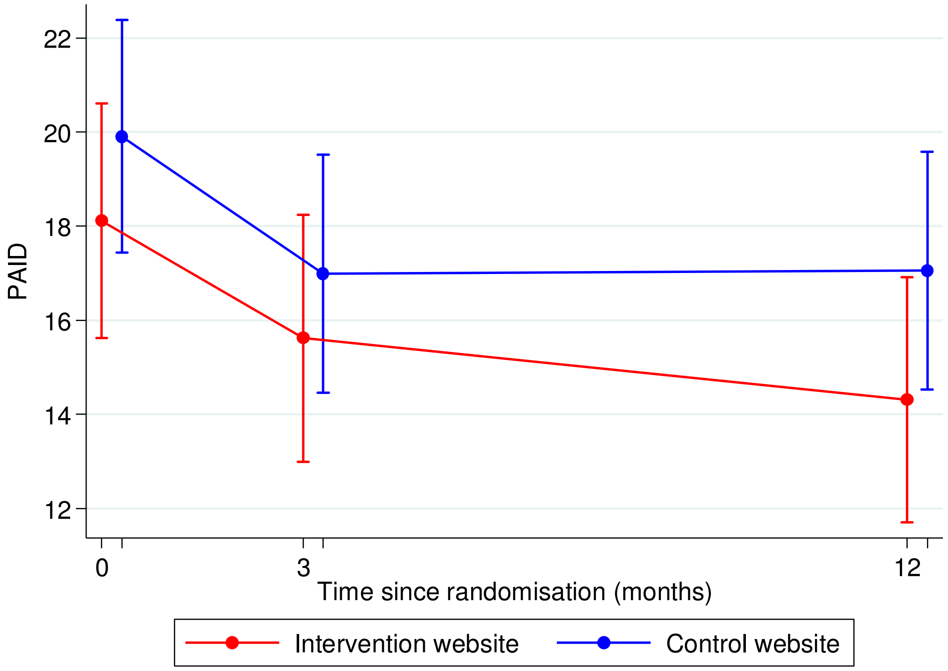

For peer review only - http://bmjopen.bmj.com/site/about/guidelines.xhtml

Table 1: Descriptive statistics of baseline variables by randomised group. Mean (SD) unless otherwise specified.

	HeLP-Diabetes N=185	Control N=189	N missing
Age at randomisation (yrs)	64.9 (9.5)	64.7 (9.1)	0
Male sex, n (%)	127 (69%)	131 (69%)	0
Ethnicity, n(%):			1
White English, Welsh, Scottish, Northern Irish, British	151 (82%)	149 (79%)	
Indian	12 (6%)	8 (4%)	
Other	21 (11%)	31 (16%)	
Experience with computers, n(%)			0
None	5 (3%)	4 (2%)	
Basic	75 (41%)	80 (42%)	
Experienced	105 (57%)	105 (56%)	
Smoking status, n(%):			0
Current smoker	14 (8%)	14 (7%)	
Former smoker	94 (51%)	86 (46%)	
Never smoker	77 (42%)	89 (47%)	
Time since diagnosis (years), n(%):			4
0-4 years	70 (38%)	64 (34%)	
5-9 years	55 (30%)	60 (32%)	
10-14 years	40 (22%)	40 (21%)	
15+ years	18 (10%)	23 (12%)	
Attending any other self-management class, n(%)	4 (2%)	4 (2%)	0
**Clinical Measures**			
Systolic blood pressure (mmHg)	135 (17)	135 (17)	0
Diastolic blood pressure (mmHg)	78 (11)	77 (10)	0
Total cholesterol (mmol/l)	4.11 (1.03)	4.18 (0.98)	2
HDL-C (mmol/l)	1.24 (0.31)	1.25 (0.36)	12
Total cholesterol /HDL-C ratio	3.43 (1.09)	3.52 (1.03)	13
HbA1c (%)	7.26 (1.25)	7.35 (1.37)	5
HbA1c (mmol/mol)	56 (14)	57 (15)	5
Body mass index (Kg/m^2)	30.1 (5.3)	29.6 (5.2)	2
**Questionnaires / scores**			
PAID (0-100)	18.1 (17.1)	19.9 (19.9)	0
HADS (0-42)	9.28 (6.47)	9.12 (7.52)	0
Anxiety scale (0-21)	4.92 (3.70)	5.21 (4.20)	0
Depression scale (0-21)	4.36 (3.48)	3.91 (3.73)	0
DMSES (0-150)	98.6 (33.9)	103.7 (32.4)	0
DTSQ (0-48)	32.1 (7.3)	32.0 (7.2)	0
Completion of 9 essential processes in previous 12 months, n(%)	97 (64%)	96 (62%)	69

For peer review only
For peer review only - http://bmjopen.bmj.com/site/about/guidelines.xhtml

Table 2: 12 month outcomes, adjusted for relevant baseline outcome, age, sex, current (baseline) participation in other self-management programmes, pre-existing cardiovascular disease and duration of diabetes.
Results from multiply imputed data shown. Data are mean (SE) or mean difference (95% CI) unless otherwise specified.

	HeLP-Diabetes		Control		HeLP-Diabetes vs Control	
	Baseline	Change from baseline to 12 months	Baseline	Change from baseline to 12 months	Mean difference (95% CI)	p-value
**Primary outcomes**						
HbA1c, (%)	7.3 (0.1)	-0.08 (0.07)	7.3 (0.1)	0.16 (0.07)	-0.24 (-0.44, -0.05)	0.014
HbA1c, mmol/mol	56.3 (1.1)	-0.8 (0.8)	56.8 (1.1)	1.8 (0.8)	-2.6 (-4.8, -0.5)	0.014
PAID	18.2 (1.3)	-4.1 (0.9)	19.8 (1.3)	-2.5 (0.9)	-1.5 (-3.9, 0.9)	0.209
**Secondary outcomes**						
Systolic blood pressure, mmHg	134.7 (1.5)	-4.2 (1.4)	134.9 (1.5)	-0.5 (1.4)	-3.8 (-6.6, -0.9)	0.010
Diastolic blood pressure, mmHg	77.8 (1.0)	-2.5 (0.9)	77.1 (1.0)	-1.9 (0.8)	-0.6 (-2.4, 1.2)	0.519
Body mass index, Kg/m^2	30.1 (0.5)	0.12 (0.2)	30.0 (0.5)	-0.04 (0.2)	0.16 (-0.30, 0.62)	0.498
Total cholesterol, mmol/L	4.1 (0.1)	-0.08 (0.06)	4.2 (0.1)	-0.15 (0.06)	0.07 (-0.09, 0.2)	0.370
HDL cholesterol, mmol/L	1.25 (0.03)	-0.003 (0.018)	1.26 (0.03)	0.004 (0.018)	-0.007 (-0.054, 0.039)	0.754
Completion of 9 essential processes*	65% (3.7)	-5.1%	61% (3.8)	3.4%	0.78 (0.45, 1.35)	0.379
HADS	9.3 (0.5)	-1.05 (0.44)	9.1 (0.5)	-0.60 (0.48)	-0.45 (-1.68, 0.78)	0.474
DMSES**	98.8 (2.4)	2.93 (2.90)	103.6 (2.3)	1.38 (2.79)	1.55 (-5.74, 8.84)	0.674
DTSQ	32.2 (0.6)	0.94 (0.57)	32.2 (0.6)	0.45 (0.61)	0.49 (-1.18, 2.15)	0.564

* Percentage (SE) and odds ratio (95% CI)
** linear regression results shown due to lack of convergence for mixed model

For peer review only

Table 3. Extent of website usage over 12-month follow-up.

	HeLP-Diabetes	Control	p-value*	N missing
Number of log-ins per person	18.7 (84.0)	4.8 (8.0)	0.0001	0
Number pages visited per log-in	10.5 (6.7)	7.7 (5.0)	<0.0001	105**
Time spent in each log-in (minutes)***	12.3 (9.8)	8.2 (8.4)	<0.0001	105**
Number of days in which website was accessed over follow-up	10.1 (22.9)	3.3 (5.1)	<0.0001	0

Mean (SD) unless otherwise specified.
* Wilcoxon rank-sum test.
** 105 individuals did not log in after their facilitation visit (42 intervention, 63 control).
*** Measured as time from first page accessed to last page accessed within a log-in session.

For peer review only

Reference List

1. Worldwide trends in diabetes since 1980: a pooled analysis of 751 population-based studies with 4.4 million participants. Lancet (London, England) 2016;**387**(10027):1513-30.
2. Tkac I. Effect of intensive glycemic control on cardiovascular outcomes and all-cause mortality in type 2 diabetes: Overview and metaanalysis of five trials. Diabetes Res Clin Pract 2009;**86 Suppl 1:S57-62.**:S57-S62.
3. National Institute of Health and Care Excellence (NICE). Type 2 diabetes in adults: Management. NG28. London, 2015.
4. Panagioti M, Richardson G, Murray E, et al. Self-management support interventions to reduce health care utilisation without compromising outcomes: a systematic review and meta-analysis. BMCHealth ServRes 2014;**14:356. doi: 10.1186/1472-6963-14-356.**:356-14.
5. (4) Foundations of care: education, nutrition, physical activity, smoking cessation, psychosocial care, and immunization. Diabetes care 2015;**38 Suppl**:S20-30.
6. NHS Digital. Quality and Outcomes Framework (QOF) - 2014-15. October 29 2015 ed. London: NHS Digital, 2015.
7. National Diabetes Audit 2013-2014 and 2014-2015 Report 1: Care Processes and Treatment Targets, 2016:1-35.
8. Deakin T, McShane CE, Cade JE, et al. Group based training for self-management strategies in people with type 2 diabetes mellitus. Cochrane Database SystRev 2005;**18**(2):CD003417.
9. Winkley K, Evwierhoma C, Amiel SA, et al. Patient explanations for non-attendance at structured diabetes education sessions for newly diagnosed Type 2 diabetes: a qualitative study. Diabetic medicine : a journal of the British Diabetic Association 2015;**32**(1):120-8.
10. Horigan G, Davies M, Findlay-White F, et al. Reasons why patients referred to diabetes education programmes choose not to attend: a systematic review. Diabetic medicine : a journal of the British Diabetic Association 2017;**34**(1):14-26.
11. Internet Access - Households and Individuals: 2015: Office for National Statistics, 2015:1-14.
12. Dutton WH, Blank G, Groselj D. Cultures of the Internet: The Internet in Britain. Oxford Internet Survery 2013. Oxford: Oxford Internet Institute, 2013:1 - 64.
13. Rowsell A, Muller I, Murray E, et al. Views of People With High and Low Levels of Health Literacy About a Digital Intervention to Promote Physical Activity for Diabetes: A Qualitative Study in Five Countries. Journal of medical Internet research 2015;**17**(10):e230.
14. Pal K, Eastwood SV, Michie S, et al. Computer-based diabetes self-management interventions for adults with type 2 diabetes mellitus. CochraneDatabaseSystRev 2013;**3:CD008776. doi: 10.1002/14651858.CD008776.pub2.**:CD008776.
15. van Vugt M, de Wit M, Cleijne WH, et al. Use of behavioral change techniques in web-based self-management programs for type 2 diabetes patients: systematic review. Journal of medical Internet research 2013;**15**(12):e279.
16. Hadjiconstantinou M, Byrne J, Bodicoat DH, et al. Do Web-Based Interventions Improve Well-Being in Type 2 Diabetes? A Systematic Review and Meta-Analysis. Journal of medical Internet research 2016;**18**(10):e270.
17. Murray E, Dack C, Barnard M, et al. HeLP-Diabetes: randomised controlled trial protocol. BMC health services research 2015;**15**:578.
18. Integrating theory, qualitative data and participatory design to develop HeLP-Diabetes: an internet self-management intervention for people with type 2 diabetes 2013/05/18/; Chicago, USA. International Society for Research on Internet Interventions.
19. Corbin JM, Strauss A. *Unending Work and Care*. First ed. San Francisco: Jossey-Bass Inc, 1988.
20. Polonsky WH, Anderson BJ, Lohrer PA, et al. Assessment of diabetes-related distress. Diabetes Care 1995;**18**(6):754-60.

For peer review only - http://bmjopen.bmj.com/site/about/guidelines.xhtml

For peer review only

21. Nicolucci A, Kovacs Burns K, Holt RI, et al. Diabetes Attitudes, Wishes and Needs second study (DAWN2): cross-national benchmarking of diabetes-related psychosocial outcomes for people with diabetes. Diabetic medicine : a journal of the British Diabetic Association 2013;**30**(7):767-77.
22. Zigmond AS, Snaith RP. The hospital anxiety and depression scale. Acta PsychiatrScand 1983;**67**(6):361-70.
23. Bijl JV, Poelgeest-Eeltink AV, Shortridge-Baggett L. The psychometric properties of the diabetes management self-efficacy scale for patients with type 2 diabetes mellitus. JAdvNurs 1999;**30**(2):352-59.
24. Bradley C, Lewis KS. Measures of psychological well-being and treatment satisfaction developed from the responses of people with tablet-treated diabetes. DiabetMed 1990;**7**(5):445-51.
25. Welch GW, Jacobson AM, Polonsky WH. The Problem Areas in Diabetes Scale. An evaluation of its clinical utility. Diabetes care 1997;**20**(5):760-6.
26. Stratton IM, Adler AI, Neil HA, et al. Association of glycaemia with macrovascular and microvascular complications of type 2 diabetes (UKPDS 35): prospective observational study. BMJ (Clinical research ed) 2000;**321**(7258):405-12.
27. White IR, Kalaitzaki E, Thompson SG. Allowing for missing outcome data and incomplete uptake of randomised interventions, with application to an Internet-based alcohol trial. Statistics in medicine 2011;**30**(27):3192-207.
28. Dunn G, Maracy M, Tomenson B. Estimating treatment effects from randomized clinical trials with noncompliance and loss to follow-up: the role of instrumental variable methods. StatMethods Med Res 2005;**14**(4):369-95.
29. Khunti K, Gray LJ, Skinner T, et al. Effectiveness of a diabetes education and self management programme (DESMOND) for people with newly diagnosed type 2 diabetes mellitus: three year follow-up of a cluster randomised controlled trial in primary care. BMJ 2012;**344:e2333. doi: 10.1136/bmj.e2333.**:e2333.
30. Hofmann M, Dack C, Barker C, et al. The Impact of an Internet-Based Self-Management Intervention (HeLP-Diabetes) on the Psychological Well-Being of Adults with Type 2 Diabetes: A Mixed-Method Cohort Study. Journal of diabetes research 2016;**2016**:1476384.
31. Molenberghs G, Thijs H, Jansen I, et al. Analyzing incomplete longitudinal clinical trial data. Biostatistics (Oxford, England) 2004;**5**(3):445-64.
32. Chew BH, Mohd-Sidik S, Shariff-Ghazali S. Negative effects of diabetes-related distress on health-related quality of life: an evaluation among the adult patients with type 2 diabetes mellitus in three primary healthcare clinics in Malaysia. Health and quality of life outcomes 2015;**13**:187.
33. Glasgow RE, Peeples M, Skovlund SE. Where is the patient in diabetes performance measures? The case for including patient-centered and self-management measures. Diabetes Care 2008;**31**(5):1046-50.
34. Frost J, Garside R, Cooper C, et al. A qualitative synthesis of diabetes self-management strategies for long term medical outcomes and quality of life in the UK. BMC health services research 2014;**14**:348.
35. Kinmonth AL, Woodcock A, Griffin S, et al. Randomised controlled trial of patient centred care of diabetes in general practice: impact on current wellbeing and future disease risk. The Diabetes Care From Diagnosis Research Team. BMJ (Clinical research ed) 1998;**317**(7167):1202-8.
36. Schulz KF, Grimes DA. Multiplicity in randomised trials I: endpoints and treatments. Lancet (London, England) 2005;**365**(9470):1591-5.
37. Amante DJ, Hogan TP, Pagoto SL, et al. A systematic review of electronic portal usage among patients with diabetes. Diabetes technology & therapeutics 2014;**16**(11):784-93.

For peer review only - http://bmjopen.bmj.com/site/about/guidelines.xhtml

38. Christian JG, Bessesen DH, Byers TE, et al. Clinic-based support to help overweight patients with type 2 diabetes increase physical activity and lose weight. Arch Intern Med 2008;**168**(2):141-6.
39. Glasgow RE, La Chance PA, Toobert DJ, et al. Long term effects and costs of brief behavioural dietary intervention for patients with diabetes delivered from the medical office. Patient education and counseling 1997;**32**(3):175-84.
40. Glasgow RE, Nutting PA, King DK, et al. Randomized effectiveness trial of a computer-assisted intervention to improve diabetes care. Diabetes care 2005;**28**(1):33-9.
41. Quinn CC, Shardell MD, Terrin ML, et al. Cluster-randomized trial of a mobile phone personalized behavioral intervention for blood glucose control. Diabetes care 2011;**34**(9):1934-42.
42. Pereira K, Phillips B, Johnson C, et al. Internet delivered diabetes self-management education: a review. Diabetes technology & therapeutics 2015;**17**(1):55-63.
43. Lorig K, Ritter PL, Laurent DD, et al. Online diabetes self-management program: a randomized study. Diabetes Care 2010;**33**(6):1275-81.
44. Glasgow RE, Kurz D, King D, et al. Twelve-month outcomes of an Internet-based diabetes self-management support program. Patient education and counseling 2012;**87**(1):81-92.
45. Shirazian S, Crnosija N, Weinger K, et al. The self-management experience of patients with type 2 diabetes and chronic kidney disease: A qualitative study. Chronic illness 2016;**12**(1):18-28.

For peer review only

For peer review only - http://bmjopen.bmj.com/site/about/guidelines.xhtml

Appendix 1: description of the intervention.[1]

Title: Healthy Living for People with type 2 Diabetes (HeLP-Diabetes): a web-based self-management programme for people with type 2diabetes.

Rationale

The aim of HeLP-Diabetes was to enable people with type 2 diabetes to lead healthier lives and improve overall quality of life. In order to do this, we adopted a strong theoretical framework, which included:

1. The Corbin and Strauss model of living with a long term condition,[2] which determined the overall scope of the intervention;
2. Normalisation Process Theory,[3] to maximise likelihood of successful implementation of the intervention into routine health care
3. A taxonomy of behaviour change techniques,[4] to guide the content of the behaviour change modules;
4. Finally, we worked within the paradigm of evidence-based medicine, ensuring all content was congruent with current best evidence. This applied both to the medical content (e.g. information about treatments) and to the delivery of the intervention (e.g. evidence about maximising acceptability, uptake and adherence).

Self-management of a long term condition is a complex process, which Corbin and Strauss characterised as requiring medical, emotional and role management.[2] Medical management includes behaviour change, such as eating healthily, being physically active, stopping smoking and taking medication. Working with health professionals and the health care system (e.g. keeping appointments, remembering to schedule check-ups or regular monitoring tests) is also included in medical management. Equally important, and often just as challenging for individuals concerned, are emotional management and role management. Emotional management refers to managing the complex negative emotions that result from being diagnosed with a long term condition, such as depression, anger, guilt, shame and a sense of stigma. Role management requires adjusting to the change in biographical narrative, acceptance of the role of "patient", and negotiating inevitable changes in relationships with family, friends and colleagues. This characterisation of the work of self-management provided the overall framework for the content, tone and style of the intervention. As these tasks are intertwined and inter-dependent, we designed the content to address all three tasks in an integrated fashion.

Normalisation Process Theory is a mid-range sociological theory which predicts and explains whether and why certain innovations will be effectively implemented into routine health care and become "normalised", that is, become so much part of routine practice that they disappear from view.[3] It posits that interventions which are easily understood and distinguished from other interventions (coherence), which relevant professionals can see the advantage of compared to current or alternative practice (cognitive participation), which fit well with existing professional relationships and workflows (collective action), and where users are provided with formal or informal feedback and evidence about the impact of the intervention (reflexive monitoring) are more likely to be implemented than those that do not. These constructs guided the development of the intervention by, for example, ensuring HeLP-Diabetes integrated well with existing workflows in general practice.

Effective self-management in diabetes often requires patients to change deep-rooted behaviours, particularly around diet and physical activity. Rather than adopt a single behavioural theory, we opted to design our behaviour change modules around specific

For peer review only - http://bmjopen.bmj.com/site/about/guidelines.xhtml

behaviour change techniques, selecting those with the best evidence base to support their use.[4]

Content

The overall content was broken down into 8 sections:

1. Understanding diabetes (information about the nature and causes of diabetes, and how it affects the body);
2. Staying healthy (motivational material about how to maintain optimal physical and emotional health and the importance of self-management; new behaviour change modules and previously validated programmes for diet, weight loss, physical activity, smoking cessation, moderating alcohol intake, and taking medicines);
3. Treating diabetes (information about medications used in diabetes, including information about indications, side effects and monitoring; importance of managing cardiovascular risk factors as well as glycaemic levels; importance of regular monitoring to prevent retinopathy, neuropathy and nephropathy; and types and roles of different health care professionals involved in caring for people with diabetes);
4. Living and working with diabetes (focus on managing social and work situations, such as shift work, parties, or holidays; impact on relationships, including sexual relationships; and possible impact on emotions and feelings of self-worth);
5. Managing my feelings (self-assessment tools for identifying low mood; cognitive behavioural therapy modules; mindfulness-based approaches);
6. My health record (opportunity to record appointments with health care professionals, results of tests or self-monitoring, with opportunities for graphical displays and feedback);
7. News and Research (updates about latest research or news about diabetes treatment, in depth articles about seminal research papers);
8. Forum and Help (moderated forum; videos of personal stories about diabetes (used with license from health talk online); additional resources, with local resources tailored according to CCG).

Where possible we aimed to use existing modules with a proven evidence-base. Hence we used, with licence, the entire type 2 diabetes module from Health Talk Online, which contains videos of interviews with people with diabetes from a range of socio-economic and ethnic backgrounds, talking about how they were diagnosed, the impact diabetes has had on their lives and relationships, how they have adapted or come to terms with it, and treatments they have experiences (see www.healthtalk.org). Behaviour change programmes used with license included: POWeR (Positive Online Weight Reduction), a set of 12 modules shown to be effective for managing obesity in primary care[5]; Stop Advisor (smoking cessation)[6] and Down Your Drink (alcohol moderation).[7] New modules were developed for healthy eating, increasing physical activity and taking medication. These used the behaviour change techniques of action planning, goal setting, self-monitoring, reminders and feedback, along with information about the benefits of change. The section on "Managing my feelings" included the *Living Life to the Full* programme[8] – a set of 10 modules based on the principals of cognitive behavioural therapy, specifically adapted for people with type 2 diabetes, and used with license.

Procedures

For peer review only - http://bmjopen.bmj.com/site/about/guidelines.xhtml

HeLP-Diabetes was designed to be used as part of an overall package of care for people with diabetes. Low usage, or non-adherence to internet interventions is well-recognised problem, and our preparatory work with patients and health care professionals indicated that integrating the intervention into routine care was likely to improve uptake and adherence. Hence we made the programme available to registered users only, and encouraged health care professionals to register patients. Once registered, patients could use the programme as much (or as little) as they wanted. There was no prescribed level of use, as our proposed users included patients at all stages of their illness journey, from those newly diagnosed to those who had lived with diabetes for many years. As such, we anticipated that each user would have different needs and priorities, and the programme was designed to allow users to pick and choose sections that were most relevant and beneficial for them personally. There was a limited amount of tailoring. Additional resources and sources of help were tailored by the patient's CCG, but otherwise tailoring was limited to the behaviour change and health record sections where users entered their own goals or data.

Registration and facilitation.

During the trial, registration was performed by a practice nurse. During the registration procedures users were asked to select a password and username. After registration, the nurse was asked to demonstrate the intervention to the patient, and integrate use of the intervention into the patient's personal diabetes care plan by, for example, discussing what goals the patient would like to set for the coming period, and showing the patient how to use the intervention to set goals, monitor progress, and, if desired, arrange for automated reminders by SMS or email to be sent by the programme.

Registration was undertaken in the patient's practice. All subsequent use of the intervention was at any location convenient for the patient with internet access. For most people, we expected this to be at home, or at the home of a relative. However, all users were given information about local services (usually libraries) offering free access to an internet-connected computer.

Encouraging engagement

In addition to the registration and facilitation procedures described above, engagement to the programme was encouraged through regular prompts, provided by email or SMS. Emailed prompts were of two types: a) short (2 – 3 lines) emails with one message and a link to the relevant section of the programme. An example of this type of prompt was a seasonal reminder of the importance of flu vaccination for people with diabetes, with a link to the page of the programme explaining how and why flu vaccination is beneficial, and a reminder that flu vaccination is available free from their general practice. The second type of email prompt was a longer (1 page of A4) newsletter, which summarised recent research or news stories, explained what this meant for patients, and also contained a paragraph or two on specific health promotion – for example, the importance of regular foot care, how to fast safely during Ramadan, or tips for safe overseas travel. These stories and paragraphs also contained links to relevant sections of the programme. SMS prompts were very brief, with one clear message, and a link to the programme. We sent around 3 prompts per month – usually 2 short emails or SMS plus one longer newsletter. Users could opt out of receiving prompts.

For peer review only - http://bmjopen.bmj.com/site/about/guidelines.xhtml

Modifications during the trial

One of the key functions of the programme was to provide up-to-date, evidence-based information. Hence the site was regularly reviewed to ensure all content was up-to-date, evidence-base, and congruent with current NICE guidelines. In practice, this meant small updates each month, with a complete review when the NICE guidelines on management of diabetes were updated.[9]

Assessment of fidelity

Intervention use was assessed through bespoke software which recorded the date, time and pages viewed for each log in by each user. Practice nurses were trained in registration and facilitation procedures, but we were unable to monitor how well they adhered to them.

References

1. Hoffmann TC, Glasziou PP, Boutron I, et al. Better reporting of interventions: template for intervention description and replication (TIDieR) checklist and guide. *BMJ (Clinical research ed)* 2014; **348**: g1687.
2. Corbin JM, Strauss A. Unending Work and Care. First ed. San Francisco: Jossey-Bass Inc; 1988.
3. May C, Finch T. Implementation, embedding, and integration: an outline of Normalization Process Theory. *Sociology* 2009; **43**(3): 535-54.
4. Abraham C, Michie S. A taxonomy of behavior change techniques used in interventions. *Health Psychol* 2008; **27**(3): 379-87.
5. Yardley L, Ware LJ, Smith ER, et al. Randomised controlled feasibility trial of a web-based weight management intervention with nurse support for obese patients in primary care. *The international journal of behavioral nutrition and physical activity* 2014; **11**: 67.
6. Brown J, Michie S, Geraghty AW, et al. Internet-based intervention for smoking cessation (StopAdvisor) in people with low and high socioeconomic status: a randomised controlled trial. *The Lancet Respiratory medicine* 2014; **2**(12): 997-1006.
7. Wallace P, Murray E, McCambridge J, et al. On-line Randomized Controlled Trial of an Internet Based Psychologically Enhanced Intervention for People with Hazardous Alcohol Consumption. *PLoSONE* 2011; **6**(3): e14740.
8. Williams C, Association CMH. Living life to the full: Media innovations; 2008.
9. NICE. Type 2 diabetes in adults: Management. NG28. London, 2015.

For peer review only - http://bmjopen.bmj.com/site/about/guidelines.xhtml

174 TiDIER CHECKLIST.

Item	Line numbers
Provide the name or a phrase that describes the intervention	2,3
Describe any rationale, theory or goal of the elements essential to the intervention	4 – 48
Materials: describe any physical or informational materials used in the intervention, including those provided to participants or used in intervention delivery or in training of the intervention providers	51-90
Procedures: Describe each of the procedures, activities and /or processes used in the intervention, including any enabling or support activities	92 - 137
For each category of intervention provider, describe their expertise, background, and any specific training given.	109
Describe the modes of delivery (such as face-to-face or by some other mechanism, such as internet or telephone) of the intervention, and whether it was provided individually or in a group	2, 123 - 137
Describe the type(s) of location(s) where the intervention occurred, including any necessary infrastructure or relevant features	116 - 120
Describe the number of times the intervention was delivered and over what period of time including the number of sessions, their schedule and their duration, intensity or dose.	98 - 106
If the intervention was planned to be personalised, titrated or adapted, then describe what, why, when and how.	101 - 106
If the intervention was modified during the course of the study, describe the changes (what, why, when and how)	139 - 144
Planned: if intervention adherence or fidelity was assessed, describe how and by whom, and if any strategies were used to maintain or improve fidelity, describe them.	147 - 149
Actual: if intervention adherence or fidelity was assessed, describe the extent to which the intervention was delivered as planned.	147 - 149

175

For peer review only

For peer review only - http://bmjopen.bmj.com/site/about/guidelines.xhtml

Appendix 2: Additional statistical methods and results

Statistical Methods for Multiple Imputation

Multiple Imputation using chained equation was used as the primary method to account for missing data (in both baseline and follow-up data).[1] A set of imputation models were specified, one for each variable with missing data. Each variable was then regressed on all other variables, including completely recorded baseline and follow-up variables and stratified by randomised group. Imputations were performed using predictive mean matching using the five nearest neighbours to the prediction as a set to draw from. The full list of variables considered in the MICE approach is shown in Supplementary Table 1, together with the number of missing values for each variable and time period.

Since only measurements within a 10-14 month window period were used within the main analyses of HbA1c and PAID, the following imputation procedure was implemented for these two co-primary outcomes. Twelve month measurements were subdivided into those that were measured within 10-14 months (the primary outcome variable) and those that were measured outside 10-14 months (a variable used for imputing only). For HbA1c, two additional variables were created for use within the imputation model; 1) the time in days from randomisation that the “12-month” HbA1c measurement was actually taken for values inside of the window period (and set to 365 for measurements taken outside the window period), 2) the time in days from randomisation that the “12-month” HbA1c measurement was actually taken for values outside the window period (and set to missing for measurements taken within the window period). The first of these variables gives the desired time for imputing HbA1c measurements when they are missing, whilst the second gives information on how far outside of the window the actual measurements were taken. Corresponding variables were created for the “12-month” PAID measurement. Finally, two additional variables were created defining the time in days at which HbA1c and PAID were measured at “3-months”. All variables were included within the chained equations and imputed where necessary.

40 imputed datasets were created, the analysis models were fitted to each imputed dataset separately, and the estimates were pooled using Rubin’s rules.

Statistical Methods for causal analyses

The causal analysis proposed attempts to address how the effectiveness of the intervention is mediated through the frequency of website usage. In particular, it is important to understand whether prolonged usage of the website modifies the efficacy of the intervention. Since website usage is measured post-randomisation a naïve analysis of correlating usage with outcomes in the intervention group may give biased and misleading results, since there may be unmeasured confounders also correlated with the outcomes that distinguish the motivated users who regularly log-in from the less motivated ones. Causal analyses using instrumental variables (IV) were therefore used to determine the effect of website usage on outcomes. This approach preserves randomisation (i.e. provides a comparison independent of observed and unobserved confounders).

“Usage” is defined as the proportion of follow-up (rescaled as no. days in a year) that the HeLP-Diabetes website is accessed. It was assumed that the efficacy of the intervention is zero for individuals who never log-in (the exclusion restriction assumption). Website usage in the control group was ignored in the model as it was assumed that the control website was unlikely to be effective. (NB. usage statistics were collected for the comparator website but were not used in these analyses).

For peer review only

This main underlying assumption of the causal analysis is that the effect of randomisation to the HeLP-Diabetes intervention on 12-month outcomes occurs only through use of the website (Supplementary Figure 1; Z is randomised intervention, WU is website-usage and Y is 12-month outcome, e.g. HbA1c or PAID). This relies of the “exclusion restriction” assumption that the HeLP Diabetes intervention has no effect when usage is zero (i.e. for individuals who never log-in). Hence randomisation is assumed to be an instrumental variable.

Figures 4 and 5 show the estimated causal effects of HeLP Diabetes on HbA1c and PAID at 12-months, respectively, by level of website usage (the “exclusion restriction” assumption leads to zero efficacy at zero usage). To determine the casual estimate of efficacy for a “high-usage” group, the individual predicted efficacy was calculated for each individual greater than or equal to the median usage of 4 days (shown as the red boxes in the histograms in Figures 4 and 5) and the average efficacy for this group was obtained. The mean usage in the “high-usage” group was 18 days.

Supplementary Figure 1. Causal model assuming no direct effect of intervention (complete mediation through website usage).

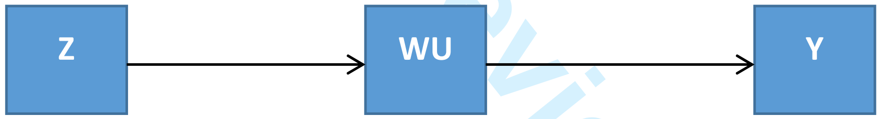

In this causal model, randomisation (Z) is the instrumental variable, which acts on the outcome (Y) exclusively through website usage (WU).

For peer review only

Supplementary Figure 2: Efficacy of HeLP-Diabetes on HbA1c at 12-months based on number of days usage, superimposed by the distribution of usage observed in the HeLP-Diabetes group. The bars shown in red highlight the "high-usage" group that have usage greater than or equal to the median of 4 days.

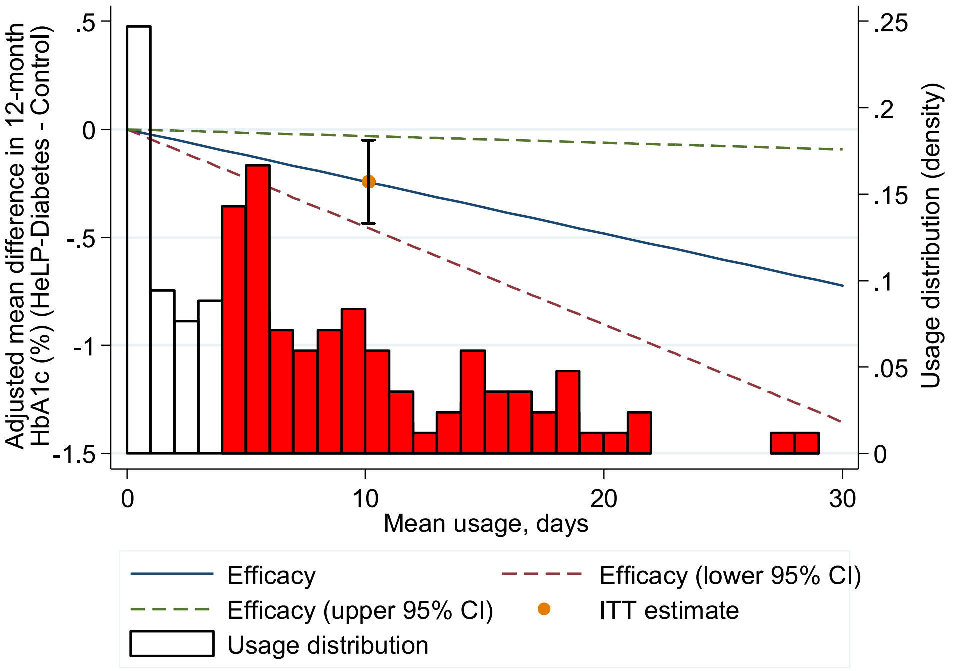

Supplementary Figure 3. Efficacy of HeLP-Diabetes on PAID score at 12-months based on number of days usage, superimposed by the distribution of usage observed in the HeLP-Diabetes group. The bars shown in red highlight the "high-usage" group that have usage greater than or equal to the median of 4 days.

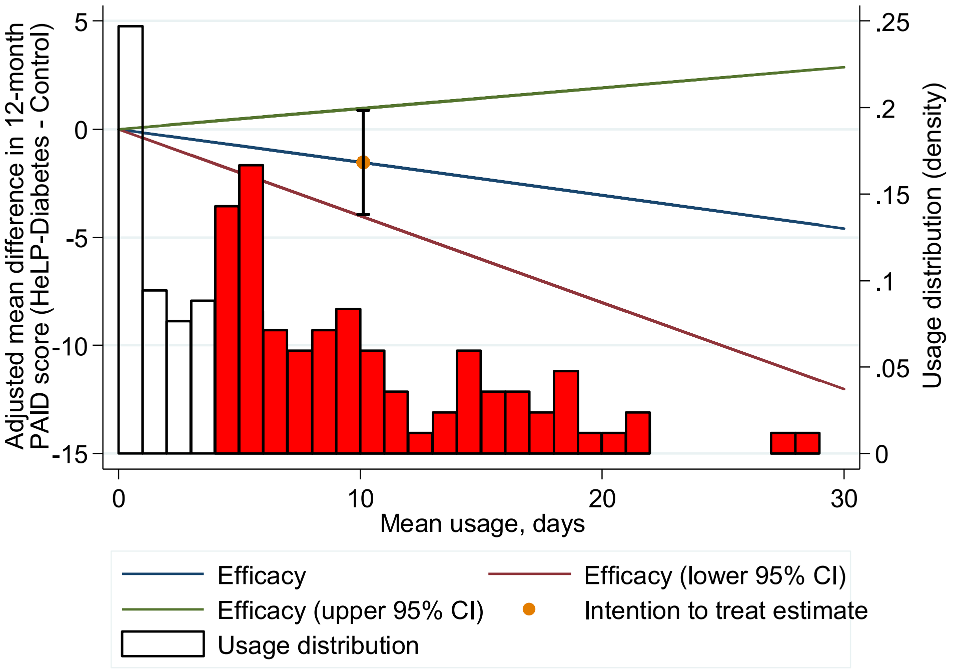

For peer review only

Supplementary Figure 4. Means and 95% CIs of HbA1c over time by randomised group and completers vs. non-completers, after multiple imputation of non-completers follow-up

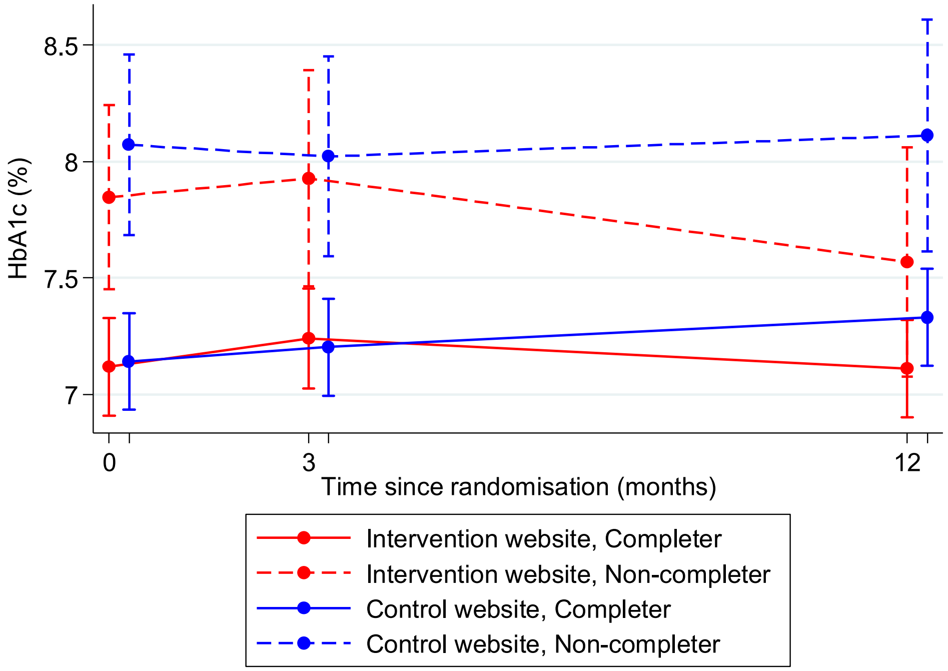

view only

Supplementary Table 1. List of variables imputed and amount of missing data at baseline, 3 months and 12 months.

Variable	Number of missing values		
	Baseline	3 months	12 months
HbA1c	5	64	83 10-14months †
			347 Outside 10-14months †
PAID	0	54	53 10-14months †
			358 Outside 10-14months †
Systolic Blood Pressure	0	57	68
Diastolic Blood Pressure	0	57	68
Body Mass Index	2	58	69
Total cholesterol	2	68	75
HDL cholesterol	12	73	76
Completion of 9 essential processes	69	-	69
HADS	0	74	107
DMSES	0	73	109
DTSQ	0	77	109
Age	0	-	-
Sex	0	-	-
Duration of diabetes	4	-	-
History of cardiovascular disease	0	-	-
Attending any other self-management class	0	-	-
Smoking status	0	-	-
No of visits to the website	-	-	0
No of webpages visited	-	-	105
Average time per visit	-	-	105

* Partial mean matching performed using 5 nearest neighbours

† 12-month measurements were subdivided into those that were measured within 10-14 months (primary outcome variable) and those that were measured outside 10-14 months (variable used for imputing only).

For peer review only

Supplementary Table 2: Descriptive statistics of 3-month variables by randomised group. Mean (SD) unless otherwise specified.

	HeLP-Diabetes	Control	p-value*	N missing
**Clinical Measures**				
Systolic blood pressure (mmHg)	133 (16)	134 (17)	0.350	57
Diastolic blood pressure (mmHg)	76 (10)	75 (9)	0.699	57
Total cholesterol (mmol/l)	4.17 (0.97)	4.16 (1.04)	0.900	68
HDL-C (mmol/l)	1.29 (0.37)	1.24 (0.37)	0.356	73
Total cholesterol /HDL-C ratio	3.48 (1.15)	3.52 (1.09)	0.570	74
HbA1c (%)	7.34 (1.48)	7.34 (1.24)	0.323	64
Body mass index (Kg/m^2)	30.0 (5.4)	30.0 (5.6)	0.545	58
**Questionnaires / scores**				
PAID (0-100)	15.2 (15.2)	16.7 (17.1)	0.492	54
HADS (0-42)	8.84 (6.54)	8.86 (7.45)	0.603	74
Anxiety scale (0-21)	4.75 (3.88)	5.07 (4.24)	0.639	74
Depression scale (0-21)	4.10 (3.30)	3.80 (3.59)	0.205	74
DMSES (0-150)	103.9 (32.2)	104.9 (32.7)	0.795	73
DTSQ (0-48)	32.8 (6.9)	32.9 (6.3)	0.715	77

* Wilcoxon rank-sum test

For peer review only

Supplementary Table 3. Effect of HeLP-Diabetes vs. Control on 12-month primary outcomes, HbA1c and PAID. Results from primary analyses and five sensitivity analyses

	HbA1c, %	
	Number individuals	mean difference (95% CI) HeLP-Diabetes - Control
Primary Analysis<br>Multiple Imputation of both baseline and outcomes	374	-0.242 (-0.435, -0.049); p=0.014
Sensitivity Analysis 1:<br>(multiple imputation of baseline covariates only)	291	-0.214 (-0.390, -0.038); p=0.017
Sensitivity Analysis 2:<br>(complete cases) ł	284	-0.207 (-0.385, -0.028); p=0.023
Sensitivity Analysis 3:<br>(complete cases) ǂ	186	-0.220 (-0.416, -0.025); p=0.027
Sensitivity Analysis 4:<br>(non-contaminated cases) ¥	370	-0.225 (-0.418, -0.032); p=0.023
Sensitivity Analysis 5:<br>Linear model excluding centre*	374	-0.242 (-0.438, -0.047); p=0.015
	**PAID**	
	Number individuals	mean difference (95% CI) HeLP-Diabetes - Control
Primary Analysis<br>Multiple Imputation	374	-1.54 (-3.94, 0.87); p=0.209
Sensitivity Analysis 1:<br>(multiple imputation of baseline covariates only)	321	-1.31 (-3.65, 1.03); p=0.274
Sensitivity Analysis 2:<br>(complete cases) ł	317	-1.29 (-3.66, 1.08); p=0.285
Sensitivity Analysis 3:<br>(complete cases) ǂ	65	-2.22 (-6.35, 1.91); p=0.292
Sensitivity Analysis 4:<br>(non-contaminated cases) ¥	370	-1.57 (-3.99, 0.85); p=0.204
Sensitivity Analysis 5:<br>Linear model excluding centre**	374	-1.54 (-3.98, 0.90); p=0.215

ł excluding outcomes outside 10-14 months post-randomisation and those missing baseline covariates

ǂ excluding outcomes outside 11-13 months post-randomisation and those missing baseline covariates

¥ Excluding patients who were suspected to have been exposed to the alternative intervention

* likelihood ratio test for including centre in the model as a fixed effect; p=0.617 (HbA1c)

** likelihood ratio test for including centre in the model as a fixed effect; p=0.357 (PAID)

For peer review only

Supplementary Table 4. Effect of subgroups on the effectiveness of HeLP-Diabetes in reducing HbA1c and PAID.

Subgroup	HbA1c, %			
	Number individuals	mean difference (95% CI) HeLP-Diabetes - Control		Interaction p-value*
		Complete Case ɫ	Multiple Imputation	
Baseline HbA1c				0.458
<7.5%	254/369	-0.15 (-0.41, 0.11)	-0.14 (-0.39, 0.12)	
≥7.5%	115/369	-0.51 (-0.94, -0.07)	-0.51 (-0.93, 0.09)	
Duration of diabetes				0.425
<6.9 yrs	185/370	-0.34 (-0.59, -0.08)	-0.30 (-0.57, -0.03)	
≥6.9 yrs	185/370	-0.07 (-0.32, 0.18)	-0.18 (-0.45, 0.09)	
	**PAID**			
Baseline PAID				0.066
<12	181/374	-0.3 (-4.6, 4.1)	-0.1 (-4.2, 4.1)	
≥12	193/374	-3.6 (-7.8, 0.7)	-3.9 (-8.2, 0.4)	
Duration of diabetes				0.004
<6.9 yrs	185/370	-2.9 (-6.3, 0.4)	-3.6 (-7.0, -0.1)	
≥6.9 yrs	185/370	0.3 (-3.0, 3.7)	0.4 (-3.1, 3.9)	

ɫ excluding outcomes outside 10-14 months post-randomisation and those missing baseline covariates

* p-value for interaction based on a test of linear trend with the continuous variable.

Reference List

1. White IR, Royston P, Wood AM. Multiple imputation using chained equations: Issues and guidance for practice. *Statistics in medicine* 2011; **30**(4): 377-99.

For peer review only

CONSORT 2010 checklist of information to include when reporting a randomised trial*

Section/Topic	Item No	Checklist item	Reported on page No
**Title and abstract**			
	1a	Identification as a randomised trial in the title	1
	1b	Structured summary of trial design, methods, results, and conclusions (for specific guidance see CONSORT for abstracts)	2
**Introduction**			
Background and objectives	2a	Scientific background and explanation of rationale	4
	2b	Specific objectives or hypotheses	4
**Methods**			
Trial design	3a	Description of trial design (such as parallel, factorial) including allocation ratio	4
	3b	Important changes to methods after trial commencement (such as eligibility criteria), with reasons	4
Participants	4a	Eligibility criteria for participants	4
	4b	Settings and locations where the data were collected	4
Interventions	5	The interventions for each group with sufficient details to allow replication, including how and when they were actually administered	5 + Appendix 1
Outcomes	6a	Completely defined pre-specified primary and secondary outcome measures, including how and when they were assessed	6
	6b	Any changes to trial outcomes after the trial commenced, with reasons	N/a
Sample size	7a	How sample size was determined	6-7
	7b	When applicable, explanation of any interim analyses and stopping guidelines	N/A
Randomisation:			
Sequence generation	8a	Method used to generate the random allocation sequence	5
	8b	Type of randomisation; details of any restriction (such as blocking and block size)	5
Allocation concealment mechanism	9	Mechanism used to implement the random allocation sequence (such as sequentially numbered containers), describing any steps taken to conceal the sequence until interventions were assigned	5
Implementation	10	Who generated the random allocation sequence, who enrolled participants, and who assigned participants to interventions	5
Blinding	11a	If done, who was blinded after assignment to interventions (for example, participants, care providers, those	5

For peer review only - http://bmjopen.bmj.com/site/about/guidelines.xhtml

		assessing outcomes) and how	5
	11b	If relevant, description of the similarity of interventions	6
Statistical methods	12a	Statistical methods used to compare groups for primary and secondary outcomes	7
	12b	Methods for additional analyses, such as subgroup analyses and adjusted analyses	7 + Appendix 2
**Results**			
Participant flow (a diagram is strongly recommended)	13a	For each group, the numbers of participants who were randomly assigned, received intended treatment, and were analysed for the primary outcome	7 + Figure 1
	13b	For each group, losses and exclusions after randomisation, together with reasons	Figure 1
Recruitment	14a	Dates defining the periods of recruitment and follow-up	7
	14b	Why the trial ended or was stopped	N/A
Baseline data	15	A table showing baseline demographic and clinical characteristics for each group	Table 1
Numbers analysed	16	For each group, number of participants (denominator) included in each analysis and whether the analysis was by original assigned groups	Figure 1
Outcomes and estimation	17a	For each primary and secondary outcome, results for each group, and the estimated effect size and its precision (such as 95% confidence interval)	8, Table 2, Figures 2 & 3
	17b	For binary outcomes, presentation of both absolute and relative effect sizes is recommended	N/A
Ancillary analyses	18	Results of any other analyses performed, including subgroup analyses and adjusted analyses, distinguishing pre-specified from exploratory	8, Supplementary Table 4
Harms	19	All important harms or unintended effects in each group (for specific guidance see CONSORT for harms)	9
**Discussion**			
Limitations	20	Trial limitations, addressing sources of potential bias, imprecision, and, if relevant, multiplicity of analyses	9 - 10
Generalisability	21	Generalisability (external validity, applicability) of the trial findings	9 - 10
Interpretation	22	Interpretation consistent with results, balancing benefits and harms, and considering other relevant evidence	9 - 10
**Other information**			
Registration	23	Registration number and name of trial registry	7
Protocol	24	Where the full trial protocol can be accessed, if available	Reference 17, page 22
Funding	25	Sources of funding and other support (such as supply of drugs), role of funders	11

*We strongly recommend reading this statement in conjunction with the CONSORT 2010 Explanation and Elaboration for important clarifications on all the items. If relevant, we also recommend reading CONSORT extensions for cluster randomised trials, non-inferiority and equivalence trials, non-pharmacological treatments, herbal interventions, and pragmatic trials. Additional extensions are forthcoming: for those and for up to date references relevant to this checklist, see www.consort-statement.org.

For peer review only - http://bmjopen.bmj.com/site/about/guidelines.xhtml

For peer review only

Web-based self-management support for people with type 2 diabetes (HeLP-Diabetes): randomised controlled trial in English primary care.

Journal:	*BMJ Open*
Manuscript ID	bmjopen-2017-016009.R1
Article Type:	Research
Date Submitted by the Author:	17-May-2017

Complete List of Authors:	Murray, Elizabeth; University College London, Research Department of Primary Care and Population Health<br>Sweeting, Michael; MRC Biostatistics Unit, Cambridge,<br>Dack, Charlotte; University of Bath, Department of Psychology<br>Pal, Kingshuk; University College London, Department of Primary Care & Population Health<br>Modrow, Kerstin; University College London, Research Department of Primary Care and Population Health<br>Hudda, Mohammed; MRC Biostatistics Unit, Cambridge,<br>Li, Jinshuo; University of York, Department of Health Sciences<br>Ross, Jamie; University College London, Primary Care & Population Health<br>Alkhaldi, Ghadah; University College London, Research Department of Primary Care and Population Health<br>Barnard, Maria; Whittington Health NHS Trust<br>Farmer, Andrew; University of Oxford, Department of Primary Care Health Sciences<br>Michie, Susan; University College London, Centre for Outcomes Research and Effectivenes<br>Yardley, Lucy; University of Southampton, Academic Unit of Psychology<br>May, Carl; University of Southampton, Faculty of Health Sciences<br>Parrott, Steve; University of York, Department of Health Sciences<br>Stevenson, Fiona; UCL, Primary care and population sciences<br>Knox, Malcolm; University College London, Research Department of Primary Care and Population Health<br>Patterson, David; Whittington Health NHS Trust

**Primary Subject Heading**:	Diabetes and endocrinology
Secondary Subject Heading:	General practice / Family practice, Health informatics, Health services research, Patient-centred medicine
Keywords:	internet, self care, Diabetes mellitus, type 2, PRIMARY CARE

SCHOLARONE™ Manuscripts

For peer review only

Title: Web-based self-management support for people with type 2 diabetes (HeLP-Diabetes): randomised controlled trial in English primary care.

Authors:

Elizabeth Murray[1], Professor of eHealth and Primary Care
Michael Sweeting[2], Senior Research Associate
Charlotte Dack[3], Lecturer,
Kingshuk Pal[1], Clinical Research Associate
Kerstin Modrow[1], Data Manager
Mohammed Hudda[4], Research Fellow in Medical Statistics
Jinshuo Li[5], Research Fellow
Jamie Ross[1], Research Associate
Ghadah Alkhaldi[1] PhD student
Maria Barnard[6], Consultant in Diabetes and Endocrinology,
Andrew Farmer[7], Professor of General Practice
Susan Michie[8], Professor of Health Psychology
Lucy Yardley[7,9], Professor of Health Psychology
Carl May[10], Professor of Health Care Innovation
Steve Parrott[5] Reader in Health Economics,
Fiona Stevenson[1] Reader in Medical Sociology
Malcolm Knox[1] Patient Representative
David Patterson[6], Consultant Cardiologist

[1]Research Department of Primary Care and Population Health, University College London, London NW3 2PF, UK
[2]Cardiovascular Epidemiology Unit, Department of Public Health and Primary Care, University of Cambridge, Worts Causeway, Cambridge, CB1 8RN
[3]Department of Psychology, University of Bath, Claverton Down, Bath, BA2 7AY
[4]Population Health Research Institute, St George's, University of London, Cranmer Terrace, London SW17 0RE
[5]Department of Health Sciences, University of York. Seebohm Rowntree Building, University of York, Heslington, York, YO10 5DD, UK
[6]Whittington Health, Magdala Avenue, London N19 5NF
[7]Nuffield Department of Primary Care Health Sciences, University of Oxford, Radcliffe Observatory Quarter, Woodstock Road, Oxford. OX2 6GG
[8]Centre for Behaviour Change, Research Department of Clinical, Educational and Health Psychology, University College London, 1-19 Torrington Place, London, WC1E 7HB
[9]Department of Psychology, Building 44 Highfield Campus, University of Southampton SO17 1BJ
[10]Health Sciences, University of Southampton, University Road, Southampton SO17 1BJ

Author for correspondence:
Professor Elizabeth Murray, Research Department of Primary Care and Population Health, University College London, London NW3 2PF, UK. Elizabeth.murray@ucl.ac.uk

For peer review only - http://bmjopen.bmj.com/site/about/guidelines.xhtml

Abstract

Objective: To determine the effectiveness of a web-based self-management programme for people with type 2 diabetes in improving glycaemic control and reducing diabetes-related distress.

Methods

Design: Individually randomised two-arm controlled trial

Setting: 21 General Practices in England

Participants: Adults aged 18 or over with a diagnosis of type 2 diabetes registered with participating general practices

Intervention and comparator: Usual care plus ***either*** HeLP-Diabetes, an interactive, theoretically informed, web-based self-management programme ***or*** a simple, text-based website containing basic information only.

Outcomes and data collection: Joint primary outcomes were glycated haemoglobin (HbA1c) and diabetes-related distress, measured by the Problem Areas in Diabetes (PAID) scale, collected at 3 and 12 months after randomisation, with 12 months the primary outcome point. Research nurses, blind to allocation collected clinical data; participants completed self-report questionnaires online.

Analysis: The analysis compared groups as randomised (intention to treat) using a linear mixed effects model, adjusted for baseline data with multiple imputation of missing values.

Results

Of the 374 participants randomised between September 2013 and December 2014, 185 were allocated to the intervention and 189 to the control. Final (12 month) follow up data for HbA1c were available for 318 (85%) and for PAID 337 (90%) of participants. Of these, 291 (78%) and 321 (86%) responses were recorded within the pre-defined “window” of 10-14 months. Participants in the intervention group had lower HbA1c than those in the control (mean difference -0.24%; 95% Confidence Interval -0.44 to -0.049; p=0.014). There was no significant overall difference between groups in the mean PAID score (p=0.21), but pre-specified subgroup analysis of participants who had had diabetes for less than 7 years showed a beneficial impact of the intervention in this group (p = 0.004). There were no reported harms.

Conclusions

Access to HeLP-Diabetes improved glycaemic control over 12 months.

Registration

Trial registration ISRCTN02123133.

Word count of abstract = 299.

For peer review only

For peer review only - http://bmjopen.bmj.com/site/about/guidelines.xhtml

Strengths and Limitations of this study

- The trial recruited to target and achieved reasonable follow-up; hence the results for the population of participants are robust;
- However, despite wide inclusion criteria and a deliberately pragmatic design, trial participants were well-controlled at baseline, and therefore the extent to which the trial results generalise to the wider population of people with type 2 diabetes is open to discussion.

For peer review only

For peer review only - http://bmjopen.bmj.com/site/about/guidelines.xhtml

Introduction.

There is a global epidemic of type 2 diabetes mellitus (T2DM). An estimated 422 million adults, or 10% of the global population, were living with diabetes in 2014 of whom around 90% had type 2 diabetes.[1] Poorly controlled diabetes is associated with premature mortality, and a high risk of complications, including cardiovascular disease, nephropathy and retinopathy. The risk of complications can be reduced by good control of glycaemia and cardiovascular risk factors. [2 3] Interventions which improve self-management skills for patients with diabetes can improve health outcomes and reduce health care costs[4] and international guidelines support training patients in self-management. [3 5] However, it is not clear how best to support patients in developing such skills, and uptake of diabetes self-management education remains low. In England, despite over 90% of eligible patients being referred, [6] only 5.3% attended self-management training in 2014 – 15. [7]

Poor uptake may be related to the dominant model of structured education, which is group-based sessions, lasting a half or whole day, or spread over regular sessions over several weeks. [8] Many patients, such as those who work, those with caring commitments, or those who are uncomfortable in groups, may find it difficult to attend.[9 10]

Web-based support for self-management could address some of these barriers, particularly in high income countries, where levels of web-access are high. In the UK over 80% of households had Internet access in 2015, and internet access amongst older people continues to grow steadily. [11 12] Potential advantages include convenience, anonymity, regular updates, and the potential to use video and graphics to present complex information in a format accessible to those with low literacy.[13] Although systematic reviews have confirmed that computer-based interventions can improve health outcomes in diabetes [14], not all such interventions have a beneficial impact, with meta-analyses showing substantial heterogeneity related to widely differing interventions, including in the use of theory to develop the intervention, [15] outcomes, [14 16] and the duration of follow-up, with most trials having relatively short follow-up (less than 12 months). [14] This is the first UK-based trial of a comprehensive, web-based self-management support programme for people with type 2 diabetes.

This trial assessed the effects of a web-based self-management programme, called Healthy Living for People with Diabetes (HeLP-Diabetes), on glycated haemoglobin (HbA1c) and diabetes-related distress over 12 months.

Methods.

Trial Design and participants: multi-centre, two-arm individually randomised controlled trial in 21 General Practices in England with a mix of urban, suburban and rural practices. Practices were required to have two nurses – one to facilitate access to the intervention, and one to collect data.

Recruitment: standard opt-in recruitment procedures were followed. Each practice had a register of patients with T2DM. The electronic medical record of every patient on this register was reviewed to screen out ineligible patients, and the remainder were sent a letter from their GP, inviting them to participate in the study. Eligible participants were adults, aged 18 or over, with T2DM, registered with participating general practices. Patients were excluded if they were unable to provide informed consent; unable to use a computer due to severe mental or physical impairment; had insufficient spoken or written English to use the intervention (operationalised as unable to consult without an interpreter); were terminally ill with less than 12 months life expectancy; or were currently participating in a trial of an alternative self-management programme. Participants were not required to have home

For peer review only - http://bmjopen.bmj.com/site/about/guidelines.xhtml

Internet access or prior experience of using the Internet to participate. Participants with previous or current experience of self-management education were eligible to participate. Recruitment took place between September 2013 and December 2014. The trial protocol was submitted for publication in June 2014.[17] There were no changes to the methods after the protocol was agreed and the start of the trial. Ethical approval was obtained from Camden and Islington National Research Ethics Service (NRES) committee, reference 12/LO/1571.

Patient involvement

Patients were involved in all stages of the study, including contributing to the original application for funding as co-investigators; substantive and ongoing contribution to intervention development; contributing to the trial design, including the decision to have two co-primary outcomes; active membership of the Trial Steering Committee and Trial Management Group; and contributing to the writing of this paper. This last role is recognised through co-authorship (MK).

Randomisation and blinding

Randomisation marked the point of study entry. It was performed centrally (independently of the trial team), after written informed consent was obtained and all baseline data were completed, using a web-based randomisation system, at the level of the individual participant. Randomisation was conducted in a 1:1 ratio using random permuted blocks of sizes 2, 4 and 6, stratified by recruitment centre. Participants were informed the trial compared two forms of web-based support, and were blinded as to which was the intervention and which the comparator. Nurses who offered facilitation for the intervention could not be blinded, but were asked not to discuss details of allocation with the nurses who gathered follow-up data. The research team obtaining and analysing data from participants were blind to allocation.

Intervention

The intervention consisted of facilitated access to HeLP-Diabetes. Facilitation consisted of an introductory training session with the practice nurse. In this appointment patients were were shown how to log on, set a user name and password, and introduced to the content of the website. HeLP-Diabetes was a theoretically informed web-based programme whose overall goals were to improve health outcomes and reduce diabetes-related distress.[18] Overall content was guided by the Corbin and Strauss model of managing a long term condition which posits that patients must undertake medical, emotional and role management.[19] It was developed using participatory design principles, with substantial input from users, defined as patients with T2DM and health professionals caring for such patients. All content was evidence-based, drawing on evidence on management of diabetes, promoting behaviour change and emotional wellbeing, and maximising usability and engagement. Content was designed to be accessible to people with a wide range of literacy and health literacy skills, with all essential content provided in both video and text. There were information sections on diabetes, how diabetes is treated, possible complications of diabetes, possible impacts of diabetes on relationships at home and at work, dealing with unusual situations like parties, holidays, travelling or shift work, and what lifestyle modifications will improve health. There were sections addressing skills and behaviour change, including behaviour change modules on eating healthily, losing weight, being more physically active, smoking cessation, moderating alcohol consumption, managing medicines, glycaemic control and blood pressure control. Users could set the programme to send themselves reminder text messages or emails, and could specify the content and frequency of such reminders. The third strand of components focused on emotional well-being with self-help tools based on cognitive behavioural therapy and mindfulness. There were multiple personal stories (used with license from health talk online), and a moderated forum. Participants were free to use the programme as much or as little as they chose. Engagement with the programme was promoted through regular newsletters, emails and SMS containing

For peer review only - http://bmjopen.bmj.com/site/about/guidelines.xhtml

updates on latest diabetes-related research or practice, seasonally-relevant advice (e.g. fasting during Ramadan, benefits of 'flu vaccinations), and links to specific relevant parts of the programme. Two or three prompts were sent each month, although users could opt out of receiving them. Further details are provided in Appendix 1.

Comparator
From an NHS perspective, the important research question was whether the proposed intervention could improve health outcomes when compared to current practice. However, to improve acceptability to participants and to maintain blinding, all participants had access to a website. Participants in the control arm were given access to a simple information website, based on the information available on the website of the main UK diabetes charity (Diabetes UK) or National Health Service patient information website (NHS Choices). They received the same initial facilitation meeting as participants in the intervention group, in which they were shown how to log on, set a user name and password, and how to use the website.

Outcomes and outcome measures

Primary outcomes
The outcomes reflected the dual goals of improving health outcomes and reducing diabetes-related distress. The two joint primary outcomes were glycated haemoglobin (HbA1c) and diabetes-related distress, measured by the Problem Areas in Diabetes (PAID) scale, both at 12 months post-randomisation. PAID has 20 items focusing on areas that cause difficulty for people living with diabetes, including social situations, food, friends and family, diabetes treatment, relationships with health care professionals and social support.[20] PAID scores range from 0 – 100, with higher scores indicating more distress. A score of 40 or more indicates significant distress, and around 40% of patients with diabetes experience significant distress.[21]

Secondary outcomes
Clinical secondary outcomes included systolic and diastolic blood pressure; body mass index; total cholesterol and HDL (not fasting); and completion of the "9 essential processes" for effective management of diabetes, mandated by NHS England (= weight, BP, smoking status, measurement of serum creatinine, cholesterol and HbA1c, urinary albumin and assessment of eyes and feet) within the previous 12 months.[3] Patient-reported outcomes included depression and anxiety, measured using the Hospital Anxiety and Depression Scale (HADS);[22] diabetes-related self-efficacy measured using the Diabetes Management Self-Efficacy Scale (DMSES);[23] and satisfaction with treatment, measured using the Diabetes Satisfaction with Treatment Questionnaire status and change version (DTSQs & DTSQc).[24]

Data collection
Data were collected at baseline, 3 and 12 months, with 12 months the primary endpoint. Patient-reported data were collected using online questionnaires emailed to participants. Clinical outcomes were collected by nurses in participating practices. Participants were asked to complete their online questionnaires before visiting the nurse for clinical measurements and blood tests. Blood samples were analysed at the local NHS laboratory used by participating practices for routine clinical analyses. Data on completion of the "9 essential processes" were collected from the GP record for the 12 months prior to randomisation and the 12 months after randomisation at the 12 month follow-up point to avoid triggering behaviour change amongst the study nurses. Use of the intervention was recorded automatically using bespoke software that recorded the date, and time of each

For peer review only - http://bmjopen.bmj.com/site/about/guidelines.xhtml

For peer review only

page visited. A new log-in to the intervention was defined as any page that was accessed 30 minutes or more after the last accessed page.

Sample size Calculation
Our original sample size calculation was that randomising 350 participants with 85% follow-up would provide 90% power at the 5% level of significance to detect a 0.25% difference in HbA1c and a 4.0 point difference in PAID score at 12 months post-randomisation between the randomised groups.[25 26] Since HbA1c and PAID were joint primary outcomes measuring different aspects of T2DM, both were tested at a 5% significance level.

Analysis
The analysis followed a pre-specified analysis plan, based on comparing the groups as randomised (intention-to-treat). The analysis plan was approved by the Trial Steering Committee before unblinding and uploaded to the trial website, https://www.ucl.ac.uk/pcph/research-groups-themes/ehealth/projects/projects/helpdiabetesrct. Only HbA1c and PAID measured within a 10-14 month window period following randomisation was used in the primary analysis with missing 12-month outcomes multiply imputed using baseline and other outcome data (e.g. 3m data and final follow-up data collected outside the 10-14 month window). Further information on the imputation method is given in Appendix 2.

A linear mixed effects model with random centre effects was used to analyse each of the primary outcomes separately, adjusting for the baseline level of the outcome, age, gender, previous participation in other self-management programmes, pre-existing cardiovascular disease and time since diagnosis of diabetes. Secondary outcome measures were analysed similarly using generalised linear mixed models, with a normal residual error structure for continuous outcomes and a logit link for the binary outcome `Completion of 9 essential processes'. Pre-specified sub-group analysis for the co-primary outcomes was undertaken by baseline glycaemic control (HbA1c outcome only), baseline PAID (PAID outcome only), and duration of diabetes, treating all potential effect modifiers as continuous. The interaction between randomised group and each effect modifier was included in the model separately and assessed using a Wald test.

Use of the intervention was investigated as a mediator for efficacy, using instrumental variable methods, with randomisation as the instrument (Supplementary Figure 1).[27 28]

Potential contamination was monitored by recording participants with similar family names and identifying those with the same addresses. Where this occurred, it was dealt with in the analysis by reporting the extent and undertaking a sensitivity analysis excluding these individuals.

A number of other sensitivity analyses were performed to assess the robustness of the primary analyses: 1) performing two complete case analyses disregarding outcomes measured outside 10-14 months and 11-13 months post-randomisation; 2) repeating the analysis using multiple imputation of baseline covariates only; 3) fitting linear models excluding centre random-effects; and 4) fitting an unadjusted model using only outcome measured in 10-14 months post-randomisation.

The TSC took on the role of the data monitoring committee. Trial registration ISRCTN02123133.

Results

For peer review only - http://bmjopen.bmj.com/site/about/guidelines.xhtml

Recruitment took place between September 2013 and December 2014. An initial 421 patients consented to participate, but of these 47 did not fully complete their baseline questionnaires and were therefore not randomised and did not enter the study. A total of 374 participants were randomised, of whom 86% (n = 321) provided data on PAID, and 78% (n = 291) had HbA1c measured within 10 to 14 months of randomisation. Additional final outcome data, obtained outside the 10 – 14 month pre-defined window, were available for a further 27 participants for HbA1c and 16 participants for PAID (Figure 1). Data obtained outside the 10 – 14 month window were not used directly in the primary analysis, but were entered into the imputation model (Supplementary Table 1).

Baseline characteristics

Baseline demographic and clinical characteristics are shown in Table 1. The mean age was nearly 65 years, over two-thirds (n = 258, 69%) were male, and most were White British (n = 300, 80%). Nearly all (n=370, 99%) had a computer with access to the internet at home and just over half (n = 210, 56%) rated themselves as experienced computer users. Around one-third (n = 134; 36%) had been diagnosed for less than 5 years, with a further third (n = 115, 31%) having been diagnosed between 5 and 9 years ago. Overall, this was a population with well-controlled diabetes at baseline (mean HbA1c was 7.3% (56 mmol / mol)) and low levels of distress (mean PAID = 19).

Primary outcomes

At twelve months the primary analysis showed a significant difference in change in HbA1c between the randomised groups with participants in the HeLP-Diabetes group having a lower HbA1c than those in the control group (mean difference = -0.24%; 95% confidence intervals -0.44 to -0.049, p=0.014) (Table 2, Figure 2). There was no difference in change in PAID scores between the groups at 12-months (mean difference -1.5; 95% CI -3.9, 0.9, p=0.209), though both groups showed a decrease in PAID over the follow-up of the trial (Table 2, Figure 3).

Secondary outcomes

There was no difference in secondary outcomes at 12 months, with the possible exception of systolic blood pressure, which decreased more in the intervention group than in the control group (p=0.010) (Table 2); though the result was not statistically significant after correction for multiple testing of secondary outcomes. There were no significant differences between groups on any of the outcome measures amongst individuals who completed three month outcomes (Supplementary Table 2). No adverse effects or events were recorded during follow-up.

Usage data

The mean number of log-ins was significantly higher in the intervention group than the control group (18.7 vs. 4.8, p= 0.0001), as was the mean number of pages visited per log-in (10.5 vs. 7.7, p <0.0001) and the mean number of days in which the website was accessed (10.1 vs. 3.3, p<0.0001) (Table 3). The causal analyses estimated that for a "high-usage" population (those with usage ≥ the median of 4 days) the HeLP-Diabetes intervention could on average reduce HbA1c by -0.44% (95% CI -0.81 to -0.06) and PAID by -2.8 (95% CI -7.2 to 1.7) over 12 months (Supplementary Figures 2 and 3). The mean usage in the "high-usage" group was 18 days. It should be noted that the usage data presented do not include the initial facilitation visit. There was a technical error in the software which led to usage data not being collected before 1 January 2014. At this point 16 participants had been randomised (7 to intervention, 9 to control). For these 16 participants, the usage data is not based on a full year, but for all other participants, data are summarised for the 12 months post-randomisation.

Sensitivity analyses

For peer review only - http://bmjopen.bmj.com/site/about/guidelines.xhtml

The findings from the sensitivity analyses, including a complete-case analysis, were similar to the main analysis (Supplementary Table 3). Participants who were missing 12-month HbA1c had significantly higher mean baseline HbA1c measures (7.9% vs. 7.1%, $p<0.001$) leading to higher imputed HbA1c at 12-months in the non-completers and a greater mean difference between the randomised groups than from complete case analyses (Supplementary Figure 4, Supplementary Table 3).

Subgroup analyses

Pre-specified subgroup analyses showed that there was no evidence of baseline measures of HbA1c or PAID being effect modifiers for the mean difference between the groups. There was strong statistical evidence (interaction $p=0.004$) to suggest that the duration of diabetes acted as an effect modifier, with those who had been diagnosed more recently (<7 years) showing more of a reduction in PAID than those who had been diagnosed for longer periods of time. Duration of diabetes had no effect on change in HbA1c (Supplementary Table 4).

Harms

There were no reported harms in either group.

Discussion

In this first UK-based trial of a web-based self-management programme for people with T2DM, participants randomised to HeLP-Diabetes demonstrated improved glycaemic control at 12 months compared to those randomised to a simple information website. This improvement is both clinically and statistically meaningful, appears robust across all pre-specified sensitivity analyses, and was not dependent on duration of diabetes, baseline glycaemic levels or level of diabetes-related distress. Each 1% reduction in HbA1c is associated with a risk reduction of 21% for deaths related to diabetes and a 37% risk reduction for microvascular complications.[26] Given that this web-based intervention could be delivered at low-cost and at scale across the UK, the potential for population benefit is considerable. Moreover, in contrast to group-based education, where the effects appear to wane with time,[29] the effects of HeLP-Diabetes were greater at 12 months than at 3 months. There was no overall impact on diabetes-related distress, but some evidence that HeLP-diabetes appeared to reduce distress in recently diagnosed individuals. However, it is worth noting that baseline PAID scores were exceptionally low in this trial population. In a small pilot study, participants offered supported access to HeLP-Diabetes reduced their PAID scores by 6 points ($p=0.04$) over 6 weeks.[30]

The trial has many strengths. It was a pragmatic trial, open to nearly all patients with T2DM in participating practices. Concealment of allocation was complete, as randomisation occurred after baseline data collection. Baseline prognostic factors were well balanced between groups. Every effort was made to achieve blinding, including requiring practices to have two nurses, so that data collection was undertaken by a nurse blind to participant allocation. Data for the co-primary outcomes at the primary outcome point were available for 78% and 86% of participants for HbA1c and PAID respectively. All analyses were on an intention-to-treat basis, supplemented by a CACE analysis. Although response rates for the co-primary outcomes were good, some potential for bias existed. Our primary analysis used multiple imputation methods because evidence shows that the assumptions underpinning this method are more defensible than those assumed using other approaches to missing data.[31] We also undertook sensitivity analyses including complete cases, non-contaminated cases, and a linear model excluding centre; all yielded similar results.

The two co-primary outcomes reflected the twin aims of the intervention: to improve diabetes control and to reduce diabetes-related distress. Around 40% of patients with diabetes have significant levels of distress, which severely impacts on quality of life,[32] and diabetes-related distress is an important outcome for patients.[33] Our patient and public involvement (PPI)

For peer review only - http://bmjopen.bmj.com/site/about/guidelines.xhtml

panel were clear that this should be a primary outcome, and a recent meta-ethnography emphasised the importance of empowerment and quality of life in promoting long term engagement with self-management.[34] In contrast, many health care professionals are more interested in glycaemic control. In line with previous trials in this area,[35] we decided to adopt both as co-primary outcomes and to test both at a 5% level of significance.[36]

There are some limitations. Despite maximising the inclusivity of the trial by minimising the exclusion criteria, participants were not representative of the overall population of patients with type 2 diabetes in England. Compared to the overall population, participants had better control of their diabetes and cardiovascular risk factors, [6 7] and were much less distressed. [21] This finding mirrors that of a recent systematic review of demographic factors associated with web portal usage amongst people with diabetes which found that those with well controlled diabetes were more likely to use such portals than those with poor control. [37] However, fewer of our participants self-rated their computer skills as excellent (57% of our sample compared to a national average of 73%).[12] This good control at baseline has two implications – first, that there was little room for improvement in this population, and secondly, that this population may have been unusually motivated to self-manage their diabetes. Although every effort was made to maintain blinding, it is possible that some participants may have discussed their use of the intervention with research nurses, making it possible to infer which arm they had been allocated to. This could have affected research nurses' measurements of secondary clinical outcomes, such as blood pressure or weight, but could not have affected assessment of glycated haemoglobin as this was measured by laboratory staff who were blinded. There appeared to be high potential for contamination between two participants who shared the same surname and address, and a further two participants did not receive their allocated intervention due to an error at practice level; excluding these four made no difference to the results. A further limitation of the trial is that it provides little insight into the mechanism of action of HeLP-Diabetes. This was the result of a deliberate decision to focus on clinically important outcomes and minimise both the response burden and the potential impact of measurement on participants.

This is the first UK-based trial of a web-based self-management programme for people with type 2 diabetes, and internationally, the first trial of such a comprehensive intervention that aims to address the three main tasks of self-management: emotional, medical and role management.[19] In the Cochrane review of computer-based self-management interventions for people with T2DM, only 4 of the included studies had follow-up of 12 months or more.[14] Of these, three interventions were clinic-based, with participants completing self-assessment tools on a touch screen and receiving tailored advice during their baseline visit to their diabetes clinician [38-40] and one was a mobile phone-based intervention which provided tailored messages in response to participant's results of blood glucose self-monitoring data. [41] A more recent systematic review of internet delivered diabetes self-management identified 2 trials with 12 or more months follow-up. [42] One trial was on a structured intervention based on a peer-led, group-based, diabetes self-management course [43]. There were six sessions, with each session available for one week. Each session required participants to make a specific action plan to address a problem they were experiencing. Peer facilitators encouraged use of the programme. Follow up was planned at 6 and 12 months; however HbA1c data were only available at 6 months. The other trial compared two versions of a web-based intervention (with and without additional social support) to enhanced usual care. The web-based intervention was designed using social-cognitive theory and a social-ecological model, with a focus on three main behaviours: dietary intake, physical activity and medication adherence. Users of either web-based intervention received motivational phone calls to encourage adherence and development of action plans. Those randomised to the enhanced intervention (with additional social support) received two additional phone calls and an invitation to attend a group session. There was no difference between groups in HbA1c or other biological outcomes at 12 months. [44] Thus the results of this trial add significantly to the available literature.

For peer review only - http://bmjopen.bmj.com/site/about/guidelines.xhtml

On the basis of these results, HeLP-Diabetes may be considered as an addition to the current menu of self-management support for people with type 2 diabetes, and may help increase overall access and uptake. Most commissioned services currently focus on newly diagnosed patients, leaving clear unmet need for people who have had their diabetes for longer, but are looking for ways to improve their health. Many patients are not ready to engage in self-management early in their illness journey,[9] but become motivated to do so later, often as a result of a change in medication or development of a complication.[45] The intervention is low cost, and as most costs are fixed, irrespective of number of users, is likely to be cost-effective, particularly if widely used. A cost-effectiveness analysis of HeLP-Diabetes will be reported separately.

Word count 4,303

Contributors

EM, MS, CD, KP, MB, AF, SM, LY, CM, SP, FS and DP all contributed to the design of the trial. MS, assisted by MH and KM designed the statistical analysis plan and undertook the analysis. SP and JL designed and undertook the health economic aspects of the trial. EM, KP, CD, JR, GA, LY and SM contributed to the development and delivery of the intervention. MK contributed PPI input. EM and MS wrote the first draft of the paper; all authors commented on this draft and have approved the final version.

Declaration of interests

EM is the Managing Director of a not-for-profit Community Interest Company established to disseminate HeLP-Diabetes across the UK.

Acknowledgements

Funding.

This paper presents independent research funded by the National Institute for Health Research (NIHR) under its Programme Grants for Applied Research Programme (Grant Reference Number RP-PG-0609-10135). The views expressed are those of the author(s) and not necessarily those of the NHS, the NIHR or the Department of Health. The funder had no role in the study design, data collection, data analysis, data interpretation, or writing of the report. All authors had full access to all the data in the study and can take responsibility for the integrity of the data and data analysis. The lead author had final responsibility for the decision to submit for publication, affirms that the manuscript is an honest, accurate, and transparent account of the study being reported; that no important aspects of the study have been omitted; and that there were no significant discrepancies from the published protocol.

Work conducted at the Cardiovascular Epidemiology Unit, University of Cambridge by MS and MH was additionally funded by the UK Medical Research Council (MR/L003120/1), British Heart Foundation (RG/13/13/30194) and UK National Institute for Health Research Cambridge Biomedical Research Centre.

For peer review only - http://bmjopen.bmj.com/site/about/guidelines.xhtml

AF is an NIHR Senior Investigator and receives funding from Oxford NIHR Biomedical Research Centre

We gratefully acknowledge the permission to use under license the validated behaviour change modules for weight loss (POWeR), alcohol reduction (DownYour Drink) and smoking cessation (StopAdvisor and the diabetes module from Healthtalk online (http://www.healthtalk.org/) in HeLP-Diabetes.

We are grateful to Orla O'Donnell for outstanding project management, Fiona Giles for administrative support, all our PPI who contributed to the development, maintenance and delivery of the intervention and / or the management and oversight of the trial, staff at the participating practices, PCRN staff, and all our participants.

Data Sharing.

Patient level data, the full dataset, statistical code are available from the corresponding author. Consent for data sharing was not obtained from participants, but the potential benefits of sharing these data outweigh the potential harms as the data are anonymised.

For peer review only

For peer review only

For peer review only - http://bmjopen.bmj.com/site/about/guidelines.xhtml

Table 1: Descriptive statistics of baseline variables by randomised group. Mean (SD) unless otherwise specified.

	HeLP-Diabetes N=185	Control N=189	N missing
Age at randomisation (yrs)	64.9 (9.5)	64.7 (9.1)	0
Male sex, n (%)	127 (69%)	131 (69%)	0
Ethnicity, n(%):			1
White English, Welsh, Scottish, Northern Irish, British	151 (82%)	149 (79%)	
Indian	12 (6%)	8 (4%)	
Other	21 (11%)	31 (16%)	
Experience with computers, n(%)			0
None	5 (3%)	4 (2%)	
Basic	75 (41%)	80 (42%)	
Experienced	105 (57%)	105 (56%)	
Smoking status, n(%):			0
Current smoker	14 (8%)	14 (7%)	
Former smoker	94 (51%)	86 (46%)	
Never smoker	77 (42%)	89 (47%)	
Time since diagnosis (years), n(%):			4
0-4 years	70 (38%)	64 (34%)	
5-9 years	55 (30%)	60 (32%)	
10-14 years	40 (22%)	40 (21%)	
15+ years	18 (10%)	23 (12%)	
Attending any other self-management class, n(%)	4 (2%)	4 (2%)	0
**Clinical Measures**			
Systolic blood pressure (mmHg)	135 (17)	135 (17)	0
Diastolic blood pressure (mmHg)	78 (11)	77 (10)	0
Total cholesterol (mmol/l)	4.11 (1.03)	4.18 (0.98)	2
HDL-C (mmol/l)	1.24 (0.31)	1.25 (0.36)	12
Total cholesterol /HDL-C ratio	3.43 (1.09)	3.52 (1.03)	13
HbA1c (%)	7.26 (1.25)	7.35 (1.37)	5
HbA1c (mmol/mol)	56 (14)	57 (15)	5
Body mass index (Kg/m^2)	30.1 (5.3)	29.6 (5.2)	2
**Questionnaires / scores**			
PAID (0-100)	18.1 (17.1)	19.9 (19.9)	0
HADS (0-42)	9.28 (6.47)	9.12 (7.52)	0
Anxiety scale (0-21)	4.92 (3.70)	5.21 (4.20)	0
Depression scale (0-21)	4.36 (3.48)	3.91 (3.73)	0
DMSES (0-150)	98.6 (33.9)	103.7 (32.4)	0
DTSQ (0-48)	32.1 (7.3)	32.0 (7.2)	0
Completion of 9 essential processes in previous 12 months, n(%)	97 (64%)	96 (62%)	69

For peer review only

For peer review only - http://bmjopen.bmj.com/site/about/guidelines.xhtml

Table 2: 12 month outcomes, adjusted for relevant baseline outcome, age, sex, current (baseline) participation in other self-management programmes, pre-existing cardiovascular disease and duration of diabetes.
Results from multiply imputed data shown. Data are mean (SE) or mean difference (95% CI) unless otherwise specified.

	HeLP-Diabetes		Control		HeLP-Diabetes vs Control	
	Baseline	Change from baseline to 12 months	Baseline	Change from baseline to 12 months	Mean difference (95% CI)	p-value
**Primary outcomes**						
HbA1c, (%)	7.3 (0.1)	-0.08 (0.07)	7.3 (0.1)	0.16 (0.07)	-0.24 (-0.44, -0.05)	0.014
HbA1c, mmol/mol	56.3 (1.1)	-0.8 (0.8)	56.8 (1.1)	1.8 (0.8)	-2.6 (-4.8, -0.5)	0.014
PAID	18.2 (1.3)	-4.1 (0.9)	19.8 (1.3)	-2.5 (0.9)	-1.5 (-3.9, 0.9)	0.209
**Secondary outcomes**						
Systolic blood pressure, mmHg	134.7 (1.5)	-4.2 (1.4)	134.9 (1.5)	-0.5 (1.4)	-3.8 (-6.6, -0.9)	0.010
Diastolic blood pressure, mmHg	77.8 (1.0)	-2.5 (0.9)	77.1 (1.0)	-1.9 (0.8)	-0.6 (-2.4, 1.2)	0.519
Body mass index, Kg/m^2	30.1 (0.5)	0.12 (0.2)	30.0 (0.5)	-0.04 (0.2)	0.16 (-0.30, 0.62)	0.498
Total cholesterol, mmol/L	4.1 (0.1)	-0.08 (0.06)	4.2 (0.1)	-0.15 (0.06)	0.07 (-0.09, 0.2)	0.370
HDL cholesterol, mmol/L	1.25 (0.03)	-0.003 (0.018)	1.26 (0.03)	0.004 (0.018)	-0.007 (-0.054, 0.039)	0.754
Completion of 9 essential processes*	65% (3.7)	-5.1%	61% (3.8)	3.4%	0.78 (0.45, 1.35)	0.379
HADS	9.3 (0.5)	-1.05 (0.44)	9.1 (0.5)	-0.60 (0.48)	-0.45 (-1.68, 0.78)	0.474
DMSES**	98.8 (2.4)	2.93 (2.90)	103.6 (2.3)	1.38 (2.79)	1.55 (-5.74, 8.84)	0.674
DTSQ	32.2 (0.6)	0.94 (0.57)	32.2 (0.6)	0.45 (0.61)	0.49 (-1.18, 2.15)	0.564

* Percentage (SE) and odds ratio (95% CI)
** linear regression results shown due to lack of convergence for mixed model

For peer review only - http://bmjopen.bmj.com/site/about/guidelines.xhtml

Table 3. Extent of website usage over 12-month follow-up.

	HeLP-Diabetes	Control	p-value*	N missing
Number of log-ins per person	18.7 (84.0)	4.8 (8.0)	0.0001	0
Number pages visited per log-in	10.5 (6.7)	7.7 (5.0)	<0.0001	105**
Time spent in each log-in (minutes)***	12.3 (9.8)	8.2 (8.4)	<0.0001	105**
Number of days in which website was accessed over follow-up	10.1 (22.9)	3.3 (5.1)	<0.0001	0

Mean (SD) unless otherwise specified.
* Wilcoxon rank-sum test.
** 105 individuals did not log in after their facilitation visit (42 intervention, 63 control).
*** Measured as time from first page accessed to last page accessed within a log-in session.

For peer review only

Reference List

1. Worldwide trends in diabetes since 1980: a pooled analysis of 751 population-based studies with 4.4 million participants. Lancet (London, England) 2016;**387**(10027):1513-30.
2. Tkac I. Effect of intensive glycemic control on cardiovascular outcomes and all-cause mortality in type 2 diabetes: Overview and metaanalysis of five trials. Diabetes Res Clin Pract 2009;**86 Suppl 1:S57-62.**:S57-S62.
3. National Institute of Health and Care Excellence (NICE). Type 2 diabetes in adults: Management. NG28. London, 2015.
4. Panagioti M, Richardson G, Murray E, et al. Self-management support interventions to reduce health care utilisation without compromising outcomes: a systematic review and meta-analysis. BMCHealth ServRes 2014;**14:356. doi: 10.1186/1472-6963-14-356.**:356-14.
5. (4) Foundations of care: education, nutrition, physical activity, smoking cessation, psychosocial care, and immunization. Diabetes care 2015;**38 Suppl**:S20-30.
6. NHS Digital. Quality and Outcomes Framework (QOF) - 2014-15. October 29 2015 ed. London: NHS Digital, 2015.
7. National Diabetes Audit 2013-2014 and 2014-2015 Report 1: Care Processes and Treatment Targets, 2016:1-35.
8. Deakin T, McShane CE, Cade JE, et al. Group based training for self-management strategies in people with type 2 diabetes mellitus. Cochrane Database SystRev 2005;**18**(2):CD003417.
9. Winkley K, Evwierhoma C, Amiel SA, et al. Patient explanations for non-attendance at structured diabetes education sessions for newly diagnosed Type 2 diabetes: a qualitative study. Diabetic medicine : a journal of the British Diabetic Association 2015;**32**(1):120-8.
10. Horigan G, Davies M, Findlay-White F, et al. Reasons why patients referred to diabetes education programmes choose not to attend: a systematic review. Diabetic medicine : a journal of the British Diabetic Association 2017;**34**(1):14-26.
11. Internet Access - Households and Individuals: 2015: Office for National Statistics, 2015:1-14.
12. Dutton WH, Blank G, Groselj D. Cultures of the Internet: The Internet in Britain. Oxford Internet Survery 2013. Oxford: Oxford Internet Institute, 2013:1 - 64.
13. Rowsell A, Muller I, Murray E, et al. Views of People With High and Low Levels of Health Literacy About a Digital Intervention to Promote Physical Activity for Diabetes: A Qualitative Study in Five Countries. Journal of medical Internet research 2015;**17**(10):e230.
14. Pal K, Eastwood SV, Michie S, et al. Computer-based diabetes self-management interventions for adults with type 2 diabetes mellitus. CochraneDatabaseSystRev 2013;**3:CD008776. doi: 10.1002/14651858.CD008776.pub2.**:CD008776.
15. van Vugt M, de Wit M, Cleijne WH, et al. Use of behavioral change techniques in web-based self-management programs for type 2 diabetes patients: systematic review. Journal of medical Internet research 2013;**15**(12):e279.
16. Hadjiconstantinou M, Byrne J, Bodicoat DH, et al. Do Web-Based Interventions Improve Well-Being in Type 2 Diabetes? A Systematic Review and Meta-Analysis. Journal of medical Internet research 2016;**18**(10):e270.
17. Murray E, Dack C, Barnard M, et al. HeLP-Diabetes: randomised controlled trial protocol. BMC health services research 2015;**15**:578.
18. Integrating theory, qualitative data and participatory design to develop HeLP-Diabetes: an internet self-management intervention for people with type 2 diabetes 2013/05/18/; Chicago, USA. International Society for Research on Internet Interventions.
19. Corbin JM, Strauss A. *Unending Work and Care*. First ed. San Francisco: Jossey-Bass Inc, 1988.
20. Polonsky WH, Anderson BJ, Lohrer PA, et al. Assessment of diabetes-related distress. Diabetes Care 1995;**18**(6):754-60.

For peer review only

21. Nicolucci A, Kovacs Burns K, Holt RI, et al. Diabetes Attitudes, Wishes and Needs second study (DAWN2): cross-national benchmarking of diabetes-related psychosocial outcomes for people with diabetes. Diabetic medicine : a journal of the British Diabetic Association 2013;**30**(7):767-77.
22. Zigmond AS, Snaith RP. The hospital anxiety and depression scale. Acta PsychiatrScand 1983;**67**(6):361-70.
23. Bijl JV, Poelgeest-Eeltink AV, Shortridge-Baggett L. The psychometric properties of the diabetes management self-efficacy scale for patients with type 2 diabetes mellitus. JAdvNurs 1999;**30**(2):352-59.
24. Bradley C, Lewis KS. Measures of psychological well-being and treatment satisfaction developed from the responses of people with tablet-treated diabetes. DiabetMed 1990;**7**(5):445-51.
25. Welch GW, Jacobson AM, Polonsky WH. The Problem Areas in Diabetes Scale. An evaluation of its clinical utility. Diabetes care 1997;**20**(5):760-6.
26. Stratton IM, Adler AI, Neil HA, et al. Association of glycaemia with macrovascular and microvascular complications of type 2 diabetes (UKPDS 35): prospective observational study. BMJ (Clinical research ed) 2000;**321**(7258):405-12.
27. White IR, Kalaitzaki E, Thompson SG. Allowing for missing outcome data and incomplete uptake of randomised interventions, with application to an Internet-based alcohol trial. Statistics in medicine 2011;**30**(27):3192-207.
28. Dunn G, Maracy M, Tomenson B. Estimating treatment effects from randomized clinical trials with noncompliance and loss to follow-up: the role of instrumental variable methods. StatMethods Med Res 2005;**14**(4):369-95.
29. Khunti K, Gray LJ, Skinner T, et al. Effectiveness of a diabetes education and self management programme (DESMOND) for people with newly diagnosed type 2 diabetes mellitus: three year follow-up of a cluster randomised controlled trial in primary care. BMJ 2012;**344:e2333. doi: 10.1136/bmj.e2333.**:e2333.
30. Hofmann M, Dack C, Barker C, et al. The Impact of an Internet-Based Self-Management Intervention (HeLP-Diabetes) on the Psychological Well-Being of Adults with Type 2 Diabetes: A Mixed-Method Cohort Study. Journal of diabetes research 2016;**2016**:1476384.
31. Molenberghs G, Thijs H, Jansen I, et al. Analyzing incomplete longitudinal clinical trial data. Biostatistics (Oxford, England) 2004;**5**(3):445-64.
32. Chew BH, Mohd-Sidik S, Shariff-Ghazali S. Negative effects of diabetes-related distress on health-related quality of life: an evaluation among the adult patients with type 2 diabetes mellitus in three primary healthcare clinics in Malaysia. Health and quality of life outcomes 2015;**13**:187.
33. Glasgow RE, Peeples M, Skovlund SE. Where is the patient in diabetes performance measures? The case for including patient-centered and self-management measures. Diabetes Care 2008;**31**(5):1046-50.
34. Frost J, Garside R, Cooper C, et al. A qualitative synthesis of diabetes self-management strategies for long term medical outcomes and quality of life in the UK. BMC health services research 2014;**14**:348.
35. Kinmonth AL, Woodcock A, Griffin S, et al. Randomised controlled trial of patient centred care of diabetes in general practice: impact on current wellbeing and future disease risk. The Diabetes Care From Diagnosis Research Team. BMJ (Clinical research ed) 1998;**317**(7167):1202-8.
36. Schulz KF, Grimes DA. Multiplicity in randomised trials I: endpoints and treatments. Lancet (London, England) 2005;**365**(9470):1591-5.
37. Amante DJ, Hogan TP, Pagoto SL, et al. A systematic review of electronic portal usage among patients with diabetes. Diabetes technology & therapeutics 2014;**16**(11):784-93.

For peer review only - http://bmjopen.bmj.com/site/about/guidelines.xhtml

38. Christian JG, Bessesen DH, Byers TE, et al. Clinic-based support to help overweight patients with type 2 diabetes increase physical activity and lose weight. Arch Intern Med 2008;**168**(2):141-6.
39. Glasgow RE, La Chance PA, Toobert DJ, et al. Long term effects and costs of brief behavioural dietary intervention for patients with diabetes delivered from the medical office. Patient education and counseling 1997;**32**(3):175-84.
40. Glasgow RE, Nutting PA, King DK, et al. Randomized effectiveness trial of a computer-assisted intervention to improve diabetes care. Diabetes care 2005;**28**(1):33-9.
41. Quinn CC, Shardell MD, Terrin ML, et al. Cluster-randomized trial of a mobile phone personalized behavioral intervention for blood glucose control. Diabetes care 2011;**34**(9):1934-42.
42. Pereira K, Phillips B, Johnson C, et al. Internet delivered diabetes self-management education: a review. Diabetes technology & therapeutics 2015;**17**(1):55-63.
43. Lorig K, Ritter PL, Laurent DD, et al. Online diabetes self-management program: a randomized study. Diabetes Care 2010;**33**(6):1275-81.
44. Glasgow RE, Kurz D, King D, et al. Twelve-month outcomes of an Internet-based diabetes self-management support program. Patient education and counseling 2012;**87**(1):81-92.
45. Shirazian S, Crnosija N, Weinger K, et al. The self-management experience of patients with type 2 diabetes and chronic kidney disease: A qualitative study. Chronic illness 2016;**12**(1):18-28.

For peer review only

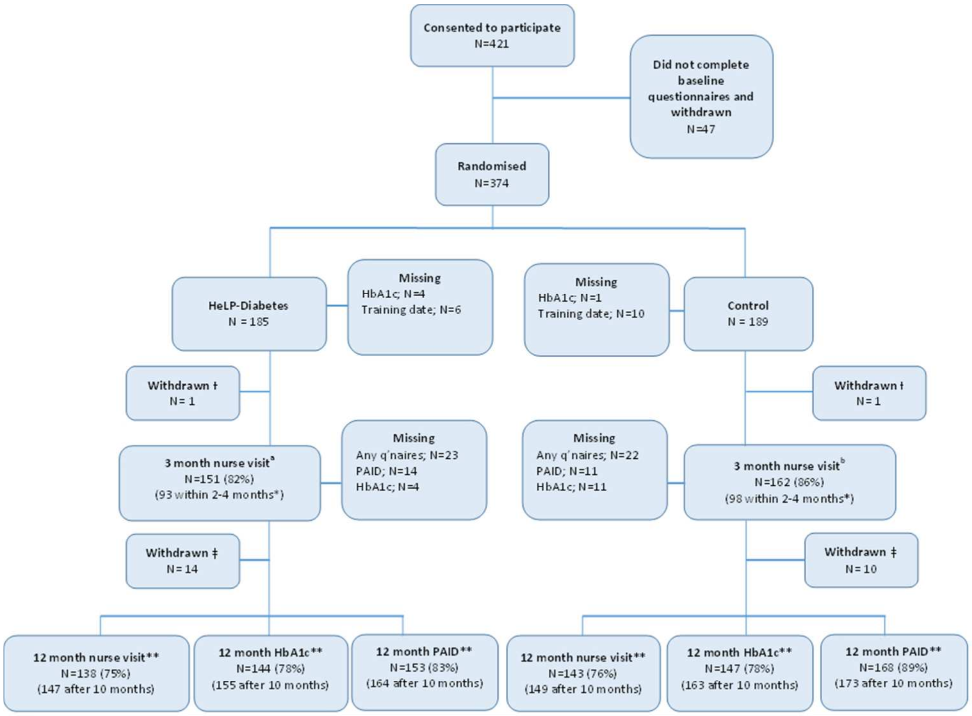

Figure 1: CONSORT diagram showing patient flow through the HeLP-Diabetes randomised controlled trial.

ǂ Withdrawn from the trial before 60 days, ‡ Withdrawn from the trial between 60 and 304 days, * Defined as a visit 60-120 days after randomisation, ** Defined as a visit 300-420 days after randomisation, [a] Range of visits from 63 to 354 days after randomisation, [b] Range of visits from 50 to 253 days after randomisation. Reasons for withdrawal from the trial before the 12-month follow-up are as follows: deteriorating health (n=6), computing issues (n=5), did not attend training (n=5), patient request with no reason given (n=4), moved away (n=2), uncontactable (n=2), family bereavement (n=1), death (n=1)

Figure 1: CONSORT diagram showing patient flow through the HeLP-Diabetes randomised controlled trial.

265x230mm (96 x 96 DPI)

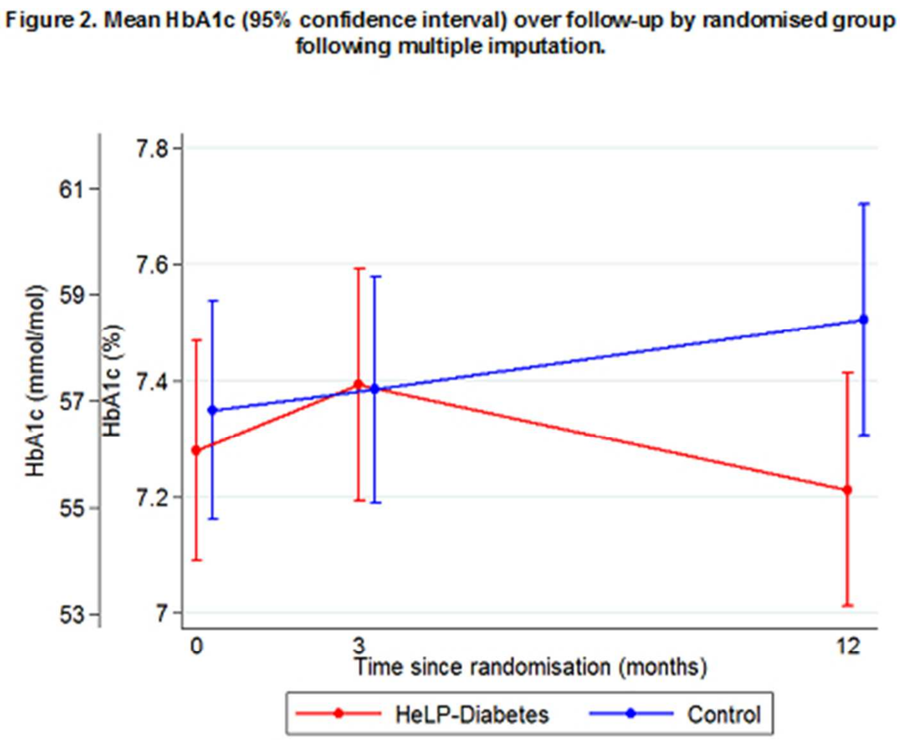

Figure 2. Mean HbA1c (95% confidence interval) over follow-up by randomised group following multiple imputation.

120x99mm (96 x 96 DPI)

Figure 3. Mean PAID score (95% confidence interval) over follow-up by randomised group following multiple imputation

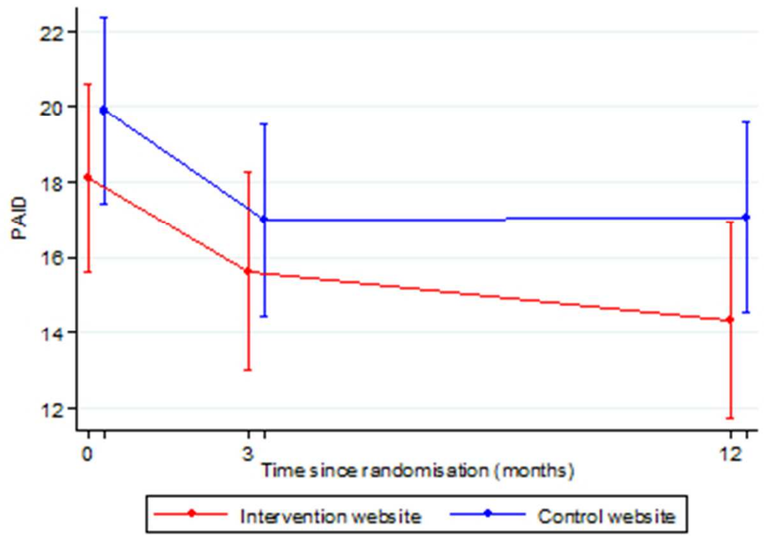

Figure 3. Mean PAID score (95% confidence interval) over follow-up by randomised group following multiple imputation

120x99mm (96 x 96 DPI)

Appendix 1: description of the intervention.[1]

Title: Healthy Living for People with type 2 Diabetes (HeLP-Diabetes): a web-based self-management programme for people with type 2diabetes.

Rationale

The aim of HeLP-Diabetes was to enable people with type 2 diabetes to lead healthier lives and improve overall quality of life. In order to do this, we adopted a strong theoretical framework, which included:

1. The Corbin and Strauss model of living with a long term condition,[2] which determined the overall scope of the intervention;
2. Normalisation Process Theory,[3] to maximise likelihood of successful implementation of the intervention into routine health care
3. A taxonomy of behaviour change techniques,[4] to guide the content of the behaviour change modules;
4. Finally, we worked within the paradigm of evidence-based medicine, ensuring all content was congruent with current best evidence. This applied both to the medical content (e.g. information about treatments) and to the delivery of the intervention (e.g. evidence about maximising acceptability, uptake and adherence).

Self-management of a long term condition is a complex process, which Corbin and Strauss characterised as requiring medical, emotional and role management.[2] Medical management includes behaviour change, such as eating healthily, being physically active, stopping smoking and taking medication. Working with health professionals and the health care system (e.g. keeping appointments, remembering to schedule check-ups or regular monitoring tests) is also included in medical management. Equally important, and often just as challenging for individuals concerned, are emotional management and role management. Emotional management refers to managing the complex negative emotions that result from being diagnosed with a long term condition, such as depression, anger, guilt, shame and a sense of stigma. Role management requires adjusting to the change in biographical narrative, acceptance of the role of "patient", and negotiating inevitable changes in relationships with family, friends and colleagues. This characterisation of the work of self-management provided the overall framework for the content, tone and style of the intervention. As these tasks are intertwined and inter-dependent, we designed the content to address all three tasks in an integrated fashion.

Normalisation Process Theory is a mid-range sociological theory which predicts and explains whether and why certain innovations will be effectively implemented into routine health care and become "normalised", that is, become so much part of routine practice that they disappear from view.[3] It posits that interventions which are easily understood and distinguished from other interventions (coherence), which relevant professionals can see the advantage of compared to current or alternative practice (cognitive participation), which fit well with existing professional relationships and workflows (collective action), and where users are provided with formal or informal feedback and evidence about the impact of the intervention (reflexive monitoring) are more likely to be implemented than those that do not. These constructs guided the development of the intervention by, for example, ensuring HeLP-Diabetes integrated well with existing workflows in general practice.

Effective self-management in diabetes often requires patients to change deep-rooted behaviours, particularly around diet and physical activity. Rather than adopt a single behavioural theory, we opted to design our behaviour change modules around specific

For peer review only - http://bmjopen.bmj.com/site/about/guidelines.xhtml

behaviour change techniques, selecting those with the best evidence base to support their use.[4]

Content

The overall content was broken down into 8 sections:

1. Understanding diabetes (information about the nature and causes of diabetes, and how it affects the body);
2. Staying healthy (motivational material about how to maintain optimal physical and emotional health and the importance of self-management; new behaviour change modules and previously validated programmes for diet, weight loss, physical activity, smoking cessation, moderating alcohol intake, and taking medicines);
3. Treating diabetes (information about medications used in diabetes, including information about indications, side effects and monitoring; importance of managing cardiovascular risk factors as well as glycaemic levels; importance of regular monitoring to prevent retinopathy, neuropathy and nephropathy; and types and roles of different health care professionals involved in caring for people with diabetes);
4. Living and working with diabetes (focus on managing social and work situations, such as shift work, parties, or holidays; impact on relationships, including sexual relationships; and possible impact on emotions and feelings of self-worth);
5. Managing my feelings (self-assessment tools for identifying low mood; cognitive behavioural therapy modules; mindfulness-based approaches);
6. My health record (opportunity to record appointments with health care professionals, results of tests or self-monitoring, with opportunities for graphical displays and feedback);
7. News and Research (updates about latest research or news about diabetes treatment, in depth articles about seminal research papers);
8. Forum and Help (moderated forum; videos of personal stories about diabetes (used with license from health talk online); additional resources, with local resources tailored according to Clinical Commissioning Group (CCG)).

Where possible we aimed to use existing modules with a proven evidence-base. Hence we used, with licence, the entire type 2 diabetes module from Health Talk Online, which contains videos of interviews with people with diabetes from a range of socio-economic and ethnic backgrounds, talking about how they were diagnosed, the impact diabetes has had on their lives and relationships, how they have adapted or come to terms with it, and treatments they have experiences (see www.healthtalk.org). Behaviour change programmes used with license included: POWeR (Positive Online Weight Reduction), a set of 12 modules shown to be effective for managing obesity in primary care[5]; Stop Advisor (smoking cessation)[6] and Down Your Drink (alcohol moderation).[7] New modules were developed for healthy eating, increasing physical activity and taking medication. These used the behaviour change techniques of action planning, goal setting, self-monitoring, reminders and feedback, along with information about the benefits of change. The section on “Managing my feelings” included the *Living Life to the Full* programme[8] – a set of 10 modules based on the principals of cognitive behavioural therapy, specifically adapted for people with type 2 diabetes, and used with license.

Procedures

For peer review only - http://bmjopen.bmj.com/site/about/guidelines.xhtml

HeLP-Diabetes was designed to be used as part of an overall package of care for people with diabetes. Low usage, or non-adherence to internet interventions is well-recognised problem, and our preparatory work with patients and health care professionals indicated that integrating the intervention into routine care was likely to improve uptake and adherence. Hence we made the programme available to registered users only, and encouraged health care professionals to register patients. Once registered, patients could use the programme as much (or as little) as they wanted. There was no prescribed level of use, as our proposed users included patients at all stages of their illness journey, from those newly diagnosed to those who had lived with diabetes for many years. As such, we anticipated that each user would have different needs and priorities, and the programme was designed to allow users to pick and choose sections that were most relevant and beneficial for them personally. There was a limited amount of tailoring. Additional resources and sources of help were tailored by the patient's CCG, but otherwise tailoring was limited to the behaviour change and health record sections where users entered their own goals or data.

Registration and facilitation.

During the trial, registration was performed by a practice nurse. During the registration procedures users were asked to select a password and username. After registration, the nurse was asked to demonstrate the intervention to the patient, and integrate use of the intervention into the patient's personal diabetes care plan by, for example, discussing what goals the patient would like to set for the coming period, and showing the patient how to use the intervention to set goals, monitor progress, and, if desired, arrange for automated reminders by SMS or email to be sent by the programme.

Registration was undertaken in the patient's practice. All subsequent use of the intervention was at any location convenient for the patient with internet access. For most people, we expected this to be at home, or at the home of a relative. However, all users were given information about local services (usually libraries) offering free access to an internet-connected computer.

Encouraging engagement

In addition to the registration and facilitation procedures described above, engagement to the programme was encouraged through regular prompts, provided by email or SMS. Emailed prompts were of two types: a) short (2 – 3 lines) emails with one message and a link to the relevant section of the programme. An example of this type of prompt was a seasonal reminder of the importance of flu vaccination for people with diabetes, with a link to the page of the programme explaining how and why flu vaccination is beneficial, and a reminder that flu vaccination is available free from their general practice. The second type of email prompt was a longer (1 page of A4) newsletter, which summarised recent research or news stories, explained what this meant for patients, and also contained a paragraph or two on specific health promotion – for example, the importance of regular foot care, how to fast safely during Ramadan, or tips for safe overseas travel. These stories and paragraphs also contained links to relevant sections of the programme. SMS prompts were very brief, with one clear message, and a link to the programme. We sent around 3 prompts per month – usually 2 short emails or SMS plus one longer newsletter. Users could opt out of receiving prompts.

For peer review only - http://bmjopen.bmj.com/site/about/guidelines.xhtml

Modifications during the trial

One of the key functions of the programme was to provide up-to-date, evidence-based information. Hence the site was regularly reviewed to ensure all content was up-to-date, evidence-base, and congruent with current NICE guidelines. In practice, this meant small updates each month, with a complete review when the NICE guidelines on management of diabetes were updated.[9]

Assessment of fidelity

Intervention use was assessed through bespoke software which recorded the date, time and pages viewed for each log in by each user. Practice nurses were trained in registration and facilitation procedures, but we were unable to monitor how well they adhered to them.

Availability of the intervention

The intervention is currently available for commissioning from a not-for-profit Community Interest Company, HeLP-Digital. The open access sections of the intervention can be viewed at http://www.helpdigitalcic.org.uk/products

References

1. Hoffmann TC, Glasziou PP, Boutron I, et al. Better reporting of interventions: template for intervention description and replication (TIDieR) checklist and guide. *BMJ (Clinical research ed)* 2014; **348**: g1687.
2. Corbin JM, Strauss A. Unending Work and Care. First ed. San Francisco: Jossey-Bass Inc; 1988.
3. May C, Finch T. Implementation, embedding, and integration: an outline of Normalization Process Theory. *Sociology* 2009; **43**(3): 535-54.
4. Abraham C, Michie S. A taxonomy of behavior change techniques used in interventions. *Health Psychol* 2008; **27**(3): 379-87.
5. Yardley L, Ware LJ, Smith ER, et al. Randomised controlled feasibility trial of a web-based weight management intervention with nurse support for obese patients in primary care. *The international journal of behavioral nutrition and physical activity* 2014; **11**: 67.
6. Brown J, Michie S, Geraghty AW, et al. Internet-based intervention for smoking cessation (StopAdvisor) in people with low and high socioeconomic status: a randomised controlled trial. *The Lancet Respiratory medicine* 2014; **2**(12): 997-1006.
7. Wallace P, Murray E, McCambridge J, et al. On-line Randomized Controlled Trial of an Internet Based Psychologically Enhanced Intervention for People with Hazardous Alcohol Consumption. *PLoSONE* 2011; **6**(3): e14740.
8. Williams C, Association CMH. Living life to the full: Media innovations; 2008.
9. NICE. Type 2 diabetes in adults: Management. NG28. London, 2015.

For peer review only - http://bmjopen.bmj.com/site/about/guidelines.xhtml

TiDIER CHECKLIST.

Item	Line numbers
Provide the name or a phrase that describes the intervention	2,3
Describe any rationale, theory or goal of the elements essential to the intervention	4 – 48
Materials: describe any physical or informational materials used in the intervention, including those provided to participants or used in intervention delivery or in training of the intervention providers	51-90
Procedures: Describe each of the procedures, activities and /or processes used in the intervention, including any enabling or support activities	92 - 137
For each category of intervention provider, describe their expertise, background, and any specific training given.	109
Describe the modes of delivery (such as face-to-face or by some other mechanism, such as internet or telephone) of the intervention, and whether it was provided individually or in a group	2, 123 - 137
Describe the type(s) of location(s) where the intervention occurred, including any necessary infrastructure or relevant features	116 - 120
Describe the number of times the intervention was delivered and over what period of time including the number of sessions, their schedule and their duration, intensity or dose.	98 - 106
If the intervention was planned to be personalised, titrated or adapted, then describe what, why, when and how.	101 - 106
If the intervention was modified during the course of the study, describe the changes (what, why, when and how)	139 - 144
Planned: if intervention adherence or fidelity was assessed, describe how and by whom, and if any strategies were used to maintain or improve fidelity, describe them.	147 - 149
Actual: if intervention adherence or fidelity was assessed, describe the extent to which the intervention was delivered as planned.	147 - 149

For peer review only

Appendix 2: Additional statistical methods and results

Statistical Methods for Multiple Imputation

Multiple Imputation using chained equation was used as the primary method to account for missing data (in both baseline and follow-up data).[1] A set of imputation models were specified, one for each variable with missing data. Each variable was then regressed on all other variables, including completely recorded baseline and follow-up variables and stratified by randomised group. Imputations were performed using predictive mean matching using the five nearest neighbours to the prediction as a set to draw from. The full list of variables considered in the MICE approach is shown in Supplementary Table 1, together with the number of missing values for each variable and time period.

Since only measurements within a 10-14 month window period were used within the main analyses of HbA1c and PAID, the following imputation procedure was implemented for these two co-primary outcomes. Twelve month measurements were subdivided into those that were measured within 10-14 months (the primary outcome variable) and those that were measured outside 10-14 months (a variable used for imputing only). For HbA1c, two additional variables were created for use within the imputation model; 1) the time in days from randomisation that the “12-month” HbA1c measurement was actually taken for values inside of the window period (and set to 365 for measurements taken outside the window period), 2) the time in days from randomisation that the “12-month” HbA1c measurement was actually taken for values outside the window period (and set to missing for measurements taken within the window period). The first of these variables gives the desired time for imputing HbA1c measurements when they are missing, whilst the second gives information on how far outside of the window the actual measurements were taken. Corresponding variables were created for the “12-month” PAID measurement. Finally, two additional variables were created defining the time in days at which HbA1c and PAID were measured at “3-months”. All variables were included within the chained equations and imputed where necessary.

40 imputed datasets were created, the analysis models were fitted to each imputed dataset separately, and the estimates were pooled using Rubin’s rules.

Statistical Methods for causal analyses

The causal analysis proposed attempts to address how the effectiveness of the intervention is mediated through the frequency of website usage. In particular, it is important to understand whether prolonged usage of the website modifies the efficacy of the intervention. Since website usage is measured post-randomisation a naïve analysis of correlating usage with outcomes in the intervention group may give biased and misleading results, since there may be unmeasured confounders also correlated with the outcomes that distinguish the motivated users who regularly log-in from the less motivated ones. Causal analyses using instrumental variables (IV) were therefore used to determine the effect of website usage on outcomes. This approach preserves randomisation (i.e. provides a comparison independent of observed and unobserved confounders).

“Usage” is defined as the proportion of follow-up (rescaled as no. days in a year) that the HeLP-Diabetes website is accessed. It was assumed that the efficacy of the intervention is zero for individuals who never log-in (the exclusion restriction assumption). Website usage in the control group was ignored in the model as it was assumed that the control website was unlikely to be effective. (NB. usage statistics were collected for the comparator website but were not used in these analyses).

For peer review only

This main underlying assumption of the causal analysis is that the effect of randomisation to the HeLP-Diabetes intervention on 12-month outcomes occurs only through use of the website (Supplementary Figure 1; Z is randomised intervention, WU is website-usage and Y is 12-month outcome, e.g. HbA1c or PAID). This relies of the "exclusion restriction" assumption that the HeLP Diabetes intervention has no effect when usage is zero (i.e. for individuals who never log-in). Hence randomisation is assumed to be an instrumental variable.

Figures 4 and 5 show the estimated causal effects of HeLP Diabetes on HbA1c and PAID at 12-months, respectively, by level of website usage (the "exclusion restriction" assumption leads to zero efficacy at zero usage). To determine the casual estimate of efficacy for a "high-usage" group, the individual predicted efficacy was calculated for each individual greater than or equal to the median usage of 4 days (shown as the red boxes in the histograms in Figures 4 and 5) and the average efficacy for this group was obtained. The mean usage in the "high-usage" group was 18 days.

Supplementary Figure 1. Causal model assuming no direct effect of intervention (complete mediation through website usage).

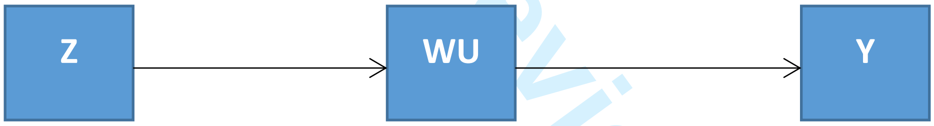

In this causal model, randomisation (Z) is the instrumental variable, which acts on the outcome (Y) exclusively through website usage (WU).

Supplementary Figure 2: Efficacy of HeLP-Diabetes on HbA1c at 12-months based on number of days usage, superimposed by the distribution of usage observed in the HeLP-Diabetes group. The bars shown in red highlight the "high-usage" group that have usage greater than or equal to the median of 4 days.

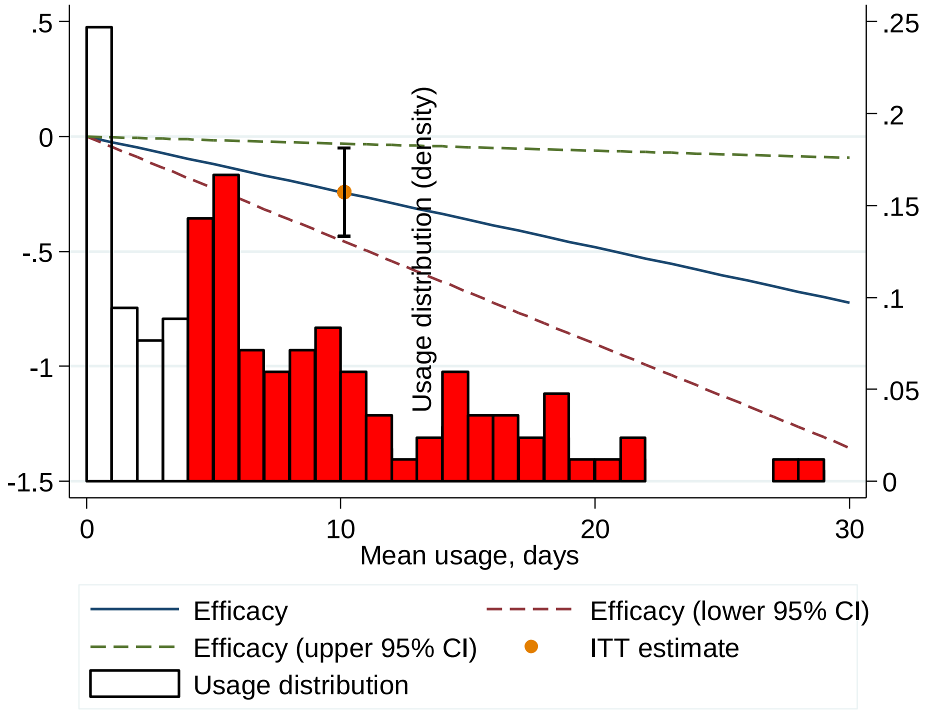

Supplementary Figure 3. Efficacy of HeLP-Diabetes on PAID score at 12-months based on number of days usage, superimposed by the distribution of usage observed in the HeLP-Diabetes group. The bars shown in red highlight the "high-usage" group that have usage greater than or equal to the median of 4 days.

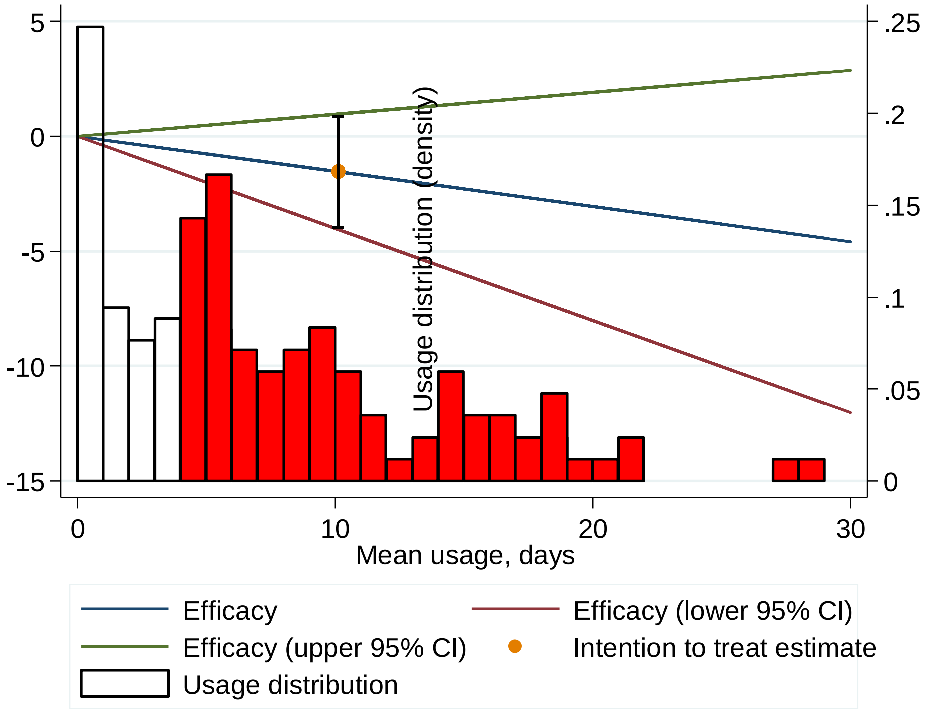

For peer review only

For peer review only

Supplementary Figure 4. Means and 95% CIs of HbA1c over time by randomised group and completers vs. non-completers, after multiple imputation of non-completers follow-up

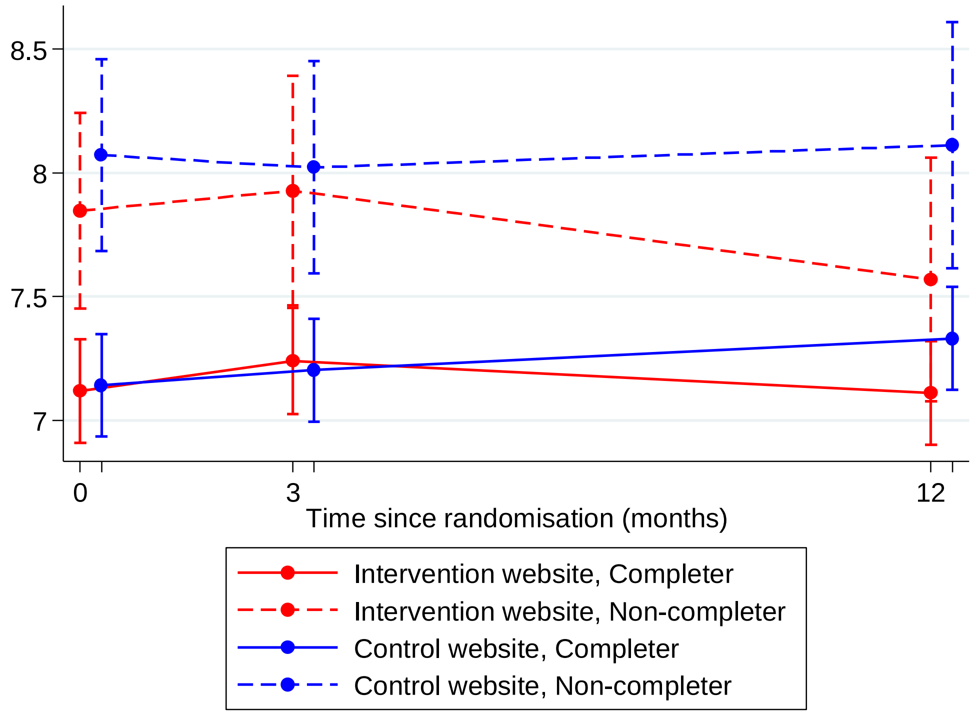

view only

Supplementary Table 1. List of variables imputed and amount of missing data at baseline, 3 months and 12 months.

Variable	Number of missing values		
	Baseline	3 months	12 months
HbA1c	5	64	83 10-14months †
			347 Outside 10-14months †
PAID	0	54	53 10-14months †
			358 Outside 10-14months †
Systolic Blood Pressure	0	57	68
Diastolic Blood Pressure	0	57	68
Body Mass Index	2	58	69
Total cholesterol	2	68	75
HDL cholesterol	12	73	76
Completion of 9 essential processes	69	-	69
HADS	0	74	107
DMSES	0	73	109
DTSQ	0	77	109
Age	0	-	-
Sex	0	-	-
Duration of diabetes	4	-	-
History of cardiovascular disease	0	-	-
Attending any other self-management class	0	-	-
Smoking status	0	-	-
No of visits to the website	-	-	0
No of webpages visited	-	-	105
Average time per visit	-	-	105

* Partial mean matching performed using 5 nearest neighbours

† 12-month measurements were subdivided into those that were measured within 10-14 months (primary outcome variable) and those that were measured outside 10-14 months (variable used for imputing only).

For peer review only

Supplementary Table 2: Descriptive statistics of 3-month variables by randomised group. Mean (SD) unless otherwise specified.

	HeLP-Diabetes	Control	p-value*	N missing
**Clinical Measures**				
Systolic blood pressure (mmHg)	133 (16)	134 (17)	0.350	57
Diastolic blood pressure (mmHg)	76 (10)	75 (9)	0.699	57
Total cholesterol (mmol/l)	4.17 (0.97)	4.16 (1.04)	0.900	68
HDL-C (mmol/l)	1.29 (0.37)	1.24 (0.37)	0.356	73
Total cholesterol /HDL-C ratio	3.48 (1.15)	3.52 (1.09)	0.570	74
HbA1c (%)	7.34 (1.48)	7.34 (1.24)	0.323	64
Body mass index (Kg/m^2)	30.0 (5.4)	30.0 (5.6)	0.545	58
**Questionnaires / scores**				
PAID (0-100)	15.2 (15.2)	16.7 (17.1)	0.492	54
HADS (0-42)	8.84 (6.54)	8.86 (7.45)	0.603	74
Anxiety scale (0-21)	4.75 (3.88)	5.07 (4.24)	0.639	74
Depression scale (0-21)	4.10 (3.30)	3.80 (3.59)	0.205	74
DMSES (0-150)	103.9 (32.2)	104.9 (32.7)	0.795	73
DTSQ (0-48)	32.8 (6.9)	32.9 (6.3)	0.715	77

* Wilcoxon rank-sum test

For peer review only

Supplementary Table 3. Effect of HeLP-Diabetes vs. Control on 12-month primary outcomes, HbA1c and PAID. Results from primary analyses and six sensitivity analyses

	HbA1c, %	
	Number individuals	mean difference (95% CI) HeLP-Diabetes - Control
Primary Analysis Multiple Imputation of both baseline and outcomes	374	-0.242 (-0.435, -0.049); p=0.014
Sensitivity Analysis 1: (multiple imputation of baseline covariates only)	291	-0.214 (-0.390, -0.038); p=0.017
Sensitivity Analysis 2: (complete cases) ƚ	284	-0.207 (-0.385, -0.028); p=0.023
Sensitivity Analysis 3: (complete cases) ǂ	186	-0.220 (-0.416, -0.025); p=0.027
Sensitivity Analysis 4: (non-contaminated cases) ¥	370	-0.225 (-0.418, -0.032); p=0.023
Sensitivity Analysis 5: Linear model excluding centre*	374	-0.242 (-0.438, -0.047); p=0.015
Sensitivity Analysis 6: Unadjusted model (complete-cases)	291	-0.220 (-0.483, 0.043); p=0.100
	**PAID**	
	Number individuals	mean difference (95% CI) HeLP-Diabetes - Control
Primary Analysis Multiple Imputation	374	-1.54 (-3.94, 0.87); p=0.209
Sensitivity Analysis 1: (multiple imputation of baseline covariates only)	321	-1.31 (-3.65, 1.03); p=0.274
Sensitivity Analysis 2: (complete cases) ƚ	317	-1.29 (-3.66, 1.08); p=0.285
Sensitivity Analysis 3: (complete cases) ǂ	65	-2.22 (-6.35, 1.91); p=0.292
Sensitivity Analysis 4: (non-contaminated cases) ¥	370	-1.57 (-3.99, 0.85); p=0.204
Sensitivity Analysis 5: Linear model excluding centre**	374	-1.54 (-3.98, 0.90); p=0.215
Sensitivity Analysis 6: Unadjusted model (complete-cases)	321	-2.38 (-6.02, 1.26); p=0.199

ƚ excluding outcomes outside 10-14 months post-randomisation and those missing baseline covariates

ǂ excluding outcomes outside 11-13 months post-randomisation and those missing baseline covariates

¥ Excluding patients who were suspected to have been exposed to the alternative intervention

* likelihood ratio test for including centre in the model as a fixed effect; p=0.617 (HbA1c)

** likelihood ratio test for including centre in the model as a fixed effect; p=0.357 (PAID)

For peer review only

For peer review only

Supplementary Table 4. Effect of subgroups on the effectiveness of HeLP-Diabetes in reducing HbA1c and PAID.

Subgroup	HbA1c, %			
	Number individuals	mean difference (95% CI) HeLP-Diabetes - Control		Interaction p-value*
		Complete Case ǂ	Multiple Imputation	
Baseline HbA1c				0.458
<7.5%	254/369	-0.15 (-0.41, 0.11)	-0.14 (-0.39, 0.12)	
≥7.5%	115/369	-0.51 (-0.94, -0.07)	-0.51 (-0.93, -0.09)	
Duration of diabetes				0.425
<6.9 yrs	185/370	-0.34 (-0.59, -0.08)	-0.30 (-0.57, -0.03)	
≥6.9 yrs	185/370	-0.07 (-0.32, 0.18)	-0.18 (-0.45, 0.09)	
	**PAID**			
Baseline PAID				0.066
<12	181/374	-0.3 (-4.6, 4.1)	-0.1 (-4.2, 4.1)	
≥12	193/374	-3.6 (-7.8, 0.7)	-3.9 (-8.2, 0.4)	
Duration of diabetes				0.004
<6.9 yrs	185/370	-2.9 (-6.3, 0.4)	-3.6 (-7.0, -0.1)	
≥6.9 yrs	185/370	0.3 (-3.0, 3.7)	0.4 (-3.1, 3.9)	

ǂ excluding outcomes outside 10-14 months post-randomisation and those missing baseline covariates

* p-value for interaction based on a test of linear trend with the continuous variable.

Reference List

1. White IR, Royston P, Wood AM. Multiple imputation using chained equations: Issues and guidance for practice. *Statistics in medicine* 2011; **30**(4): 377-99.

For peer review only

CONSORT 2010 checklist of information to include when reporting a randomised trial*

Section/Topic	Item No	Checklist item	Reported on page No
**Title and abstract**			
	1a	Identification as a randomised trial in the title	1
	1b	Structured summary of trial design, methods, results, and conclusions (for specific guidance see CONSORT for abstracts)	2
**Introduction**			
Background and objectives	2a	Scientific background and explanation of rationale	4
	2b	Specific objectives or hypotheses	4
**Methods**			
Trial design	3a	Description of trial design (such as parallel, factorial) including allocation ratio	4
	3b	Important changes to methods after trial commencement (such as eligibility criteria), with reasons	4
Participants	4a	Eligibility criteria for participants	4
	4b	Settings and locations where the data were collected	4
Interventions	5	The interventions for each group with sufficient details to allow replication, including how and when they were actually administered	5 + Appendix 1
Outcomes	6a	Completely defined pre-specified primary and secondary outcome measures, including how and when they were assessed	6
	6b	Any changes to trial outcomes after the trial commenced, with reasons	N/a
Sample size	7a	How sample size was determined	6-7
	7b	When applicable, explanation of any interim analyses and stopping guidelines	N/A
Randomisation:			
Sequence generation	8a	Method used to generate the random allocation sequence	5
	8b	Type of randomisation; details of any restriction (such as blocking and block size)	5
Allocation concealment mechanism	9	Mechanism used to implement the random allocation sequence (such as sequentially numbered containers), describing any steps taken to conceal the sequence until interventions were assigned	5
Implementation	10	Who generated the random allocation sequence, who enrolled participants, and who assigned participants to interventions	5
Blinding	11a	If done, who was blinded after assignment to interventions (for example, participants, care providers, those	5

For peer review only - http://bmjopen.bmj.com/site/about/guidelines.xhtml

			5
		assessing outcomes) and how	6
	11b	If relevant, description of the similarity of interventions	
Statistical methods	12a	Statistical methods used to compare groups for primary and secondary outcomes	7
	12b	Methods for additional analyses, such as subgroup analyses and adjusted analyses	7 + Appendix 2
**Results**			
Participant flow (a diagram is strongly recommended)	13a	For each group, the numbers of participants who were randomly assigned, received intended treatment, and were analysed for the primary outcome	7 + Figure 1
	13b	For each group, losses and exclusions after randomisation, together with reasons	Figure 1
Recruitment	14a	Dates defining the periods of recruitment and follow-up	7
	14b	Why the trial ended or was stopped	N/A
Baseline data	15	A table showing baseline demographic and clinical characteristics for each group	Table 1
Numbers analysed	16	For each group, number of participants (denominator) included in each analysis and whether the analysis was by original assigned groups	Figure 1
Outcomes and estimation	17a	For each primary and secondary outcome, results for each group, and the estimated effect size and its precision (such as 95% confidence interval)	8, Table 2, Figures 2 & 3
	17b	For binary outcomes, presentation of both absolute and relative effect sizes is recommended	N/A
Ancillary analyses	18	Results of any other analyses performed, including subgroup analyses and adjusted analyses, distinguishing pre-specified from exploratory	8, Supplementary Table 4
Harms	19	All important harms or unintended effects in each group (for specific guidance see CONSORT for harms)	9
**Discussion**			
Limitations	20	Trial limitations, addressing sources of potential bias, imprecision, and, if relevant, multiplicity of analyses	9 - 10
Generalisability	21	Generalisability (external validity, applicability) of the trial findings	9 - 10
Interpretation	22	Interpretation consistent with results, balancing benefits and harms, and considering other relevant evidence	9 - 10
**Other information**			
Registration	23	Registration number and name of trial registry	7
Protocol	24	Where the full trial protocol can be accessed, if available	Reference 17, page 22
Funding	25	Sources of funding and other support (such as supply of drugs), role of funders	11

*We strongly recommend reading this statement in conjunction with the CONSORT 2010 Explanation and Elaboration for important clarifications on all the items. If relevant, we also recommend reading CONSORT extensions for cluster randomised trials, non-inferiority and equivalence trials, non-pharmacological treatments, herbal interventions, and pragmatic trials. Additional extensions are forthcoming: for those and for up to date references relevant to this checklist, see www.consort-statement.org.

For peer review only

For peer review only - http://bmjopen.bmj.com/site/about/guidelines.xhtml

BMJ Open

Web-based self-management support for people with type 2 diabetes (HeLP-Diabetes): randomised controlled trial in English primary care.

Journal:	*BMJ Open*
Manuscript ID	bmjopen-2017-016009.R2
Article Type:	Research
Date Submitted by the Author:	10-Jul-2017

Complete List of Authors:	Murray, Elizabeth; University College London, Research Department of Primary Care and Population Health<br>Sweeting, Michael; MRC Biostatistics Unit, Cambridge,<br>Dack, Charlotte; University of Bath, Department of Psychology<br>Pal, Kingshuk; University College London, Department of Primary Care & Population Health<br>Modrow, Kerstin; University College London, Research Department of Primary Care and Population Health<br>Hudda, Mohammed; MRC Biostatistics Unit, Cambridge,<br>Li, Jinshuo; University of York, Department of Health Sciences<br>Ross, Jamie; University College London, Primary Care & Population Health<br>Alkhaldi, Ghadah; University College London, Research Department of Primary Care and Population Health<br>Barnard, Maria; Whittington Health NHS Trust<br>Farmer, Andrew; University of Oxford, Department of Primary Care Health Sciences<br>Michie, Susan; University College London, Centre for Outcomes Research and Effectivenes<br>Yardley, Lucy; University of Southampton, Academic Unit of Psychology<br>May, Carl; University of Southampton, Faculty of Health Sciences<br>Parrott, Steve; University of York, Department of Health Sciences<br>Stevenson, Fiona; UCL, Primary care and population sciences<br>Knox, Malcolm; University College London, Research Department of Primary Care and Population Health<br>Patterson, David; Whittington Health NHS Trust

**Primary Subject Heading**:	Diabetes and endocrinology
Secondary Subject Heading:	General practice / Family practice, Health informatics, Health services research, Patient-centred medicine
Keywords:	internet, self care, Diabetes mellitus, type 2, PRIMARY CARE

SCHOLARONE™
Manuscripts

For peer review only

Title: Web-based self-management support for people with type 2 diabetes (HeLP-Diabetes): randomised controlled trial in English primary care.

Authors:

Elizabeth Murray[1], Professor of eHealth and Primary Care
Michael Sweeting[2], Senior Research Associate
Charlotte Dack[3], Lecturer,
Kingshuk Pal[1], Clinical Research Associate
Kerstin Modrow[1], Data Manager
Mohammed Hudda[4], Research Fellow in Medical Statistics
Jinshuo Li[5], Research Fellow
Jamie Ross[1], Research Associate
Ghadah Alkhaldi[1] PhD student
Maria Barnard[6], Consultant in Diabetes and Endocrinology,
Andrew Farmer[7], Professor of General Practice
Susan Michie[8], Professor of Health Psychology
Lucy Yardley[7,9], Professor of Health Psychology
Carl May[10], Professor of Health Care Innovation
Steve Parrott[5] Reader in Health Economics,
Fiona Stevenson[1] Reader in Medical Sociology
Malcolm Knox[1] Patient Representative
David Patterson[6], Consultant Cardiologist

[1]Research Department of Primary Care and Population Health, University College London, London NW3 2PF, UK
[2]Cardiovascular Epidemiology Unit, Department of Public Health and Primary Care, University of Cambridge, Worts Causeway, Cambridge, CB1 8RN
[3]Department of Psychology, University of Bath, Claverton Down, Bath, BA2 7AY
[4]Population Health Research Institute, St George's, University of London, Cranmer Terrace, London SW17 0RE
[5]Department of Health Sciences, University of York. Seebohm Rowntree Building, University of York, Heslington, York, YO10 5DD, UK
[6]Whittington Health, Magdala Avenue, London N19 5NF
[7]Nuffield Department of Primary Care Health Sciences, University of Oxford, Radcliffe Observatory Quarter, Woodstock Road, Oxford. OX2 6GG
[8]Centre for Behaviour Change, Research Department of Clinical, Educational and Health Psychology, University College London, 1-19 Torrington Place, London, WC1E 7HB
[9]Department of Psychology, Building 44 Highfield Campus, University of Southampton SO17 1BJ
[10]Health Sciences, University of Southampton, University Road, Southampton SO17 1BJ

Author for correspondence:
Professor Elizabeth Murray, Research Department of Primary Care and Population Health, University College London, London NW3 2PF, UK. Elizabeth.murray@ucl.ac.uk

For peer review only - http://bmjopen.bmj.com/site/about/guidelines.xhtml

For peer review only

Abstract

Objective: To determine the effectiveness of a web-based self-management programme for people with type 2 diabetes in improving glycaemic control and reducing diabetes-related distress.

Methods

Design: Individually randomised two-arm controlled trial

Setting: 21 General Practices in England

Participants: Adults aged 18 or over with a diagnosis of type 2 diabetes registered with participating general practices

Intervention and comparator: Usual care plus ***either*** HeLP-Diabetes, an interactive, theoretically informed, web-based self-management programme ***or*** a simple, text-based website containing basic information only.

Outcomes and data collection: Joint primary outcomes were glycated haemoglobin (HbA1c) and diabetes-related distress, measured by the Problem Areas in Diabetes (PAID) scale, collected at 3 and 12 months after randomisation, with 12 months the primary outcome point. Research nurses, blind to allocation collected clinical data; participants completed self-report questionnaires online.

Analysis: The analysis compared groups as randomised (intention to treat) using a linear mixed effects model, adjusted for baseline data with multiple imputation of missing values.

Results

Of the 374 participants randomised between September 2013 and December 2014, 185 were allocated to the intervention and 189 to the control. Final (12 month) follow up data for HbA1c were available for 318 (85%) and for PAID 337 (90%) of participants. Of these, 291 (78%) and 321 (86%) responses were recorded within the pre-defined "window" of 10-14 months. Participants in the intervention group had lower HbA1c than those in the control (mean difference -0.24%; 95% Confidence Interval -0.44 to -0.049; p=0.014). There was no significant overall difference between groups in the mean PAID score (p=0.21), but pre-specified subgroup analysis of participants who had had diabetes for less than 7 years showed a beneficial impact of the intervention in this group (p = 0.004). There were no reported harms.

Conclusions

Access to HeLP-Diabetes improved glycaemic control over 12 months.

Registration

Trial registration ISRCTN02123133.

Word count of abstract = 299.

For peer review only

Strengths and Limitations of this study

- The trial recruited to target and achieved reasonable follow-up; hence the results for the population of participants are robust (internal validity);
- The two co-primary outcomes reflected the goals of the intervention, namely improving diabetes control and reducing diabetes-related distress;
- However, despite wide inclusion criteria and a deliberately pragmatic design, trial participants were well-controlled at baseline, and therefore the extent to which the trial results generalise to the wider population of people with type 2 diabetes is open to discussion (external validity).

For peer review only

For peer review only - http://bmjopen.bmj.com/site/about/guidelines.xhtml

Introduction.

There is a global epidemic of type 2 diabetes mellitus (T2DM). An estimated 422 million adults, or 10% of the global population, were living with diabetes in 2014 of whom around 90% had type 2 diabetes.[1] Poorly controlled diabetes is associated with premature mortality, and a high risk of complications, including cardiovascular disease, nephropathy and retinopathy. The risk of complications can be reduced by good control of glycaemia and cardiovascular risk factors. [2 3] Interventions which improve self-management skills for patients with diabetes can improve health outcomes and reduce health care costs[4] and international guidelines support training patients in self-management. [3 5] However, it is not clear how best to support patients in developing such skills, and uptake of diabetes self-management education remains low. In England, despite over 90% of eligible patients being referred, [6] only 5.3% attended self-management training in 2014 – 15. [7]

Poor uptake may be related to the dominant model of structured education, which is group-based sessions, lasting a half or whole day, or spread over regular sessions over several weeks. [8] Many patients, such as those who work, those with caring commitments, or those who are uncomfortable in groups, may find it difficult to attend.[9 10]

Web-based support for self-management could address some of these barriers, particularly in high income countries, where levels of web-access are high. In the UK over 80% of households had Internet access in 2015, and internet access amongst older people continues to grow steadily. [11 12] Potential advantages include convenience, anonymity, regular updates, and the potential to use video and graphics to present complex information in a format accessible to those with low literacy.[13] Although systematic reviews have confirmed that computer-based interventions can improve health outcomes in diabetes [14], not all such interventions have a beneficial impact, with meta-analyses showing substantial heterogeneity related to widely differing interventions, including in the use of theory to develop the intervention, [15] outcomes, [14 16] and the duration of follow-up, with most trials having relatively short follow-up (less than 12 months). [14] This is the first UK-based trial of a comprehensive, web-based self-management support programme for people with type 2 diabetes.

This trial assessed the effects of a web-based self-management programme, called Healthy Living for People with Diabetes (HeLP-Diabetes), on glycated haemoglobin (HbA1c) and diabetes-related distress over 12 months.

Methods.

Trial Design and participants: multi-centre, two-arm individually randomised controlled trial in 21 General Practices in England with a mix of urban, suburban and rural practices. Practices were required to have two nurses – one to facilitate access to the intervention, and one to collect data.

Recruitment: standard opt-in recruitment procedures were followed. Each practice had a register of patients with T2DM. The electronic medical record of every patient on this register was reviewed to screen out ineligible patients, and the remainder were sent a letter from their GP, inviting them to participate in the study. Eligible participants were adults, aged 18 or over, with T2DM, registered with participating general practices. Patients were excluded if they were unable to provide informed consent; unable to use a computer due to severe mental or physical impairment; had insufficient spoken or written English to use the intervention (operationalised as unable to consult without an interpreter); were terminally ill with less than 12 months life expectancy; or were currently participating in a trial of an alternative self-management programme. Participants were not required to have home

For peer review only - http://bmjopen.bmj.com/site/about/guidelines.xhtml

Internet access or prior experience of using the Internet to participate. Participants with previous or current experience of self-management education were eligible to participate. Recruitment took place between September 2013 and December 2014. The trial protocol was submitted for publication in June 2014.[17] There were no changes to the methods after the protocol was agreed and the start of the trial. Ethical approval was obtained from Camden and Islington National Research Ethics Service (NRES) committee, reference 12/LO/1571.

Patient involvement

Patients were involved in all stages of the study, including contributing to the original application for funding as co-investigators; substantive and ongoing contribution to intervention development; contributing to the trial design, including the decision to have two co-primary outcomes; active membership of the Trial Steering Committee and Trial Management Group; and contributing to the writing of this paper. This last role is recognised through co-authorship (MK).

Randomisation and blinding

Randomisation marked the point of study entry. It was performed centrally (independently of the trial team), after written informed consent was obtained and all baseline data were completed, using a web-based randomisation system, at the level of the individual participant. Randomisation was conducted in a 1:1 ratio using random permuted blocks of sizes 2, 4 and 6, stratified by recruitment centre. Participants were informed the trial compared two forms of web-based support, and were blinded as to which was the intervention and which the comparator. Nurses who offered facilitation for the intervention could not be blinded, but were asked not to discuss details of allocation with the nurses who gathered follow-up data. The research team obtaining and analysing data from participants were blind to allocation.

Intervention

The intervention consisted of facilitated access to HeLP-Diabetes. Facilitation consisted of an introductory training session with the practice nurse. In this appointment patients were were shown how to log on, set a user name and password, and introduced to the content of the website. HeLP-Diabetes was a theoretically informed web-based programme whose overall goals were to improve health outcomes and reduce diabetes-related distress.[18] Overall content was guided by the Corbin and Strauss model of managing a long term condition which posits that patients must undertake medical, emotional and role management.[19] It was developed using participatory design principles, with substantial input from users, defined as patients with T2DM and health professionals caring for such patients. All content was evidence-based, drawing on evidence on management of diabetes, promoting behaviour change and emotional wellbeing, and maximising usability and engagement. Content was designed to be accessible to people with a wide range of literacy and health literacy skills, with all essential content provided in both video and text. There were information sections on diabetes, how diabetes is treated, possible complications of diabetes, possible impacts of diabetes on relationships at home and at work, dealing with unusual situations like parties, holidays, travelling or shift work, and what lifestyle modifications will improve health. There were sections addressing skills and behaviour change, including behaviour change modules on eating healthily, losing weight, being more physically active, smoking cessation, moderating alcohol consumption, managing medicines, glycaemic control and blood pressure control. Users could set the programme to send themselves reminder text messages or emails, and could specify the content and frequency of such reminders. The third strand of components focused on emotional well-being with self-help tools based on cognitive behavioural therapy and mindfulness. There were multiple personal stories (used with license from health talk online), and a moderated forum. Participants were free to use the programme as much or as little as they chose. Engagement with the programme was promoted through regular newsletters, emails and SMS containing

For peer review only - http://bmjopen.bmj.com/site/about/guidelines.xhtml

updates on latest diabetes-related research or practice, seasonally-relevant advice (e.g. fasting during Ramadan, benefits of ‘flu vaccinations), and links to specific relevant parts of the programme. Two or three prompts were sent each month, although users could opt out of receiving them. Further details are provided in Appendix 1.

Comparator
From an NHS perspective, the important research question was whether the proposed intervention could improve health outcomes when compared to current practice. However, to improve acceptability to participants and to maintain blinding, all participants had access to a website. Participants in the control arm were given access to a simple information website, based on the information available on the website of the main UK diabetes charity (Diabetes UK) or National Health Service patient information website (NHS Choices). They received the same initial facilitation meeting as participants in the intervention group, in which they were shown how to log on, set a user name and password, and how to use the website.

Outcomes and outcome measures

Primary outcomes
The outcomes reflected the dual goals of improving health outcomes and reducing diabetes-related distress. The two joint primary outcomes were glycated haemoglobin (HbA1c) and diabetes-related distress, measured by the Problem Areas in Diabetes (PAID) scale, both at 12 months post-randomisation. PAID has 20 items focusing on areas that cause difficulty for people living with diabetes, including social situations, food, friends and family, diabetes treatment, relationships with health care professionals and social support.[20] PAID scores range from 0 – 100, with higher scores indicating more distress. A score of 40 or more indicates significant distress, and around 40% of patients with diabetes experience significant distress.[21]

Secondary outcomes
Clinical secondary outcomes included systolic and diastolic blood pressure; body mass index; total cholesterol and HDL (not fasting); and completion of the “9 essential processes” for effective management of diabetes, mandated by NHS England (= weight, BP, smoking status, measurement of serum creatinine, cholesterol and HbA1c, urinary albumin and assessment of eyes and feet) within the previous 12 months.[3] Patient-reported outcomes included depression and anxiety, measured using the Hospital Anxiety and Depression Scale (HADS);[22] diabetes-related self-efficacy measured using the Diabetes Management Self-Efficacy Scale (DMSES);[23] and satisfaction with treatment, measured using the Diabetes Satisfaction with Treatment Questionnaire status and change version (DTSQs & DTSQc).[24]

Data collection
Data were collected at baseline, 3 and 12 months, with 12 months the primary endpoint. Patient-reported data were collected using online questionnaires emailed to participants. Clinical outcomes were collected by nurses in participating practices. Participants were asked to complete their online questionnaires before visiting the nurse for clinical measurements and blood tests. Blood samples were analysed at the local NHS laboratory used by participating practices for routine clinical analyses. Data on completion of the “9 essential processes” were collected from the GP record for the 12 months prior to randomisation and the 12 months after randomisation at the 12 month follow-up point to avoid triggering behaviour change amongst the study nurses. Use of the intervention was recorded automatically using bespoke software that recorded the date, and time of each

For peer review only - http://bmjopen.bmj.com/site/about/guidelines.xhtml

For peer review only

page visited. A new log-in to the intervention was defined as any page that was accessed 30 minutes or more after the last accessed page.

Sample size Calculation

Our original sample size calculation was that randomising 350 participants with 85% follow-up would provide 90% power at the 5% level of significance to detect a 0.25% difference in HbA1c and a 4.0 point difference in PAID score at 12 months post-randomisation between the randomised groups.[25 26] Since HbA1c and PAID were joint primary outcomes measuring different aspects of T2DM, both were tested at a 5% significance level.

Analysis

The analysis followed a pre-specified analysis plan, based on comparing the groups as randomised (intention-to-treat). The analysis plan was approved by the Trial Steering Committee before unblinding and uploaded to the trial website, https://www.ucl.ac.uk/pcph/research-groups-themes/ehealth/projects/projects/helpdiabetesrct. Only HbA1c and PAID measured within a 10-14 month window period following randomisation was used in the primary analysis with missing 12-month outcomes multiply imputed using baseline and other outcome data (e.g. 3m data and final follow-up data collected outside the 10-14 month window). Further information on the imputation method is given in Appendix 2.

A linear mixed effects model with random centre effects was used to analyse each of the primary outcomes separately, adjusting for the baseline level of the outcome, age, gender, previous participation in other self-management programmes, pre-existing cardiovascular disease and time since diagnosis of diabetes. Secondary outcome measures were analysed similarly using generalised linear mixed models, with a normal residual error structure for continuous outcomes and a logit link for the binary outcome `Completion of 9 essential processes'. Pre-specified sub-group analysis for the co-primary outcomes was undertaken by baseline glycaemic control (HbA1c outcome only), baseline PAID (PAID outcome only), and duration of diabetes, treating all potential effect modifiers as continuous. The interaction between randomised group and each effect modifier was included in the model separately and assessed using a Wald test.

Use of the intervention was investigated as a mediator for efficacy, using instrumental variable methods, with randomisation as the instrument (Supplementary Figure 1).[27 28]

Potential contamination was monitored by recording participants with similar family names and identifying those with the same addresses. Where this occurred, it was dealt with in the analysis by reporting the extent and undertaking a sensitivity analysis excluding these individuals.

A number of other sensitivity analyses were performed to assess the robustness of the primary analyses: 1) performing two complete case analyses disregarding outcomes measured outside 10-14 months and 11-13 months post-randomisation; 2) repeating the analysis using multiple imputation of baseline covariates only; 3) fitting linear models excluding centre random-effects; and 4) fitting an unadjusted model using only outcome measured in 10-14 months post-randomisation.

The TSC took on the role of the data monitoring committee. Trial registration ISRCTN02123133.

Results

For peer review only - http://bmjopen.bmj.com/site/about/guidelines.xhtml

Recruitment took place between September 2013 and December 2014. An initial 421 patients consented to participate, but of these 47 did not fully complete their baseline questionnaires and were therefore not randomised and did not enter the study. A total of 374 participants were randomised, of whom 86% (n = 321) provided data on PAID, and 78% (n = 291) had HbA1c measured within 10 to 14 months of randomisation. Additional final outcome data, obtained outside the 10 – 14 month pre-defined window, were available for a further 27 participants for HbA1c and 16 participants for PAID (Figure 1). Data obtained outside the 10 – 14 month window were not used directly in the primary analysis, but were entered into the imputation model (Supplementary Table 1).

Baseline characteristics

Baseline demographic and clinical characteristics are shown in Table 1. The mean age was nearly 65 years, over two-thirds (n = 258, 69%) were male, and most were White British (n = 300, 80%). Nearly all (n=370, 99%) had a computer with access to the internet at home and just over half (n = 210, 56%) rated themselves as experienced computer users. Around one-third (n = 134; 36%) had been diagnosed for less than 5 years, with a further third (n = 115, 31%) having been diagnosed between 5 and 9 years ago. Overall, this was a population with well-controlled diabetes at baseline (mean HbA1c was 7.3% (56 mmol / mol)) and low levels of distress (mean PAID = 19).

Primary outcomes

At twelve months the primary analysis showed a significant difference in change in HbA1c between the randomised groups with participants in the HeLP-Diabetes group having a lower HbA1c than those in the control group (mean difference = -0.24%; 95% confidence intervals -0.44 to -0.049, p=0.014) (Table 2, Figure 2). There was no difference in change in PAID scores between the groups at 12-months (mean difference -1.5; 95% CI -3.9, 0.9, p=0.209), though both groups showed a decrease in PAID over the follow-up of the trial (Table 2, Figure 3).

Secondary outcomes

There was no difference in secondary outcomes at 12 months, with the possible exception of systolic blood pressure, which decreased more in the intervention group than in the control group (p=0.010) (Table 2); though the result was not statistically significant after correction for multiple testing of secondary outcomes. There were no significant differences between groups on any of the outcome measures amongst individuals who completed three month outcomes (Supplementary Table 2). No adverse effects or events were recorded during follow-up.

Usage data

The mean number of log-ins was significantly higher in the intervention group than the control group (18.7 vs. 4.8, p= 0.0001), as was the mean number of pages visited per log-in (10.5 vs. 7.7, p <0.0001) and the mean number of days in which the website was accessed (10.1 vs. 3.3, p<0.0001) (Table 3). The causal analyses estimated that for a "high-usage" population (those with usage ≥ the median of 4 days) the HeLP-Diabetes intervention could on average reduce HbA1c by -0.44% (95% CI -0.81 to -0.06) and PAID by -2.8 (95% CI -7.2 to 1.7) over 12 months (Supplementary Figures 2 and 3). The mean usage in the "high-usage" group was 18 days. It should be noted that the usage data presented do not include the initial facilitation visit. There was a technical error in the software which led to usage data not being collected before 1 January 2014. At this point 16 participants had been randomised (7 to intervention, 9 to control). For these 16 participants, the usage data is not based on a full year, but for all other participants, data are summarised for the 12 months post-randomisation.

Sensitivity analyses

For peer review only - http://bmjopen.bmj.com/site/about/guidelines.xhtml

The findings from the sensitivity analyses, including a complete-case analysis, were similar to the main analysis (Supplementary Table 3). Participants who were missing 12-month HbA1c had significantly higher mean baseline HbA1c measures (7.9% vs. 7.1%, p<0.001) leading to higher imputed HbA1c at 12-months in the non-completers and a greater mean difference between the randomised groups than from complete case analyses (Supplementary Figure 4, Supplementary Table 3).

Subgroup analyses

Pre-specified subgroup analyses showed that there was no evidence of baseline measures of HbA1c or PAID being effect modifiers for the mean difference between the groups. There was strong statistical evidence (interaction p=0.004) to suggest that the duration of diabetes acted as an effect modifier, with those who had been diagnosed more recently (<7 years) showing more of a reduction in PAID than those who had been diagnosed for longer periods of time. Duration of diabetes had no effect on change in HbA1c (Supplementary Table 4).

Harms

There were no reported harms in either group.

Discussion

In this first UK-based trial of a web-based self-management programme for people with T2DM, participants randomised to HeLP-Diabetes demonstrated improved glycaemic control at 12 months compared to those randomised to a simple information website. This improvement appears robust across all pre-specified sensitivity analyses, and was not dependent on duration of diabetes, baseline glycaemic levels or level of diabetes-related distress. Each 1% reduction in HbA1c is associated with a risk reduction of 21% for deaths related to diabetes and a 37% risk reduction for microvascular complications.[26] A reduction in HbA1c of 0.24% across a population level could translate into considerable population benefit, particularly as this web-based intervention could be delivered at low-cost and at scale across the UK. Moreover, in contrast to group-based education, where the effects appear to wane with time,[29] the effects of HeLP-Diabetes were greater at 12 months than at 3 months. There was no overall impact on diabetes-related distress, but some evidence that HeLP-diabetes appeared to reduce distress in recently diagnosed individuals. However, it is worth noting that baseline PAID scores were exceptionally low in this trial population. In a small pilot study, participants offered supported access to HeLP-Diabetes reduced their PAID scores by 6 points (p=0.04) over 6 weeks.[30]

The trial has many strengths. It was a pragmatic trial, open to nearly all patients with T2DM in participating practices. Concealment of allocation was complete, as randomisation occurred after baseline data collection. Baseline prognostic factors were well balanced between groups. Every effort was made to achieve blinding, including requiring practices to have two nurses, so that data collection was undertaken by a nurse blind to participant allocation. Data for the co-primary outcomes at the primary outcome point were available for 78% and 86% of participants for HbA1c and PAID respectively. All analyses were on an intention-to-treat basis, supplemented by a CACE analysis. Although response rates for the co-primary outcomes were good, some potential for bias existed. Our primary analysis used multiple imputation methods because evidence shows that the assumptions underpinning this method are more defensible than those assumed using other approaches to missing data.[31] We also undertook sensitivity analyses including complete cases, non-contaminated cases, and a linear model excluding centre; all yielded similar results.

The two co-primary outcomes reflected the twin aims of the intervention: to improve diabetes control and to reduce diabetes-related distress. Around 40% of patients with diabetes have significant levels of distress, which severely impacts on quality of life,[32] and diabetes-related distress is an important outcome for patients.[33] Our patient and public involvement (PPI)

For peer review only - http://bmjopen.bmj.com/site/about/guidelines.xhtml

panel were clear that this should be a primary outcome, and a recent meta-ethnography emphasised the importance of empowerment and quality of life in promoting long term engagement with self-management.[34] In contrast, many health care professionals are more interested in glycaemic control. In line with previous trials in this area,[35] we decided to adopt both as co-primary outcomes and to test both at a 5% level of significance.[36]

There are some limitations. Despite maximising the inclusivity of the trial by minimising the exclusion criteria, participants were not representative of the overall population of patients with type 2 diabetes in England. Compared to the overall population, participants had better control of their diabetes and cardiovascular risk factors, [6 7] and were much less distressed. [21] This finding mirrors that of a recent systematic review of demographic factors associated with web portal usage amongst people with diabetes which found that those with well controlled diabetes were more likely to use such portals than those with poor control. [37] However, fewer of our participants self-rated their computer skills as excellent (57% of our sample compared to a national average of 73%).[12] This good control at baseline has two implications – first, that there was little room for improvement in this population, and secondly, that this population may have been unusually motivated to self-manage their diabetes. Although every effort was made to maintain blinding, it is possible that some participants may have discussed their use of the intervention with research nurses, making it possible to infer which arm they had been allocated to. This could have affected research nurses' measurements of secondary clinical outcomes, such as blood pressure or weight, but could not have affected assessment of glycated haemoglobin as this was measured by laboratory staff who were blinded. There appeared to be high potential for contamination between two participants who shared the same surname and address, and a further two participants did not receive their allocated intervention due to an error at practice level; excluding these four made no difference to the results. A further limitation of the trial is that it provides little insight into the mechanism of action of HeLP-Diabetes. This was the result of a deliberate decision to focus on clinically important outcomes and minimise both the response burden and the potential impact of measurement on participants.

This is the first UK-based trial of a web-based self-management programme for people with type 2 diabetes, and internationally, the first trial of such a comprehensive intervention that aims to address the three main tasks of self-management: emotional, medical and role management.[19] In the Cochrane review of computer-based self-management interventions for people with T2DM, only 4 of the included studies had follow-up of 12 months or more.[14] Of these, three interventions were clinic-based, with participants completing self-assessment tools on a touch screen and receiving tailored advice during their baseline visit to their diabetes clinician [38-40] and one was a mobile phone-based intervention which provided tailored messages in response to participant's results of blood glucose self-monitoring data. [41] A more recent systematic review of internet delivered diabetes self-management identified 2 trials with 12 or more months follow-up. [42] One trial was on a structured intervention based on a peer-led, group-based, diabetes self-management course [43]. There were six sessions, with each session available for one week. Each session required participants to make a specific action plan to address a problem they were experiencing. Peer facilitators encouraged use of the programme. Follow up was planned at 6 and 12 months; however HbA1c data were only available at 6 months. The other trial compared two versions of a web-based intervention (with and without additional social support) to enhanced usual care. The web-based intervention was designed using social-cognitive theory and a social-ecological model, with a focus on three main behaviours: dietary intake, physical activity and medication adherence. Users of either web-based intervention received motivational phone calls to encourage adherence and development of action plans. Those randomised to the enhanced intervention (with additional social support) received two additional phone calls and an invitation to attend a group session. There was no difference between groups in HbA1c or other biological outcomes at 12 months. [44] Thus the results of this trial add significantly to the available literature.

For peer review only - http://bmjopen.bmj.com/site/about/guidelines.xhtml

On the basis of these results, HeLP-Diabetes may be considered as an addition to the current menu of self-management support for people with type 2 diabetes, and may help increase overall access and uptake. Most commissioned services currently focus on newly diagnosed patients, leaving clear unmet need for people who have had their diabetes for longer, but are looking for ways to improve their health. Many patients are not ready to engage in self-management early in their illness journey,[9] but become motivated to do so later, often as a result of a change in medication or development of a complication.[45] The intervention is low cost, and as most costs are fixed, irrespective of number of users, is likely to be cost-effective, particularly if widely used. A cost-effectiveness analysis of HeLP-Diabetes will be reported separately.

Word count 4,303

Contributors

EM, MS, CD, KP, MB, AF, SM, LY, CM, SP, FS and DP all contributed to the design of the trial. MS, assisted by MH and KM designed the statistical analysis plan and undertook the analysis. SP and JL designed and undertook the health economic aspects of the trial. EM, KP, CD, JR, GA, LY and SM contributed to the development and delivery of the intervention. MK contributed PPI input. EM and MS wrote the first draft of the paper; all authors commented on this draft and have approved the final version.

Declaration of interests

EM is the Managing Director of a not-for-profit Community Interest Company established to disseminate HeLP-Diabetes across the UK.

Acknowledgements

Funding.

This paper presents independent research funded by the National Institute for Health Research (NIHR) under its Programme Grants for Applied Research Programme (Grant Reference Number RP-PG-0609-10135). The views expressed are those of the author(s) and not necessarily those of the NHS, the NIHR or the Department of Health. The funder had no role in the study design, data collection, data analysis, data interpretation, or writing of the report. All authors had full access to all the data in the study and can take responsibility for the integrity of the data and data analysis. The lead author had final responsibility for the decision to submit for publication, affirms that the manuscript is an honest, accurate, and transparent account of the study being reported; that no important aspects of the study have been omitted; and that there were no significant discrepancies from the published protocol.

Work conducted at the Cardiovascular Epidemiology Unit, University of Cambridge by MS and MH was additionally funded by the UK Medical Research Council (MR/L003120/1), British Heart Foundation (RG/13/13/30194) and UK National Institute for Health Research Cambridge Biomedical Research Centre.

For peer review only

For peer review only - http://bmjopen.bmj.com/site/about/guidelines.xhtml

AF is an NIHR Senior Investigator and receives funding from Oxford NIHR Biomedical Research Centre

We gratefully acknowledge the permission to use under license the validated behaviour change modules for weight loss (POWeR), alcohol reduction (DownYour Drink) and smoking cessation (StopAdvisor and the diabetes module from Healthtalk online (http://www.healthtalk.org/) in HeLP-Diabetes.

We are grateful to Orla O'Donnell for outstanding project management, Fiona Giles for administrative support, all our PPI who contributed to the development, maintenance and delivery of the intervention and / or the management and oversight of the trial, staff at the participating practices, PCRN staff, and all our participants.

Data Sharing.
Patient level data, the full dataset, statistical code are available from the corresponding author. Consent for data sharing was not obtained from participants, but the potential benefits of sharing these data outweigh the potential harms as the data are anonymised.

For peer review only

For peer review only

For peer review only - http://bmjopen.bmj.com/site/about/guidelines.xhtml

Table 1: Descriptive statistics of baseline variables by randomised group. Mean (SD) unless otherwise specified.

	HeLP-Diabetes N=185	Control N=189	N missing
Age at randomisation (yrs)	64.9 (9.5)	64.7 (9.1)	0
Male sex, n (%)	127 (69%)	131 (69%)	0
Ethnicity, n(%):			1
White English, Welsh, Scottish, Northern Irish, British	151 (82%)	149 (79%)	
Indian	12 (6%)	8 (4%)	
Other	21 (11%)	31 (16%)	
Experience with computers, n(%)			0
None	5 (3%)	4 (2%)	
Basic	75 (41%)	80 (42%)	
Experienced	105 (57%)	105 (56%)	
Smoking status, n(%):			0
Current smoker	14 (8%)	14 (7%)	
Former smoker	94 (51%)	86 (46%)	
Never smoker	77 (42%)	89 (47%)	
Time since diagnosis (years), n(%):			4
0-4 years	70 (38%)	64 (34%)	
5-9 years	55 (30%)	60 (32%)	
10-14 years	40 (22%)	40 (21%)	
15+ years	18 (10%)	23 (12%)	
Attending any other self-management class, n(%)	4 (2%)	4 (2%)	0
**Clinical Measures**			
Systolic blood pressure (mmHg)	135 (17)	135 (17)	0
Diastolic blood pressure (mmHg)	78 (11)	77 (10)	0
Total cholesterol (mmol/l)	4.11 (1.03)	4.18 (0.98)	2
HDL-C (mmol/l)	1.24 (0.31)	1.25 (0.36)	12
Total cholesterol /HDL-C ratio	3.43 (1.09)	3.52 (1.03)	13
HbA1c (%)	7.26 (1.25)	7.35 (1.37)	5
HbA1c (mmol/mol)	56 (14)	57 (15)	5
Body mass index (Kg/m^2)	30.1 (5.3)	29.6 (5.2)	2
**Questionnaires / scores**			
PAID (0-100)	18.1 (17.1)	19.9 (19.9)	0
HADS (0-42)	9.28 (6.47)	9.12 (7.52)	0
Anxiety scale (0-21)	4.92 (3.70)	5.21 (4.20)	0
Depression scale (0-21)	4.36 (3.48)	3.91 (3.73)	0
DMSES (0-150)	98.6 (33.9)	103.7 (32.4)	0
DTSQ (0-48)	32.1 (7.3)	32.0 (7.2)	0
Completion of 9 essential processes in previous 12 months, n(%)	97 (64%)	96 (62%)	69

For peer review only - http://bmjopen.bmj.com/site/about/guidelines.xhtml

Table 2: 12 month outcomes, adjusted for relevant baseline outcome, age, sex, current (baseline) participation in other self-management programmes, pre-existing cardiovascular disease and duration of diabetes.
Results from multiply imputed data shown. Data are mean (SE) or mean difference (95% CI) unless otherwise specified.

	HeLP-Diabetes		Control		HeLP-Diabetes vs Control	
	Baseline	Change from baseline to 12 months	Baseline	Change from baseline to 12 months	Mean difference (95% CI)	p-value
**Primary outcomes**						
HbA1c, (%)	7.3 (0.1)	-0.08 (0.07)	7.3 (0.1)	0.16 (0.07)	-0.24 (-0.44, -0.05)	0.014
HbA1c, mmol/mol	56.3 (1.1)	-0.8 (0.8)	56.8 (1.1)	1.8 (0.8)	-2.6 (-4.8, -0.5)	0.014
PAID	18.2 (1.3)	-4.1 (0.9)	19.8 (1.3)	-2.5 (0.9)	-1.5 (-3.9, 0.9)	0.209
**Secondary outcomes**						
Systolic blood pressure, mmHg	134.7 (1.5)	-4.2 (1.4)	134.9 (1.5)	-0.5 (1.4)	-3.8 (-6.6, -0.9)	0.010
Diastolic blood pressure, mmHg	77.8 (1.0)	-2.5 (0.9)	77.1 (1.0)	-1.9 (0.8)	-0.6 (-2.4, 1.2)	0.519
Body mass index, Kg/m^2	30.1 (0.5)	0.12 (0.2)	30.0 (0.5)	-0.04 (0.2)	0.16 (-0.30, 0.62)	0.498
Total cholesterol, mmol/L	4.1 (0.1)	-0.08 (0.06)	4.2 (0.1)	-0.15 (0.06)	0.07 (-0.09, 0.2)	0.370
HDL cholesterol, mmol/L	1.25 (0.03)	-0.003 (0.018)	1.26 (0.03)	0.004 (0.018)	-0.007 (-0.054, 0.039)	0.754
Completion of 9 essential processes*	65% (3.7)	-5.1%	61% (3.8)	3.4%	0.78 (0.45, 1.35)	0.379
HADS	9.3 (0.5)	-1.05 (0.44)	9.1 (0.5)	-0.60 (0.48)	-0.45 (-1.68, 0.78)	0.474
DMSES**	98.8 (2.4)	2.93 (2.90)	103.6 (2.3)	1.38 (2.79)	1.55 (-5.74, 8.84)	0.674
DTSQ	32.2 (0.6)	0.94 (0.57)	32.2 (0.6)	0.45 (0.61)	0.49 (-1.18, 2.15)	0.564

* Percentage (SE) and odds ratio (95% CI)
** linear regression results shown due to lack of convergence for mixed model

For peer review only - http://bmjopen.bmj.com/site/about/guidelines.xhtml

Table 3. Extent of website usage over 12-month follow-up.

	HeLP-Diabetes	Control	p-value*	N missing
Number of log-ins per person	18.7 (84.0)	4.8 (8.0)	0.0001	0
Number pages visited per log-in	10.5 (6.7)	7.7 (5.0)	<0.0001	105**
Time spent in each log-in (minutes)***	12.3 (9.8)	8.2 (8.4)	<0.0001	105**
Number of days in which website was accessed over follow-up	10.1 (22.9)	3.3 (5.1)	<0.0001	0

Mean (SD) unless otherwise specified.
* Wilcoxon rank-sum test.
** 105 individuals did not log in after their facilitation visit (42 intervention, 63 control).
*** Measured as time from first page accessed to last page accessed within a log-in session.

For peer review only

Figure Legends

Figure 1: CONSORT diagram showing patient flow through the HeLP-Diabetes Randomised Controlled Trial.

Figure 2: Mean HbA1c (95% confidence interval) over follow up by randomised group using multiple imputation

Figure 3: Mean PAID score (95% confidence interval) over follow up by randomised group using multiple imputation.

For peer review only

For peer review only - http://bmjopen.bmj.com/site/about/guidelines.xhtml

Reference List

1. Worldwide trends in diabetes since 1980: a pooled analysis of 751 population-based studies with 4.4 million participants. Lancet (London, England) 2016;**387**(10027):1513-30.
2. Tkac I. Effect of intensive glycemic control on cardiovascular outcomes and all-cause mortality in type 2 diabetes: Overview and metaanalysis of five trials. Diabetes Res Clin Pract 2009;**86 Suppl 1:S57-62.**:S57-S62.
3. National Institute of Health and Care Excellence (NICE). Type 2 diabetes in adults: Management. NG28. London, 2015.
4. Panagioti M, Richardson G, Murray E, et al. Self-management support interventions to reduce health care utilisation without compromising outcomes: a systematic review and meta-analysis. BMCHealth ServRes 2014;**14:356. doi: 10.1186/1472-6963-14-356.**:356-14.
5. (4) Foundations of care: education, nutrition, physical activity, smoking cessation, psychosocial care, and immunization. Diabetes care 2015;**38 Suppl**:S20-30.
6. NHS Digital. Quality and Outcomes Framework (QOF) - 2014-15. October 29 2015 ed. London: NHS Digital, 2015.
7. National Diabetes Audit 2013-2014 and 2014-2015 Report 1: Care Processes and Treatment Targets, 2016:1-35.
8. Deakin T, McShane CE, Cade JE, et al. Group based training for self-management strategies in people with type 2 diabetes mellitus. Cochrane Database SystRev 2005;**18**(2):CD003417.
9. Winkley K, Evwierhoma C, Amiel SA, et al. Patient explanations for non-attendance at structured diabetes education sessions for newly diagnosed Type 2 diabetes: a qualitative study. Diabetic medicine : a journal of the British Diabetic Association 2015;**32**(1):120-8.
10. Horigan G, Davies M, Findlay-White F, et al. Reasons why patients referred to diabetes education programmes choose not to attend: a systematic review. Diabetic medicine : a journal of the British Diabetic Association 2017;**34**(1):14-26.
11. Internet Access - Households and Individuals: 2015: Office for National Statistics, 2015:1-14.
12. Dutton WH, Blank G, Groselj D. Cultures of the Internet: The Internet in Britain. Oxford Internet Survery 2013. Oxford: Oxford Internet Institute, 2013:1 - 64.
13. Rowsell A, Muller I, Murray E, et al. Views of People With High and Low Levels of Health Literacy About a Digital Intervention to Promote Physical Activity for Diabetes: A Qualitative Study in Five Countries. Journal of medical Internet research 2015;**17**(10):e230.
14. Pal K, Eastwood SV, Michie S, et al. Computer-based diabetes self-management interventions for adults with type 2 diabetes mellitus. CochraneDatabaseSystRev 2013;**3:CD008776. doi: 10.1002/14651858.CD008776.pub2.**:CD008776.
15. van Vugt M, de Wit M, Cleijne WH, et al. Use of behavioral change techniques in web-based self-management programs for type 2 diabetes patients: systematic review. Journal of medical Internet research 2013;**15**(12):e279.
16. Hadjiconstantinou M, Byrne J, Bodicoat DH, et al. Do Web-Based Interventions Improve Well-Being in Type 2 Diabetes? A Systematic Review and Meta-Analysis. Journal of medical Internet research 2016;**18**(10):e270.
17. Murray E, Dack C, Barnard M, et al. HeLP-Diabetes: randomised controlled trial protocol. BMC health services research 2015;**15**:578.
18. Integrating theory, qualitative data and participatory design to develop HeLP-Diabetes: an internet self-management intervention for people with type 2 diabetes 2013/05/18/; Chicago, USA. International Society for Research on Internet Interventions.
19. Corbin JM, Strauss A. *Unending Work and Care*. First ed. San Francisco: Jossey-Bass Inc, 1988.
20. Polonsky WH, Anderson BJ, Lohrer PA, et al. Assessment of diabetes-related distress. Diabetes Care 1995;**18**(6):754-60.

For peer review only - http://bmjopen.bmj.com/site/about/guidelines.xhtml

21. Nicolucci A, Kovacs Burns K, Holt RI, et al. Diabetes Attitudes, Wishes and Needs second study (DAWN2): cross-national benchmarking of diabetes-related psychosocial outcomes for people with diabetes. Diabetic medicine : a journal of the British Diabetic Association 2013;**30**(7):767-77.
22. Zigmond AS, Snaith RP. The hospital anxiety and depression scale. Acta PsychiatrScand 1983;**67**(6):361-70.
23. Bijl JV, Poelgeest-Eeltink AV, Shortridge-Baggett L. The psychometric properties of the diabetes management self-efficacy scale for patients with type 2 diabetes mellitus. JAdvNurs 1999;**30**(2):352-59.
24. Bradley C, Lewis KS. Measures of psychological well-being and treatment satisfaction developed from the responses of people with tablet-treated diabetes. DiabetMed 1990;**7**(5):445-51.
25. Welch GW, Jacobson AM, Polonsky WH. The Problem Areas in Diabetes Scale. An evaluation of its clinical utility. Diabetes care 1997;**20**(5):760-6.
26. Stratton IM, Adler AI, Neil HA, et al. Association of glycaemia with macrovascular and microvascular complications of type 2 diabetes (UKPDS 35): prospective observational study. BMJ (Clinical research ed) 2000;**321**(7258):405-12.
27. White IR, Kalaitzaki E, Thompson SG. Allowing for missing outcome data and incomplete uptake of randomised interventions, with application to an Internet-based alcohol trial. Statistics in medicine 2011;**30**(27):3192-207.
28. Dunn G, Maracy M, Tomenson B. Estimating treatment effects from randomized clinical trials with noncompliance and loss to follow-up: the role of instrumental variable methods. StatMethods Med Res 2005;**14**(4):369-95.
29. Khunti K, Gray LJ, Skinner T, et al. Effectiveness of a diabetes education and self management programme (DESMOND) for people with newly diagnosed type 2 diabetes mellitus: three year follow-up of a cluster randomised controlled trial in primary care. BMJ 2012;**344:e2333. doi: 10.1136/bmj.e2333.**:e2333.
30. Hofmann M, Dack C, Barker C, et al. The Impact of an Internet-Based Self-Management Intervention (HeLP-Diabetes) on the Psychological Well-Being of Adults with Type 2 Diabetes: A Mixed-Method Cohort Study. Journal of diabetes research 2016;**2016**:1476384.
31. Molenberghs G, Thijs H, Jansen I, et al. Analyzing incomplete longitudinal clinical trial data. Biostatistics (Oxford, England) 2004;**5**(3):445-64.
32. Chew BH, Mohd-Sidik S, Shariff-Ghazali S. Negative effects of diabetes-related distress on health-related quality of life: an evaluation among the adult patients with type 2 diabetes mellitus in three primary healthcare clinics in Malaysia. Health and quality of life outcomes 2015;**13**:187.
33. Glasgow RE, Peeples M, Skovlund SE. Where is the patient in diabetes performance measures? The case for including patient-centered and self-management measures. Diabetes Care 2008;**31**(5):1046-50.
34. Frost J, Garside R, Cooper C, et al. A qualitative synthesis of diabetes self-management strategies for long term medical outcomes and quality of life in the UK. BMC health services research 2014;**14**:348.
35. Kinmonth AL, Woodcock A, Griffin S, et al. Randomised controlled trial of patient centred care of diabetes in general practice: impact on current wellbeing and future disease risk. The Diabetes Care From Diagnosis Research Team. BMJ (Clinical research ed) 1998;**317**(7167):1202-8.
36. Schulz KF, Grimes DA. Multiplicity in randomised trials I: endpoints and treatments. Lancet (London, England) 2005;**365**(9470):1591-5.
37. Amante DJ, Hogan TP, Pagoto SL, et al. A systematic review of electronic portal usage among patients with diabetes. Diabetes technology & therapeutics 2014;**16**(11):784-93.

For peer review only - http://bmjopen.bmj.com/site/about/guidelines.xhtml

For peer review only

38. Christian JG, Bessesen DH, Byers TE, et al. Clinic-based support to help overweight patients with type 2 diabetes increase physical activity and lose weight. Arch Intern Med 2008;**168**(2):141-6.
39. Glasgow RE, La Chance PA, Toobert DJ, et al. Long term effects and costs of brief behavioural dietary intervention for patients with diabetes delivered from the medical office. Patient education and counseling 1997;**32**(3):175-84.
40. Glasgow RE, Nutting PA, King DK, et al. Randomized effectiveness trial of a computer-assisted intervention to improve diabetes care. Diabetes care 2005;**28**(1):33-9.
41. Quinn CC, Shardell MD, Terrin ML, et al. Cluster-randomized trial of a mobile phone personalized behavioral intervention for blood glucose control. Diabetes care 2011;**34**(9):1934-42.
42. Pereira K, Phillips B, Johnson C, et al. Internet delivered diabetes self-management education: a review. Diabetes technology & therapeutics 2015;**17**(1):55-63.
43. Lorig K, Ritter PL, Laurent DD, et al. Online diabetes self-management program: a randomized study. Diabetes Care 2010;**33**(6):1275-81.
44. Glasgow RE, Kurz D, King D, et al. Twelve-month outcomes of an Internet-based diabetes self-management support program. Patient education and counseling 2012;**87**(1):81-92.
45. Shirazian S, Crnosija N, Weinger K, et al. The self-management experience of patients with type 2 diabetes and chronic kidney disease: A qualitative study. Chronic illness 2016;**12**(1):18-28.

For peer review only

For peer review only - http://bmjopen.bmj.com/site/about/guidelines.xhtml

Unable to Convert Image

The dimensions of this image (in pixels) are too large to be converted. For this image to convert, the total number of pixels (height x width) must be less than 40,000,000 (40 megapixels).

Figure 1: CONSORT diagram showing patient flow through the HeLP-Diabetes Randomised Controlled Trial.

For peer review only

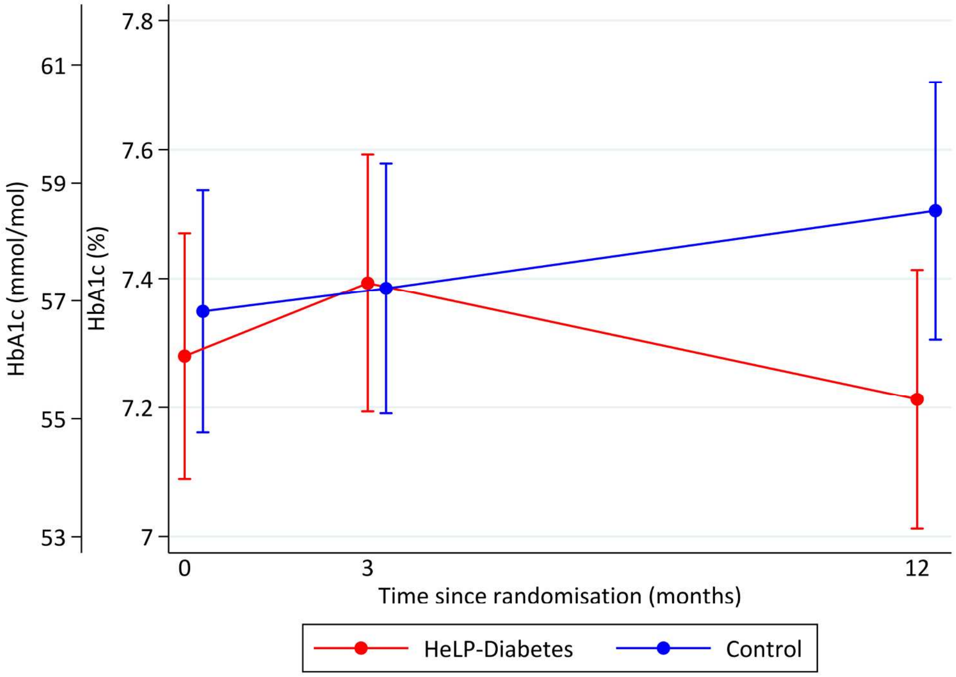

Figure 2: Mean HbA1c (95% confidence interval) over follow up by randomised group using multiple imputation

101x73mm (600 x 600 DPI)

ew only

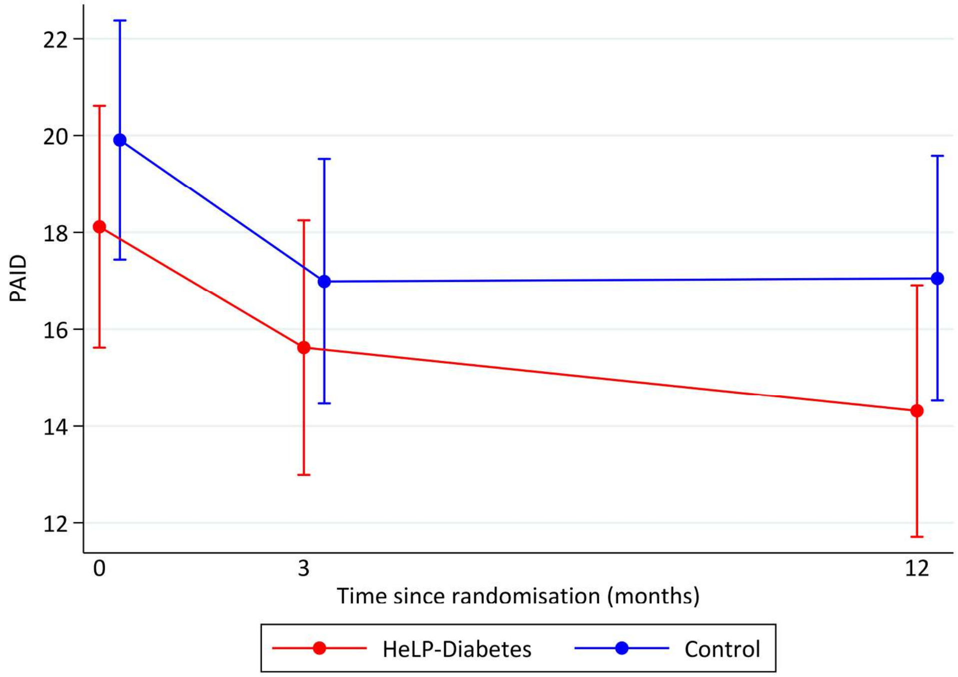

Figure 3: Mean PAID score (95% confidence interval) over follow up by randomised group using multiple imputation.

101x73mm (600 x 600 DPI)

Appendix 1: description of the intervention.[1]

Title: Healthy Living for People with type 2 Diabetes (HeLP-Diabetes): a web-based self-management programme for people with type 2diabetes.

Rationale

The aim of HeLP-Diabetes was to enable people with type 2 diabetes to lead healthier lives and improve overall quality of life. In order to do this, we adopted a strong theoretical framework, which included:

1. The Corbin and Strauss model of living with a long term condition,[2] which determined the overall scope of the intervention;
2. Normalisation Process Theory,[3] to maximise likelihood of successful implementation of the intervention into routine health care
3. A taxonomy of behaviour change techniques,[4] to guide the content of the behaviour change modules;
4. Finally, we worked within the paradigm of evidence-based medicine, ensuring all content was congruent with current best evidence. This applied both to the medical content (e.g. information about treatments) and to the delivery of the intervention (e.g. evidence about maximising acceptability, uptake and adherence).

Self-management of a long term condition is a complex process, which Corbin and Strauss characterised as requiring medical, emotional and role management.[2] Medical management includes behaviour change, such as eating healthily, being physically active, stopping smoking and taking medication. Working with health professionals and the health care system (e.g. keeping appointments, remembering to schedule check-ups or regular monitoring tests) is also included in medical management. Equally important, and often just as challenging for individuals concerned, are emotional management and role management. Emotional management refers to managing the complex negative emotions that result from being diagnosed with a long term condition, such as depression, anger, guilt, shame and a sense of stigma. Role management requires adjusting to the change in biographical narrative, acceptance of the role of "patient", and negotiating inevitable changes in relationships with family, friends and colleagues. This characterisation of the work of self-management provided the overall framework for the content, tone and style of the intervention. As these tasks are intertwined and inter-dependent, we designed the content to address all three tasks in an integrated fashion.

Normalisation Process Theory is a mid-range sociological theory which predicts and explains whether and why certain innovations will be effectively implemented into routine health care and become "normalised", that is, become so much part of routine practice that they disappear from view.[3] It posits that interventions which are easily understood and distinguished from other interventions (coherence), which relevant professionals can see the advantage of compared to current or alternative practice (cognitive participation), which fit well with existing professional relationships and workflows (collective action), and where users are provided with formal or informal feedback and evidence about the impact of the intervention (reflexive monitoring) are more likely to be implemented than those that do not. These constructs guided the development of the intervention by, for example, ensuring HeLP-Diabetes integrated well with existing workflows in general practice.

Effective self-management in diabetes often requires patients to change deep-rooted behaviours, particularly around diet and physical activity. Rather than adopt a single behavioural theory, we opted to design our behaviour change modules around specific

For peer review only - http://bmjopen.bmj.com/site/about/guidelines.xhtml

behaviour change techniques, selecting those with the best evidence base to support their use.[4]

Content

The overall content was broken down into 8 sections:

1. Understanding diabetes (information about the nature and causes of diabetes, and how it affects the body);
2. Staying healthy (motivational material about how to maintain optimal physical and emotional health and the importance of self-management; new behaviour change modules and previously validated programmes for diet, weight loss, physical activity, smoking cessation, moderating alcohol intake, and taking medicines);
3. Treating diabetes (information about medications used in diabetes, including information about indications, side effects and monitoring; importance of managing cardiovascular risk factors as well as glycaemic levels; importance of regular monitoring to prevent retinopathy, neuropathy and nephropathy; and types and roles of different health care professionals involved in caring for people with diabetes);
4. Living and working with diabetes (focus on managing social and work situations, such as shift work, parties, or holidays; impact on relationships, including sexual relationships; and possible impact on emotions and feelings of self-worth);
5. Managing my feelings (self-assessment tools for identifying low mood; cognitive behavioural therapy modules; mindfulness-based approaches);
6. My health record (opportunity to record appointments with health care professionals, results of tests or self-monitoring, with opportunities for graphical displays and feedback);
7. News and Research (updates about latest research or news about diabetes treatment, in depth articles about seminal research papers);
8. Forum and Help (moderated forum; videos of personal stories about diabetes (used with license from health talk online); additional resources, with local resources tailored according to Clinical Commissioning Group (CCG)).

Where possible we aimed to use existing modules with a proven evidence-base. Hence we used, with licence, the entire type 2 diabetes module from Health Talk Online, which contains videos of interviews with people with diabetes from a range of socio-economic and ethnic backgrounds, talking about how they were diagnosed, the impact diabetes has had on their lives and relationships, how they have adapted or come to terms with it, and treatments they have experiences (see www.healthtalk.org). Behaviour change programmes used with license included: POWeR (Positive Online Weight Reduction), a set of 12 modules shown to be effective for managing obesity in primary care[5]; Stop Advisor (smoking cessation)[6] and Down Your Drink (alcohol moderation).[7] New modules were developed for healthy eating, increasing physical activity and taking medication. These used the behaviour change techniques of action planning, goal setting, self-monitoring, reminders and feedback, along with information about the benefits of change. The section on “Managing my feelings” included the *Living Life to the Full* programme[8] – a set of 10 modules based on the principals of cognitive behavioural therapy, specifically adapted for people with type 2 diabetes, and used with license.

Procedures

For peer review only - http://bmjopen.bmj.com/site/about/guidelines.xhtml

HeLP-Diabetes was designed to be used as part of an overall package of care for people with diabetes. Low usage, or non-adherence to internet interventions is well-recognised problem, and our preparatory work with patients and health care professionals indicated that integrating the intervention into routine care was likely to improve uptake and adherence. Hence we made the programme available to registered users only, and encouraged health care professionals to register patients. Once registered, patients could use the programme as much (or as little) as they wanted. There was no prescribed level of use, as our proposed users included patients at all stages of their illness journey, from those newly diagnosed to those who had lived with diabetes for many years. As such, we anticipated that each user would have different needs and priorities, and the programme was designed to allow users to pick and choose sections that were most relevant and beneficial for them personally. There was a limited amount of tailoring. Additional resources and sources of help were tailored by the patient's CCG, but otherwise tailoring was limited to the behaviour change and health record sections where users entered their own goals or data.

Registration and facilitation.

During the trial, registration was performed by a practice nurse. During the registration procedures users were asked to select a password and username. After registration, the nurse was asked to demonstrate the intervention to the patient, and integrate use of the intervention into the patient's personal diabetes care plan by, for example, discussing what goals the patient would like to set for the coming period, and showing the patient how to use the intervention to set goals, monitor progress, and, if desired, arrange for automated reminders by SMS or email to be sent by the programme.

Registration was undertaken in the patient's practice. All subsequent use of the intervention was at any location convenient for the patient with internet access. For most people, we expected this to be at home, or at the home of a relative. However, all users were given information about local services (usually libraries) offering free access to an internet-connected computer.

Encouraging engagement

In addition to the registration and facilitation procedures described above, engagement to the programme was encouraged through regular prompts, provided by email or SMS. Emailed prompts were of two types: a) short (2 – 3 lines) emails with one message and a link to the relevant section of the programme. An example of this type of prompt was a seasonal reminder of the importance of flu vaccination for people with diabetes, with a link to the page of the programme explaining how and why flu vaccination is beneficial, and a reminder that flu vaccination is available free from their general practice. The second type of email prompt was a longer (1 page of A4) newsletter, which summarised recent research or news stories, explained what this meant for patients, and also contained a paragraph or two on specific health promotion – for example, the importance of regular foot care, how to fast safely during Ramadan, or tips for safe overseas travel. These stories and paragraphs also contained links to relevant sections of the programme. SMS prompts were very brief, with one clear message, and a link to the programme. We sent around 3 prompts per month – usually 2 short emails or SMS plus one longer newsletter. Users could opt out of receiving prompts.

For peer review only - http://bmjopen.bmj.com/site/about/guidelines.xhtml

Modifications during the trial

One of the key functions of the programme was to provide up-to-date, evidence-based information. Hence the site was regularly reviewed to ensure all content was up-to-date, evidence-base, and congruent with current NICE guidelines. In practice, this meant small updates each month, with a complete review when the NICE guidelines on management of diabetes were updated.[9]

Assessment of fidelity

Intervention use was assessed through bespoke software which recorded the date, time and pages viewed for each log in by each user. Practice nurses were trained in registration and facilitation procedures, but we were unable to monitor how well they adhered to them.

Availability of the intervention

The intervention is currently available for commissioning from a not-for-profit Community Interest Company, HeLP-Digital. The open access sections of the intervention can be viewed at http://www.helpdigitalcic.org.uk/products

References

1. Hoffmann TC, Glasziou PP, Boutron I, et al. Better reporting of interventions: template for intervention description and replication (TIDieR) checklist and guide. *BMJ (Clinical research ed)* 2014; **348**: g1687.
2. Corbin JM, Strauss A. Unending Work and Care. First ed. San Francisco: Jossey-Bass Inc; 1988.
3. May C, Finch T. Implementation, embedding, and integration: an outline of Normalization Process Theory. *Sociology* 2009; **43**(3): 535-54.
4. Abraham C, Michie S. A taxonomy of behavior change techniques used in interventions. *Health Psychol* 2008; **27**(3): 379-87.
5. Yardley L, Ware LJ, Smith ER, et al. Randomised controlled feasibility trial of a web-based weight management intervention with nurse support for obese patients in primary care. *The international journal of behavioral nutrition and physical activity* 2014; **11**: 67.
6. Brown J, Michie S, Geraghty AW, et al. Internet-based intervention for smoking cessation (StopAdvisor) in people with low and high socioeconomic status: a randomised controlled trial. *The Lancet Respiratory medicine* 2014; **2**(12): 997-1006.
7. Wallace P, Murray E, McCambridge J, et al. On-line Randomized Controlled Trial of an Internet Based Psychologically Enhanced Intervention for People with Hazardous Alcohol Consumption. *PLoSONE* 2011; **6**(3): e14740.
8. Williams C, Association CMH. Living life to the full: Media innovations; 2008.
9. NICE. Type 2 diabetes in adults: Management. NG28. London, 2015.

For peer review only - http://bmjopen.bmj.com/site/about/guidelines.xhtml

TiDIER CHECKLIST.

Item	Line numbers
Provide the name or a phrase that describes the intervention	2,3
Describe any rationale, theory or goal of the elements essential to the intervention	4 – 48
Materials: describe any physical or informational materials used in the intervention, including those provided to participants or used in intervention delivery or in training of the intervention providers	51-90
Procedures: Describe each of the procedures, activities and /or processes used in the intervention, including any enabling or support activities	92 - 137
For each category of intervention provider, describe their expertise, background, and any specific training given.	109
Describe the modes of delivery (such as face-to-face or by some other mechanism, such as internet or telephone) of the intervention, and whether it was provided individually or in a group	2, 123 - 137
Describe the type(s) of location(s) where the intervention occurred, including any necessary infrastructure or relevant features	116 - 120
Describe the number of times the intervention was delivered and over what period of time including the number of sessions, their schedule and their duration, intensity or dose.	98 - 106
If the intervention was planned to be personalised, titrated or adapted, then describe what, why, when and how.	101 - 106
If the intervention was modified during the course of the study, describe the changes (what, why, when and how)	139 - 144
Planned: if intervention adherence or fidelity was assessed, describe how and by whom, and if any strategies were used to maintain or improve fidelity, describe them.	147 - 149
Actual: if intervention adherence or fidelity was assessed, describe the extent to which the intervention was delivered as planned.	147 - 149

For peer review only

Appendix 2: Additional statistical methods and results

Statistical Methods for Multiple Imputation

Multiple Imputation using chained equation was used as the primary method to account for missing data (in both baseline and follow-up data).[1] A set of imputation models were specified, one for each variable with missing data. Each variable was then regressed on all other variables, including completely recorded baseline and follow-up variables and stratified by randomised group. Imputations were performed using predictive mean matching using the five nearest neighbours to the prediction as a set to draw from. The full list of variables considered in the MICE approach is shown in Supplementary Table 1, together with the number of missing values for each variable and time period.

Since only measurements within a 10-14 month window period were used within the main analyses of HbA1c and PAID, the following imputation procedure was implemented for these two co-primary outcomes. Twelve month measurements were subdivided into those that were measured within 10-14 months (the primary outcome variable) and those that were measured outside 10-14 months (a variable used for imputing only). For HbA1c, two additional variables were created for use within the imputation model; 1) the time in days from randomisation that the “12-month” HbA1c measurement was actually taken for values inside of the window period (and set to 365 for measurements taken outside the window period), 2) the time in days from randomisation that the “12-month” HbA1c measurement was actually taken for values outside the window period (and set to missing for measurements taken within the window period). The first of these variables gives the desired time for imputing HbA1c measurements when they are missing, whilst the second gives information on how far outside of the window the actual measurements were taken. Corresponding variables were created for the “12-month” PAID measurement. Finally, two additional variables were created defining the time in days at which HbA1c and PAID were measured at “3-months”. All variables were included within the chained equations and imputed where necessary.

40 imputed datasets were created, the analysis models were fitted to each imputed dataset separately, and the estimates were pooled using Rubin’s rules.

Statistical Methods for causal analyses

The causal analysis proposed attempts to address how the effectiveness of the intervention is mediated through the frequency of website usage. In particular, it is important to understand whether prolonged usage of the website modifies the efficacy of the intervention. Since website usage is measured post-randomisation a naïve analysis of correlating usage with outcomes in the intervention group may give biased and misleading results, since there may be unmeasured confounders also correlated with the outcomes that distinguish the motivated users who regularly log-in from the less motivated ones. Causal analyses using instrumental variables (IV) were therefore used to determine the effect of website usage on outcomes. This approach preserves randomisation (i.e. provides a comparison independent of observed and unobserved confounders).

“Usage” is defined as the proportion of follow-up (rescaled as no. days in a year) that the HeLP-Diabetes website is accessed. It was assumed that the efficacy of the intervention is zero for individuals who never log-in (the exclusion restriction assumption). Website usage in the control group was ignored in the model as it was assumed that the control website was unlikely to be effective. (NB. usage statistics were collected for the comparator website but were not used in these analyses).

For peer review only

This main underlying assumption of the causal analysis is that the effect of randomisation to the HeLP-Diabetes intervention on 12-month outcomes occurs only through use of the website (Supplementary Figure 1; Z is randomised intervention, WU is website-usage and Y is 12-month outcome, e.g. HbA1c or PAID). This relies of the “exclusion restriction” assumption that the HeLP Diabetes intervention has no effect when usage is zero (i.e. for individuals who never log-in). Hence randomisation is assumed to be an instrumental variable.

Figures 4 and 5 show the estimated causal effects of HeLP Diabetes on HbA1c and PAID at 12-months, respectively, by level of website usage (the “exclusion restriction” assumption leads to zero efficacy at zero usage). To determine the casual estimate of efficacy for a “high-usage” group, the individual predicted efficacy was calculated for each individual greater than or equal to the median usage of 4 days (shown as the red boxes in the histograms in Figures 4 and 5) and the average efficacy for this group was obtained. The mean usage in the “high-usage” group was 18 days.

Supplementary Figure 1. Causal model assuming no direct effect of intervention (complete mediation through website usage).

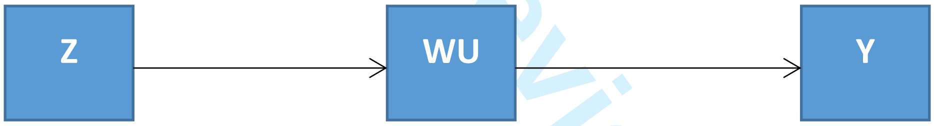

In this causal model, randomisation (Z) is the instrumental variable, which acts on the outcome (Y) exclusively through website usage (WU).

For peer review only

Supplementary Figure 2: Efficacy of HeLP-Diabetes on HbA1c at 12-months based on number of days usage, superimposed by the distribution of usage observed in the HeLP-Diabetes group. The bars shown in red highlight the "high-usage" group that have usage greater than or equal to the median of 4 days.

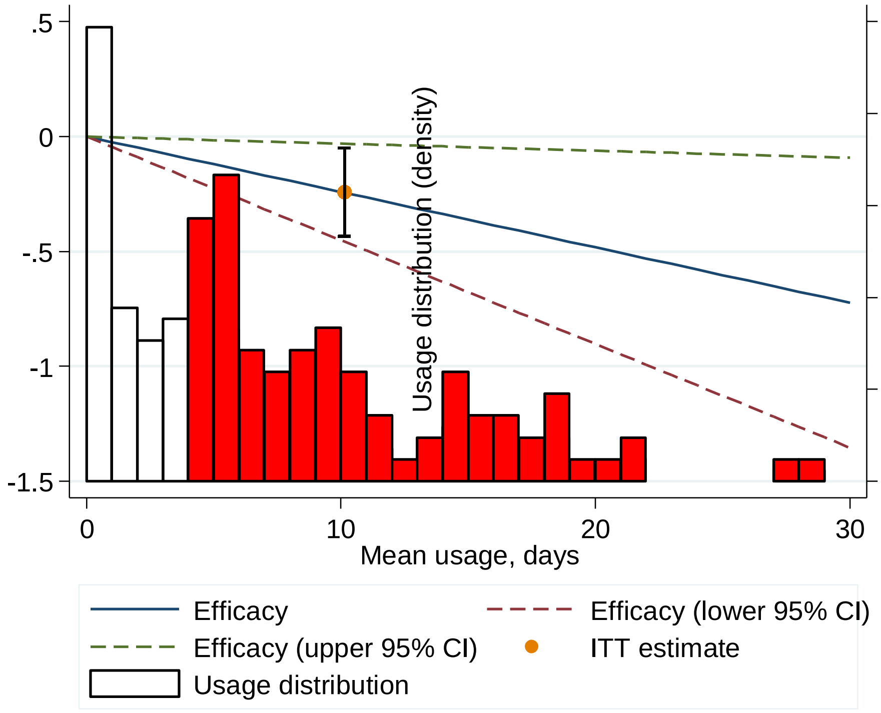

Supplementary Figure 3. Efficacy of HeLP-Diabetes on PAID score at 12-months based on number of days usage, superimposed by the distribution of usage observed in the HeLP-Diabetes group. The bars shown in red highlight the "high-usage" group that have usage greater than or equal to the median of 4 days.

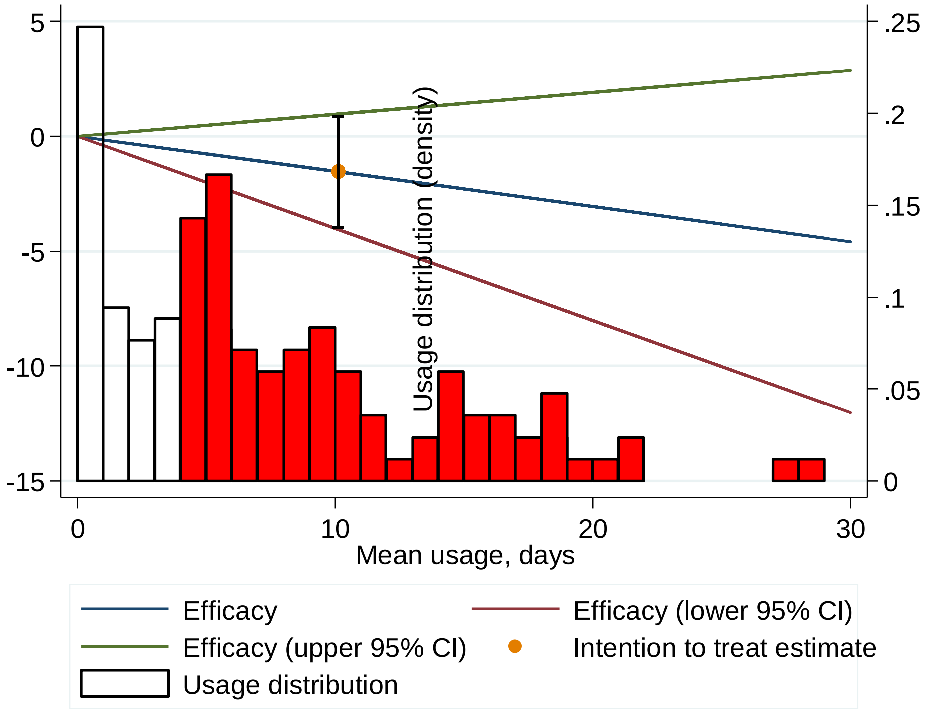

For peer review only

Supplementary Figure 4. Means and 95% CIs of HbA1c over time by randomised group and completers vs. non-completers, after multiple imputation of non-completers follow-up

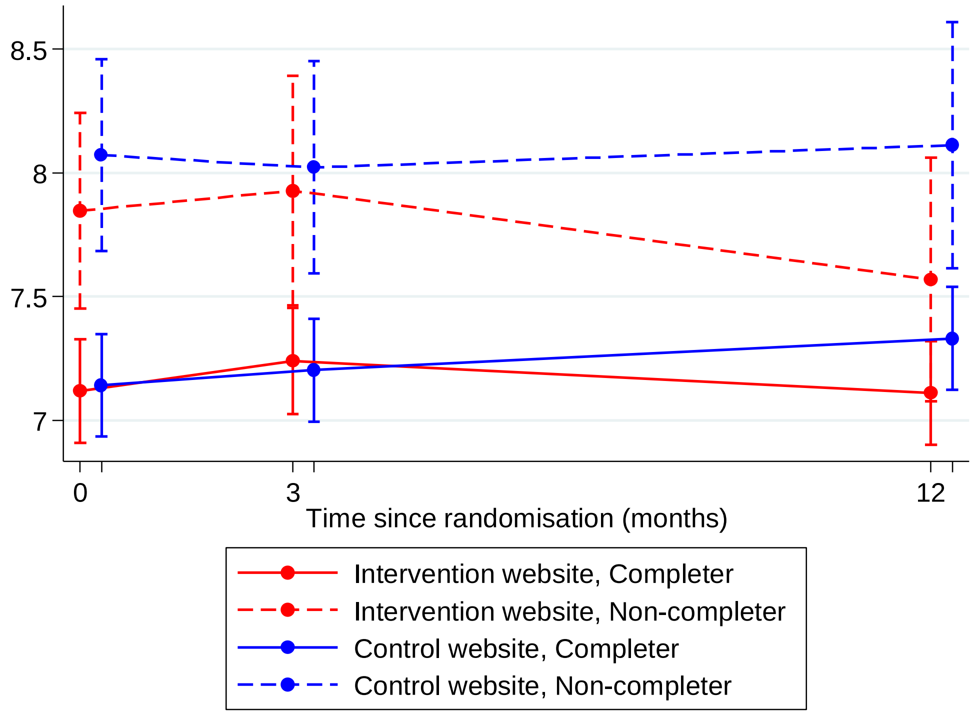

view only

Supplementary Table 1. List of variables imputed and amount of missing data at baseline, 3 months and 12 months.

Variable	Number of missing values		
	Baseline	3 months	12 months
HbA1c	5	64	83 10-14months †
			347 Outside 10-14months †
PAID	0	54	53 10-14months †
			358 Outside 10-14months †
Systolic Blood Pressure	0	57	68
Diastolic Blood Pressure	0	57	68
Body Mass Index	2	58	69
Total cholesterol	2	68	75
HDL cholesterol	12	73	76
Completion of 9 essential processes	69	-	69
HADS	0	74	107
DMSES	0	73	109
DTSQ	0	77	109
Age	0	-	-
Sex	0	-	-
Duration of diabetes	4	-	-
History of cardiovascular disease	0	-	-
Attending any other self-management class	0	-	-
Smoking status	0	-	-
No of visits to the website	-	-	0
No of webpages visited	-	-	105
Average time per visit	-	-	105

* Partial mean matching performed using 5 nearest neighbours

† 12-month measurements were subdivided into those that were measured within 10-14 months (primary outcome variable) and those that were measured outside 10-14 months (variable used for imputing only).

For peer review only

Supplementary Table 2: Descriptive statistics of 3-month variables by randomised group. Mean (SD) unless otherwise specified.

	HeLP-Diabetes	Control	p-value*	N missing
**Clinical Measures**				
Systolic blood pressure (mmHg)	133 (16)	134 (17)	0.350	57
Diastolic blood pressure (mmHg)	76 (10)	75 (9)	0.699	57
Total cholesterol (mmol/l)	4.17 (0.97)	4.16 (1.04)	0.900	68
HDL-C (mmol/l)	1.29 (0.37)	1.24 (0.37)	0.356	73
Total cholesterol /HDL-C ratio	3.48 (1.15)	3.52 (1.09)	0.570	74
HbA1c (%)	7.34 (1.48)	7.34 (1.24)	0.323	64
Body mass index (Kg/m^2)	30.0 (5.4)	30.0 (5.6)	0.545	58
**Questionnaires / scores**				
PAID (0-100)	15.2 (15.2)	16.7 (17.1)	0.492	54
HADS (0-42)	8.84 (6.54)	8.86 (7.45)	0.603	74
Anxiety scale (0-21)	4.75 (3.88)	5.07 (4.24)	0.639	74
Depression scale (0-21)	4.10 (3.30)	3.80 (3.59)	0.205	74
DMSES (0-150)	103.9 (32.2)	104.9 (32.7)	0.795	73
DTSQ (0-48)	32.8 (6.9)	32.9 (6.3)	0.715	77

* Wilcoxon rank-sum test

For peer review only

Supplementary Table 3. Effect of HeLP-Diabetes vs. Control on 12-month primary outcomes, HbA1c and PAID. Results from primary analyses and six sensitivity analyses

	HbA1c, %	
	Number individuals	mean difference (95% CI) HeLP-Diabetes - Control
Primary Analysis Multiple Imputation of both baseline and outcomes	374	-0.242 (-0.435, -0.049); p=0.014
Sensitivity Analysis 1: (multiple imputation of baseline covariates only)	291	-0.214 (-0.390, -0.038); p=0.017
Sensitivity Analysis 2: (complete cases) ⱡ	284	-0.207 (-0.385, -0.028); p=0.023
Sensitivity Analysis 3: (complete cases) ǂ	186	-0.220 (-0.416, -0.025); p=0.027
Sensitivity Analysis 4: (non-contaminated cases) ¥	370	-0.225 (-0.418, -0.032); p=0.023
Sensitivity Analysis 5: Linear model excluding centre*	374	-0.242 (-0.438, -0.047); p=0.015
Sensitivity Analysis 6: Unadjusted model (complete-cases)	291	-0.220 (-0.483, 0.043); p=0.100
	PAID	
	Number individuals	mean difference (95% CI) HeLP-Diabetes - Control
Primary Analysis Multiple Imputation	374	-1.54 (-3.94, 0.87); p=0.209
Sensitivity Analysis 1: (multiple imputation of baseline covariates only)	321	-1.31 (-3.65, 1.03); p=0.274
Sensitivity Analysis 2: (complete cases) ⱡ	317	-1.29 (-3.66, 1.08); p=0.285
Sensitivity Analysis 3: (complete cases) ǂ	65	-2.22 (-6.35, 1.91); p=0.292
Sensitivity Analysis 4: (non-contaminated cases) ¥	370	-1.57 (-3.99, 0.85); p=0.204
Sensitivity Analysis 5: Linear model excluding centre**	374	-1.54 (-3.98, 0.90); p=0.215
Sensitivity Analysis 6: Unadjusted model (complete-cases)	321	-2.38 (-6.02, 1.26); p=0.199

ⱡ excluding outcomes outside 10-14 months post-randomisation and those missing baseline covariates

ǂ excluding outcomes outside 11-13 months post-randomisation and those missing baseline covariates

¥ Excluding patients who were suspected to have been exposed to the alternative intervention

* likelihood ratio test for including centre in the model as a fixed effect; p=0.617 (HbA1c)

** likelihood ratio test for including centre in the model as a fixed effect; p=0.357 (PAID)

For peer review only

For peer review only

Supplementary Table 4. Effect of subgroups on the effectiveness of HeLP-Diabetes in reducing HbA1c and PAID.

Subgroup	HbA1c, %			
	Number individuals	mean difference (95% CI) HeLP-Diabetes - Control		Interaction p-value*
		Complete Case ǂ	Multiple Imputation	
Baseline HbA1c				0.458
<7.5%	254/369	-0.15 (-0.41, 0.11)	-0.14 (-0.39, 0.12)	
≥7.5%	115/369	-0.51 (-0.94, -0.07)	-0.51 (-0.93, -0.09)	
Duration of diabetes				0.425
<6.9 yrs	185/370	-0.34 (-0.59, -0.08)	-0.30 (-0.57, -0.03)	
≥6.9 yrs	185/370	-0.07 (-0.32, 0.18)	-0.18 (-0.45, 0.09)	
	PAID			
Baseline PAID				0.066
<12	181/374	-0.3 (-4.6, 4.1)	-0.1 (-4.2, 4.1)	
≥12	193/374	-3.6 (-7.8, 0.7)	-3.9 (-8.2, 0.4)	
Duration of diabetes				0.004
<6.9 yrs	185/370	-2.9 (-6.3, 0.4)	-3.6 (-7.0, -0.1)	
≥6.9 yrs	185/370	0.3 (-3.0, 3.7)	0.4 (-3.1, 3.9)	

ǂ excluding outcomes outside 10-14 months post-randomisation and those missing baseline covariates

* p-value for interaction based on a test of linear trend with the continuous variable.

For peer review only

Reference List

1. White IR, Royston P, Wood AM. Multiple imputation using chained equations: Issues and guidance for practice. *Statistics in medicine* 2011; **30**(4): 377-99.

CONSORT 2010 checklist of information to include when reporting a randomised trial*

Section/Topic	Item No	Checklist item	Reported on page No
**Title and abstract**			
	1a	Identification as a randomised trial in the title	1
	1b	Structured summary of trial design, methods, results, and conclusions (for specific guidance see CONSORT for abstracts)	2
**Introduction**			
Background and objectives	2a	Scientific background and explanation of rationale	4
	2b	Specific objectives or hypotheses	4
**Methods**			
Trial design	3a	Description of trial design (such as parallel, factorial) including allocation ratio	4
	3b	Important changes to methods after trial commencement (such as eligibility criteria), with reasons	4
Participants	4a	Eligibility criteria for participants	4
	4b	Settings and locations where the data were collected	4
Interventions	5	The interventions for each group with sufficient details to allow replication, including how and when they were actually administered	5 + Appendix 1
Outcomes	6a	Completely defined pre-specified primary and secondary outcome measures, including how and when they were assessed	6
	6b	Any changes to trial outcomes after the trial commenced, with reasons	N/a
Sample size	7a	How sample size was determined	6-7
	7b	When applicable, explanation of any interim analyses and stopping guidelines	N/A
Randomisation:			
Sequence generation	8a	Method used to generate the random allocation sequence	5
	8b	Type of randomisation; details of any restriction (such as blocking and block size)	5
Allocation concealment mechanism	9	Mechanism used to implement the random allocation sequence (such as sequentially numbered containers), describing any steps taken to conceal the sequence until interventions were assigned	5
Implementation	10	Who generated the random allocation sequence, who enrolled participants, and who assigned participants to interventions	5
Blinding	11a	If done, who was blinded after assignment to interventions (for example, participants, care providers, those	5

For peer review only - http://bmjopen.bmj.com/site/about/guidelines.xhtml

			5
		assessing outcomes) and how	6
	11b	If relevant, description of the similarity of interventions	
Statistical methods	12a	Statistical methods used to compare groups for primary and secondary outcomes	7
	12b	Methods for additional analyses, such as subgroup analyses and adjusted analyses	7 + Appendix 2
**Results**			
Participant flow (a diagram is strongly recommended)	13a	For each group, the numbers of participants who were randomly assigned, received intended treatment, and were analysed for the primary outcome	7 + Figure 1
	13b	For each group, losses and exclusions after randomisation, together with reasons	Figure 1
Recruitment	14a	Dates defining the periods of recruitment and follow-up	7
	14b	Why the trial ended or was stopped	N/A
Baseline data	15	A table showing baseline demographic and clinical characteristics for each group	Table 1
Numbers analysed	16	For each group, number of participants (denominator) included in each analysis and whether the analysis was by original assigned groups	Figure 1
Outcomes and estimation	17a	For each primary and secondary outcome, results for each group, and the estimated effect size and its precision (such as 95% confidence interval)	8, Table 2, Figures 2 & 3
	17b	For binary outcomes, presentation of both absolute and relative effect sizes is recommended	N/A
Ancillary analyses	18	Results of any other analyses performed, including subgroup analyses and adjusted analyses, distinguishing pre-specified from exploratory	8, Supplementary Table 4
Harms	19	All important harms or unintended effects in each group (for specific guidance see CONSORT for harms)	9
**Discussion**			
Limitations	20	Trial limitations, addressing sources of potential bias, imprecision, and, if relevant, multiplicity of analyses	9 - 10
Generalisability	21	Generalisability (external validity, applicability) of the trial findings	9 - 10
Interpretation	22	Interpretation consistent with results, balancing benefits and harms, and considering other relevant evidence	9 - 10
**Other information**			
Registration	23	Registration number and name of trial registry	7
Protocol	24	Where the full trial protocol can be accessed, if available	Reference 17, page 22
Funding	25	Sources of funding and other support (such as supply of drugs), role of funders	11

*We strongly recommend reading this statement in conjunction with the CONSORT 2010 Explanation and Elaboration for important clarifications on all the items. If relevant, we also recommend reading CONSORT extensions for cluster randomised trials, non-inferiority and equivalence trials, non-pharmacological treatments, herbal interventions, and pragmatic trials. Additional extensions are forthcoming: for those and for up to date references relevant to this checklist, see www.consort-statement.org.

For peer review only - http://bmjopen.bmj.com/site/about/guidelines.xhtml
